# Plasma enzymatic activity, proteomics and peptidomics in COVID-19-induced sepsis: A novel approach for the analysis of hemostasis

**DOI:** 10.3389/fmolb.2022.1051471

**Published:** 2023-01-11

**Authors:** Fernando Dos Santos, Joyce B. Li, Nathalia Juocys, Rafi Mazor, Laura Beretta, Nicole G. Coufal, Michael T. Y. Lam, Mazen F. Odish, Maria Claudia Irigoyen, Anthony J. O’Donoghue, Federico Aletti, Erik B. Kistler

**Affiliations:** ^1^ Department of Anesthesiology, School of Medicine, University of California, San Diego, CA, United States; ^2^ Department of Bioengineering, University of California, San Diego, CA, United States; ^3^ Instituto do Coração, Hospital das Clínicas, Faculdade de Medicina, Universidade de São Paulo (InCor-FMUSP), São Paulo, Brazil; ^4^ Skaggs School of Pharmacy and Pharmaceutical Sciences, University of California, San Diego, CA, United States; ^5^ Department of Pediatrics, School of Medicine, University of California, San Diego, CA, United States; ^6^ Department of Medicine, School of Medicine, University of California, San Diego, CA, United States; ^7^ Instituto de Ciência e Tecnologia, Universidade Federal de São Paulo, São Josê dos Campos, Brazil; ^8^ Department of Anesthesiology and Critical Care, VA San Diego Healthcare System, San Diego, CA, United States

**Keywords:** COVID-19, sepsis, proteolysis, enzymatic activity, peptidomics, proteomics

## Abstract

**Introduction:** Infection by SARS-CoV-2 and subsequent COVID-19 can cause viral sepsis. We investigated plasma protease activity patterns in COVID-19-induced sepsis with bacterial superinfection, as well as plasma proteomics and peptidomics in order to assess the possible implications of enhanced proteolysis on major protein systems (e.g., coagulation).

**Methods:** Patients (=4) admitted to the intensive care units (ICUs) at the University of California, San Diego (UCSD) Medical Center with confirmed positive test for COVID-19 by real-time reverse transcription polymerase chain reaction (RT-PCR) were enrolled in a study approved by the UCSD Institutional Review Board (IRB# 190699, Protocol #20-0006). Informed consent was obtained for the collection of blood samples and de-identified use of the data. Blood samples were collected at multiple time points and analyzed to quantify a) the circulating proteome and peptidome by mass spectrometry; b) the aminopeptidase activity in plasma; and c) the endopeptidase activity in plasma using fluorogenic substrates that are cleaved by trypsin-like endopeptidases, specific clotting factors and plasmin. The one patient who died was diagnosed with bacterial superinfection on day 7 after beginning of the study.

**Results:** Spikes in protease activity (factor VII, trypsin-like activity), and corresponding increases in the intensity of peptides derived by hydrolysis of plasma proteins, especially of fibrinogen degradation products and downregulation of endogenous protease inhibitors were detected on day 7 for the patient who died. The activity of the analyzed proteases was stable in survivors.

**Discussion:** The combination of multiomics and enzymatic activity quantification enabled to i) hypothesize that elevated proteolysis occurs in COVID-19-induced septic shock with bacterial superinfection, and ii) provide additional insight into malfunctioning protease-mediated systems, such as hemostasis.

## 1 Introduction

The Coronavirus disease (COVID-19) pandemic is caused by viral infection with the coronavirus SARS-CoV-2. Affecting every organ of the body following initial infection of the respiratory system, this highly contagious virus has contributed to more than 6 million confirmed deaths to date. Despite the emphasis on COVID-19 research, our fundamental understanding of the disease remains incomplete and therapeutic options are limited (Osuchowski et al., 2020; Wiersing et al., 2020; Alhazzani et al., 2021; Chalmers et al., 2021; Lopes-Pacheco et al., 2021; Osuchowski et al., 2021; Welte et al., 2021). Frequent and fatal complications arising from SARS-CoV-2-induced sepsis include cytokine storm and coagulopathies (Iba et al., 2020; Tang et al., 2020). Less frequent (Garcia-Vidal et al., 2021), but not less dangerous, bacterial superinfections make the clinical treatment of COVID-19 patients in the intensive care unit (ICU) even more challenging, especially because of the unclear underlying pathology and interplay between viral and bacterial infection and their clinical implications (Lazzaroni et al., 2021).

We have previously shown, in both animal and human studies, that dysregulated proteolysis is a possible, additional pathological mechanism triggered by circulatory shock, including septic shock following bacterial infection (Aletti et al., 2016a; Aletti et al., 2016b; Bauzá-Martinez et al., 2018; Aletti et al., 2022). Several protease-mediated systems, such as the coagulation and complement systems, are known to be compromised in critical illness. To better understand the possible relationship between systemic proteolysis occurring in shock, including COVID-19-induced septic shock, and the malfunction of major protease-mediated systems, we have developed a complex, multimodal approach to assess protease activity and proteolysis in plasma, consisting of quantitative protease activity assays and mass spectrometry-based multi-omics analyses of the circulating proteome and peptidome. The combined use of these techniques has shown promise in the ability to i) estimate *in vivo* proteolysis in plasma as a marker of ongoing systemic proteolysis during shock (Aletti et al., 2016b; Bauzá-Martinez et al., 2018), and ii) detect circulating peptidase activity (Maffioli et al., 2020).

The broad hypothesis of this study was that a complex description of plasma proteins and peptides and of the activity of some of the most important circulating enzymes can be leveraged as a tool to shed light on the progression of COVID-19 and the risk for fatal complications, especially in the presence of bacterial superinfection and diagnosis of septic shock. In particular, we postulated that these measurements, which are not part of the clinical routine, would provide important pathophysiological information on the COVID-19-induced alterations of an all-important proteolytically controlled system such as hemostasis.

The specific goal was to propose a complex, multi-omics and multidata approach for the longitudinal description of the proteome, peptidome and the activity of several plasma peptidases able to assess: 1) the patterns of protease activity in the plasma of COVID-19 patients following admission to the ICU and their association with the development of bacterial superinfection by means of quantitative analysis of enzymatic activity, and 2) the implications of dysregulated proteolysis on important protein systems, such as hemostasis mediated both by coagulation factors and proteases involved in fibrinolysis, by analysis of plasma proteomics and peptidomics during the ICU stay of the participants.

## 2 Materials and methods

### 2.1 Clinical measurements

This was a prospective observational study. The University of California, San Diego Institutional Review Board (IRB) provided approval of the study conduct (IRB# 190699, Protocol #20-0006); informed consent was obtained for the collection of blood samples and de-identified use of the data. A convenience sample of patients (*n* = 4) admitted to the Intensive Care Units (ICUs) at the University of California, San Diego Medical Center with confirmed positive test for COVID-19 by real-time reverse transcription polymerase chain reaction (RT-PCR) was enrolled in the study. De-identified demographic and prior health history data were obtained. Three healthy subjects (male, age 35–56) were enrolled as a control group for protease activity assays. Standard laboratory measurements as necessary for optimal patient care were obtained by the UC San Diego Medical Center for Advanced Laboratory Medicine (CALM) laboratories. All non-standard laboratory assays were conducted off-site in academic research laboratories (protease activity measurements) and in the Biomolecular and Proteomics Mass Spectrometry Facility (mass spectrometry) at the University of California, San Diego, with investigators blinded to patient clinical course and outcomes. Patient blood samples (3–10 mL) were collected in sodium heparin (BD Vacutainer, REF: 366480) tubes. To prevent coagulation, the tubes were inverted 10 times prior to transport at room temperature. Blood was processed within 4 h of collection and kept at room temperature throughout the protocol. PolymorphprepTM was added to collected samples and centrifuged at 500 g × 30 min to separate plasma from white blood cells per manufacturer’s instructions (Progen) and transferred to new Eppendorf tubes and centrifuged at ×3,731 g for 5 min to remove any cellular debris. The supernatant was transferred to new tubes and flash frozen in dry ice and 95% ethanol. Plasma was aliquoted and stored at −80°C for further analysis. The protocol for the preprocessing, storage and subsequent analysis of the samples *via* mass spectrometry (see Section 2.3) ensured that all the samples would be analyzed under the same conditions and to avoid possible bias effects due to sample processing. The clinical data acquisition and blood sample collection started on the day following protocol study consent (usually day of ICU admission) and was repeated on the following day and every other day thereafter until patient discharge or death.

### 2.2 Plasma enzymatic activity

To test enzymatic activity, a variety of fluorogenic substrates was selected, with a specific focus on the activity of endopeptidases such as trypsin-like enzymes, clotting factors (thrombin, factors VII, IX and X), fibrinolytic enzymes (e.g., plasmin) and aminopeptidases. All plasma samples were processed and analyzed simultaneously for each substrate, including healthy control plasma. Briefly, a 384-well plate was used to combine 15 μL of plasma (1/50 final dilution in PBS, pH = 7.4) with 15 μL of each substrate (5 μM in PBS) in triplicate wells and immediately measured for activity with a spectrophotometer (FilterMax F5 Multi-Mode, Molecular Devices) with excitation of 360 nm and emission of 460 nm. Wells were read every 2 min and 45 s for 4 h and 50 min thereby generating fluorescent activity progress curves that each contain 100 data points. Fluorogenic activity was reported as relative fluorescence units per second using the slope from the progress curve where the *r* ^2^ value was higher than .98. The activity was corrected by the dilution factor. Results shown are plotted as the percentage of enzymatic activity relative to the average of healthy control plasma samples on a log2 scale, so that the large intersubject variability is appropriately taken into consideration. The peptide sequences used in this assay were chosen as substrates for enzymes involved in the regulation of coagulation and hemostasis, tryptic-like endopeptidases and aminopeptidases. (Table 1).

**TABLE 1:** Substrates for enzymatic activity assay.

Amino acid sequence	Protease
z-Arg-Arg-Leu-Arg-AMC	Trypsin-like endopeptidase
N-Benzoyl-Phe-Val-Arg-AMC	Trypsin-like endopeptidase
z-Phe-Arg-AMC	Trypsin-like endopeptidase
Methionine Aminopeptidase-AMC	Methionine Aminopeptidase
z-Gly-Gly-Arg-AMC	Thrombin
Boc-Val-Pro-Arg-AMC	Factor VII
Boc-Glu(OBzl)-Gly-Arg-AMC	Factor IX
Boc-Ile-Glu-Gly-Arg-AMC	Factor X
D-Ala-Leu-Lys-AMC	Plasmin

Ala, alanine; AMC, 7-amino-4-methylcoumarin; Arg, arginine; Boc, tert-butyloxycarbonyl; Glu, glutamate; Glu(OBzl), glutamic acid 5-benzyl ester; Gly, glycine; Ile, isoleucine; Leu, leucine; Lys, lysine; Phe, phenylalanine; Pro, proline; Tyr, tyrosine; Val, valine; z, Benzyloxycarbonyl

### 2.3 Proteomics and peptidomics analysis of plasma *via* mass spectrometry

All plasma samples from the COVID-19 patients were subjected to a series of protein extraction and pretreatment steps prior to mass spectrometry. Briefly, for proteomics measurements, proteins were precipitated from the plasma samples, treated with denaturing, reducing, and alkylating reagents, and enzymatically digested with trypsin. To quantify peptides in plasma, high molecular weight proteins were removed following addition of methanol (without any tryptic digestion step in order to avoid *ex vivo* protein hydrolysis); the remaining peptides (exclusively generated by *in vivo* proteolysis) were quantified by tandem mass spectrometry (LC-MS/MS) using nano-spray ionization with a Orbitrap fusion Lumos hybrid mass spectrometer (Thermo Fisher Scientific). The sample preparation procedure both for proteomics and peptidomics and the setting of the mass spectrometry parameters followed established and previously described protocols (Courelli et al., 2021). PEAKS Studio 8.5 (Bioinformatics Solutions, Inc.) proteomics software was used for protein identification and quantification.

For the purposes of this study, the analysis of the circulating proteome and peptidome focused on three main groups of proteins, according to their function: 1) coagulation, e.g. clotting factors and fibrinogen; 2) complement system and proteins involved in the immune response and inflammation, e.g. complement factors, vascular endothelial growth factor receptor 2 (VEGF2) and C-reactive protein, and 3) endogenous protease inhibitors, e.g., serpins such as trypsin and chymotrypsin inhibitors, thrombin and plasmin inhibitors, C1 inhibitors and heparin cofactor II.

For proteome analysis, we used the log_2_ ratio of each individual intensity measurement to the average of all sample intensities for each protein. Heat maps for the three protein function groups described above were generated by Morpheus (https://software.broadinstitute.org/morpheus). Identified proteins were hierarchically clustered with the one minus Pearson’s correlation metric and average linkage method for each protein group. Columns were sorted by patient and ordered by day of sample collection. For peptidomic analysis, the degree of overall proteolysis was estimated by the sum of all fragment intensities in a sample. Similarly, the proteolysis of a single protein was estimated by the sum of the intensities of the peptides generated by that protein. An online web tool, Peptigram (@Bioware—© University College Dublin) (Manguy et al., 2017) was used to build cleavage maps representative of the number of peptides generated by a parent protein, abundance and length of each amino acid sequence, and cleavage site on the parent protein of origin.

## 3 Results

Patient characteristics are shown in Table 2.

**TABLE 2:** Patient characteristics.

	Patient 1	Patient 2	Patient 3	Patient 4
Age	57	47	88	29
BMI	29.40	23.66	32.14	30.19
Male sex	Y	Y		Y
Ethnicity (Hispanic)	Y		Y	Y
Survived	Y	Y		Y
Comorbidities				
COPD				
Chronic kidney disease				
Coronary artery disease				
Hypertension		Y	Y	Y
Diabetes				
Rheumatologic disease				Y
Admit to ICU from ED or Ward	Ward	ED	Ward	ED
ICU length of stay	17	3	16	9
Hospital length of stay	24	8	18	11
Self-described fitness level upon admission	Fit	Fit	Well	Fit
Modified rankin score on admission	No symptoms	No symptoms	No symptoms	No significant disability
Treatment in ICU				
Maximum SOFA score	9	3	8	9
Required pressors	Y		Y	Y
ARDS diagnosis	Y		Y	Y
Presence of another infection			Y	
Antibiotic therapy				
Azithromycin	Y	Y	Y	Y
Ceftriaxone	Y	Y		Y
Meropenem			Y	
Vancomycin			Y	Y
Piperacillin/Tazobactam			Y	Y

The four patients were comprised of three males and one female and ranged in age from 29 to 88 years. Reflecting the overall demographics of the COVID-19 epidemic in San Diego, three of the subjects were Hispanic. One patient (88-year-old female), who will be referred to as “Patient 3” in the following, was diagnosed with bacterial superinfection and subsequent sepsis on day 7 and died on day 16 of her ICU stay. The remainder (Patients 1, 2 and 4) were discharged from the ICU with a mean ICU length-of-stay for all subjects of 11.25 ± 6.55 days and mean hospital stay of 15.25 ± 7.18 days. The only major co-morbidity reported was hypertension in three subjects and rheumatoid arthritis in Patient 4. Body mass index (BMI) was 28.84 ± 3.65 kg/m^2^ (range: 29.4–32.14 kg/m^2^) and self-described baseline health was either “fit” or “well.” All patients were initially treated with azithromycin. The three surviving subjects, who did not present with a bacterial superinfection, were also treated with ceftriaxone. Patient 3 was treated with piperacillin/tazobactam after positive culture identification (Pseudomonas aeruginosa in urine and Enterobacter aerogenes pneumonia), and later with meropenem and vancomycin, after clinical failure to respond to infection.

The three more critically ill patients of the group (Patients 1, 3 and 4) were intubated following diagnosis of Acute Respiratory Distress Syndrome (ARDS) and remained on mechanical ventilation for at least some duration of their ICU stay. These patients also required vasopressor agents to maintain hemodynamic stability. As regards other therapeutic interventions, none of these patients was systematically anti-coagulated, and at the time of their admission to the ICU and recruitment for this study (early in the COVID-19 pandemic), there was no recommendation yet resulting in widespread use of steroids and anti-viral therapies. Further details as to the ICU course and treatment of these patients are available in Supplementary File S1.

**FIGURE 1:**
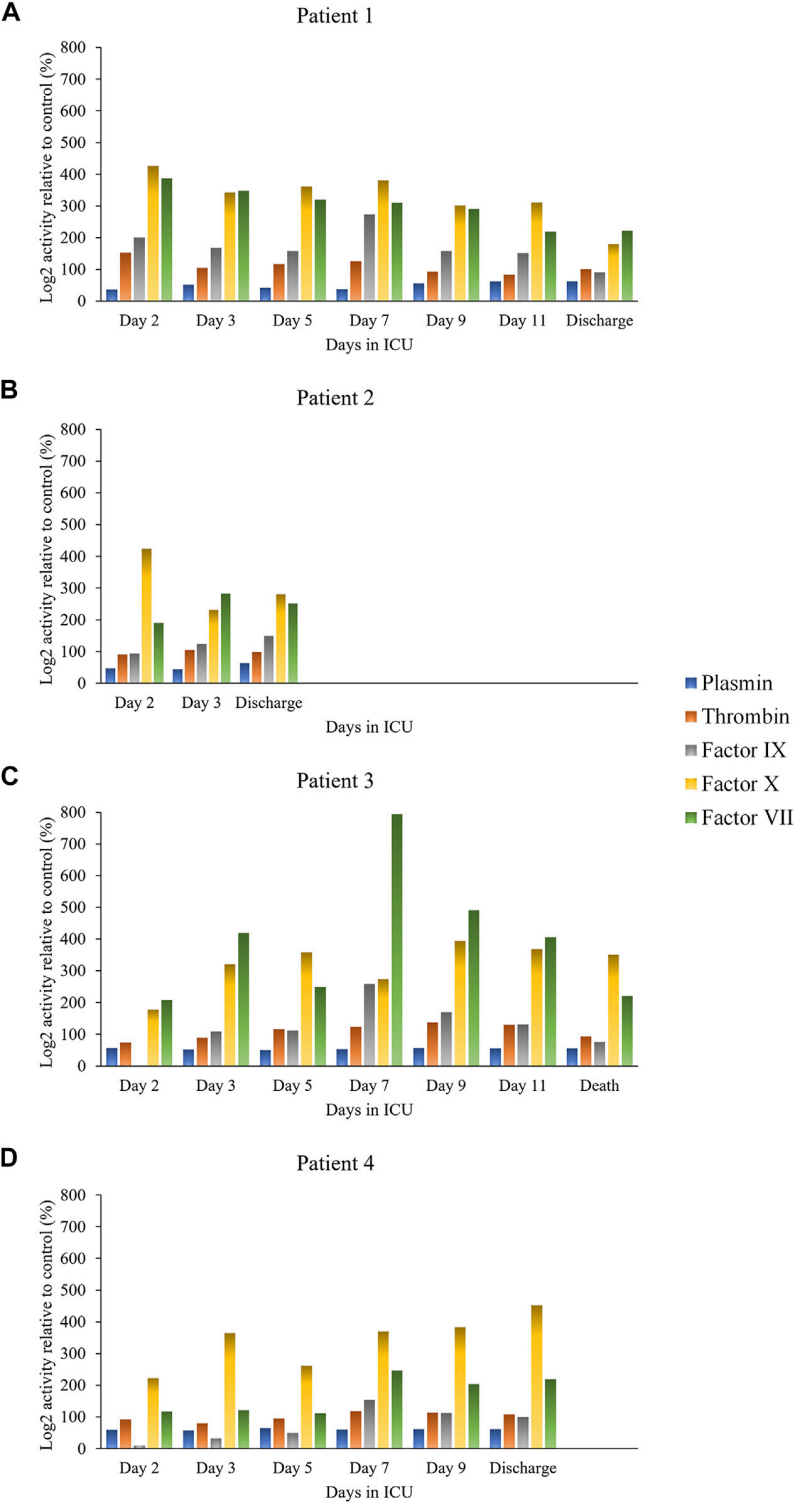
Individual activity of clotting factors and plasmin over time for each patient: Patient 1 **(A)**, Patient 2 **(B)**, Patient 3 **(C)**, Patient 4 **(D)**.

**FIGURE 2:**
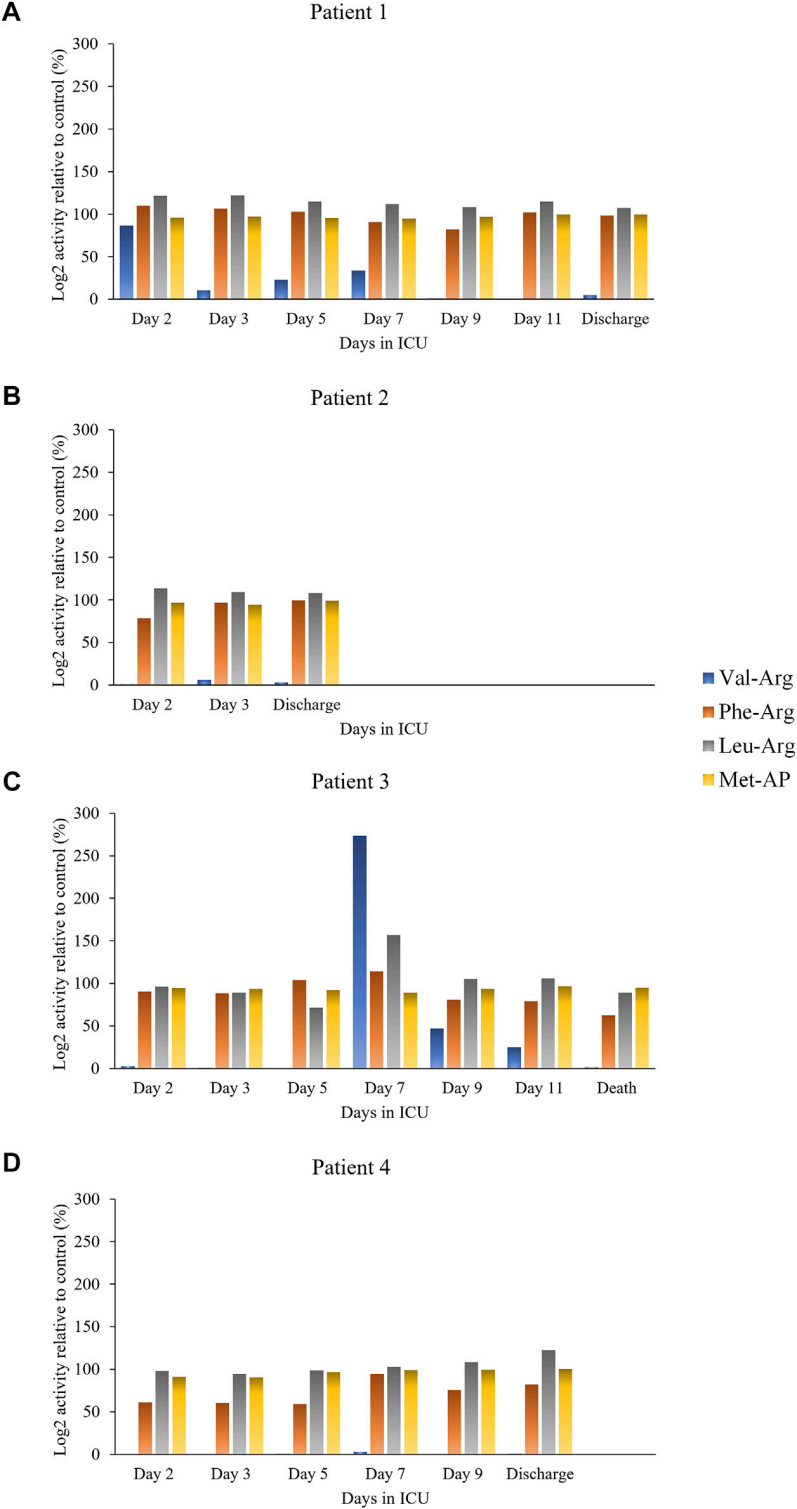
Individual trypsin-like and aminopeptidase activity over time for each patient: Patient 1 **(A)**, Patient 2 **(B)**, Patient 3 **(C)**, Patient 4 **(D)**.

**FIGURE 3:**
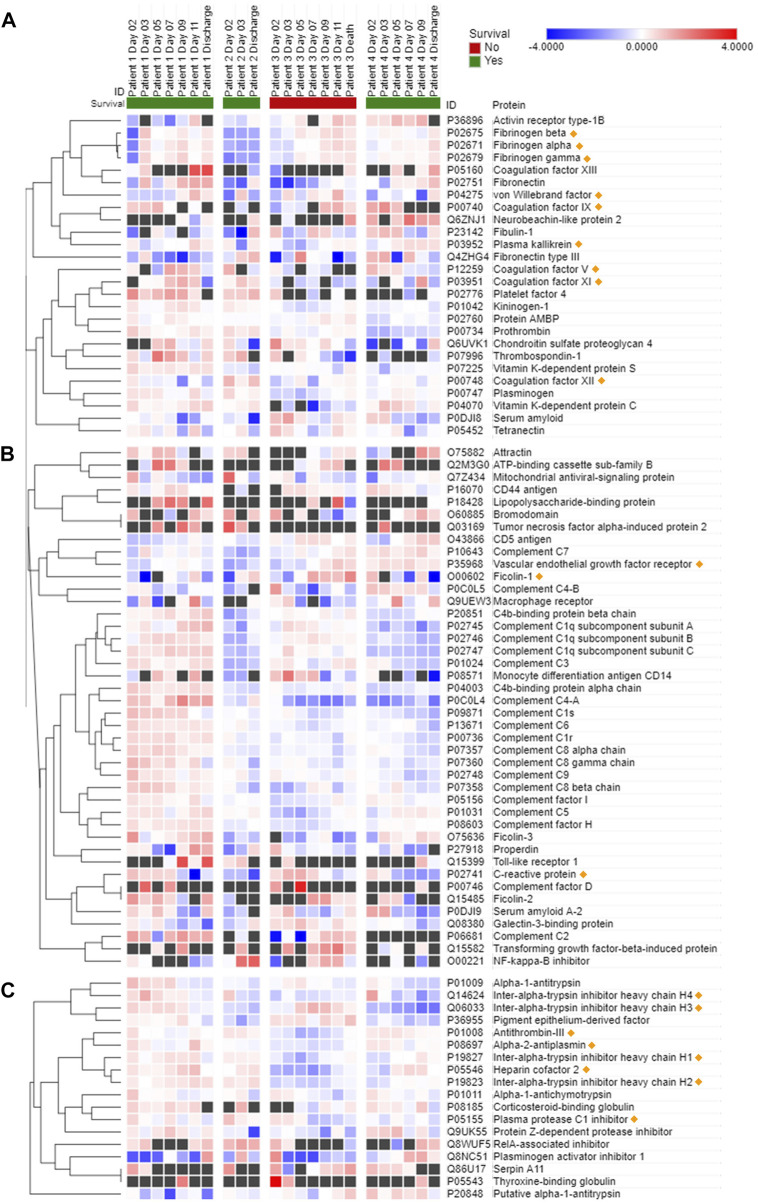
Most significant proteins involved in hemostasis **(A)**, immune or inflammatory response **(B)**, and endogenous protease inhibition **(C)** detected in COVID-19 patient plasma. Data are presented as Log2 ratio between the value at a specific time point and the average of the intensity values at all time points. Black squares indicate that a protein was not detected in the sample. Rows of identified proteins were hierarchically clustered with the one minus Pearson’s correlation metric and average linkage method for each protein group. Columns were sorted by patient and ordered by day of sample collection. Morpheus (https://software.broadinstitute.org/morpheus/) was used to generate the heatmaps.

Protease activity data are reported in Figure 1 (hemostasis-mediating proteases) and in Figure 2 (trypsin-like enzymes and aminopeptidase). Figure 1 shows that plasmin activity remained stable over time and between patients, serving as internal control of enzymatic activity. Thrombin and Factor IX activity showed an increasing trend over the first 5 days of ICU admission followed by gradual decrease. Factor X activity decreased over time in Patients 1 and 2 compared to day 2 and increased in Patients 3 and 4. The main finding was the marked increase in Factor VII activity and tryptic-like activity, particularly at a terminal Val-Arg (Figure 2) in Patient 3 on day 7, when bacterial superinfection was diagnosed. Figure 2 also shows static methionine aminopeptidase (MetAP) activity (Vivitide®, Gardner, MA), serving as a *de facto* negative control for the assay. The intensity of the proteins of interest, derived from proteomic analysis, are reported in the heat maps in Figure 3. Proteins characterized by the most evident trends (over time and between patients) are highlighted with a diamond sign next to the protein name. For the complete proteome profile, see Supplementary File S2.

Patient 3 exhibited a reduction in several plasma coagulation proteins such as coagulation factors V and XI on day 7 and thereafter, and a concomitant increase in the expression of fibrinogen, plasma kallikrein and von Willebrand factor (Figure 3A). Overall, there were mild changes over time in the abundance of proteins involved in the immune and inflammatory response of Patient 3, as shown by diverging trends of increase and decrease of the complement factors (Figure 3B). Interestingly, starting on day 7 ficolin-1 and VEGF 2 were increased in Patient 3, while the trend was less apparent and characterized by some variability in the other patients. C-reactive protein showed a progressively decreasing trend in expression over time in all four patients. A general trend towards a reduction in the expression of protease inhibitors, in particular of endogenous trypsin inhibitors, was observed in Patient 3 (Figure 3C). Thrombin inhibitors, such as antithrombin-III and heparin cofactor II, were also downregulated in Patient 3 compared to the survivors at all time points.

**FIGURE 4:**
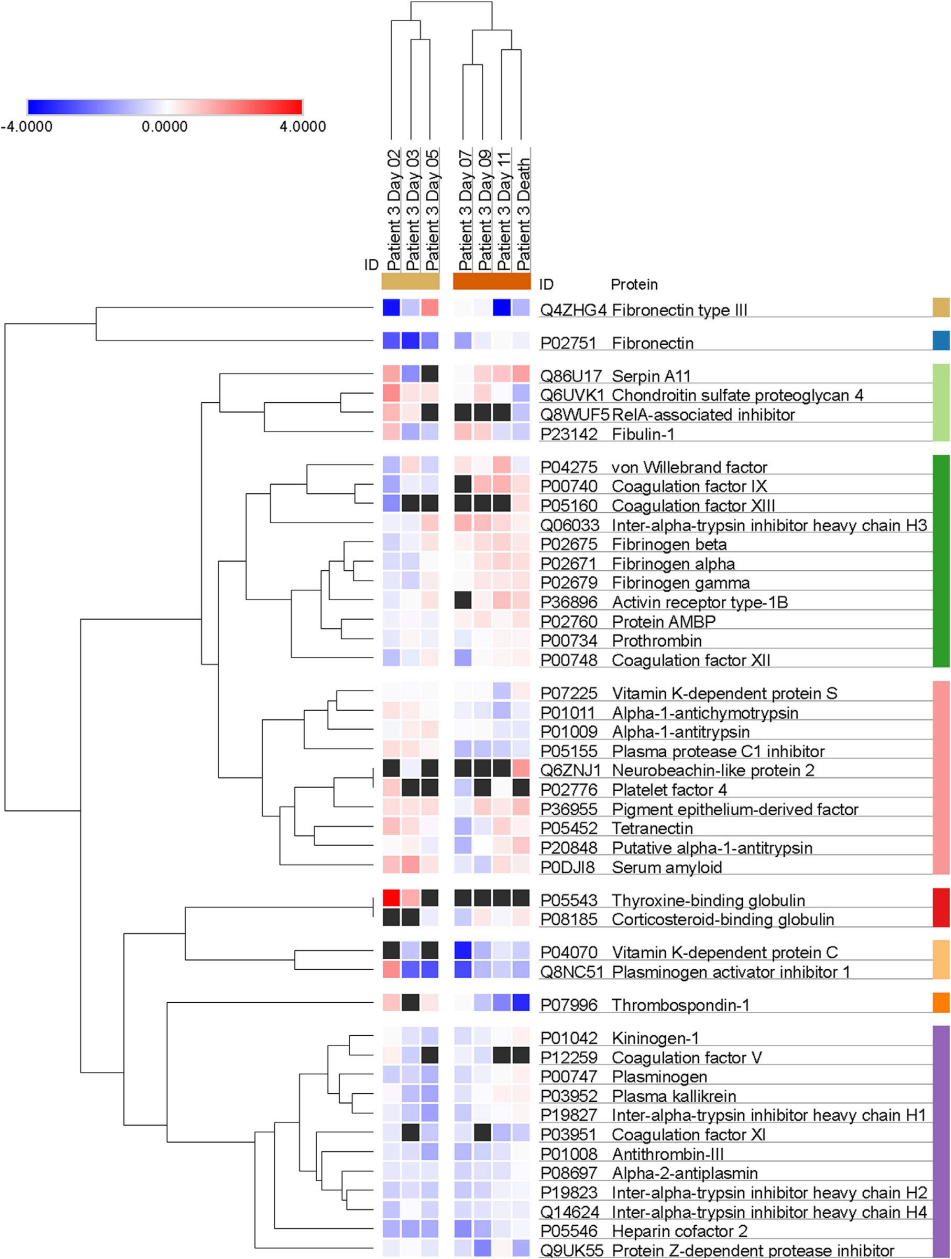
Trends of clustering between proteins involved in hemostasis regulation and endogenous protease inhibition across multiple days for Patient 3. Data are presented as Log2 ratio between the value at a specific time point and the average of the intensity values at all time points. Black squares indicate that a protein was not detected in the sample. Rows of identified proteins were hierarchically clustered with the one minus Pearson’s correlation metric and average linkage method for each protein group.

Additional clustering analyses focused specifically on Patient 3 (Figure 4) and revealed a trend of apparent correlation between the expression of several proteins (involved either in hemostasis regulation or in endogenous enzymatic inhibition) and the advancement of the disease after day 7 as opposed to the trends before day 7.

**FIGURE 5:**
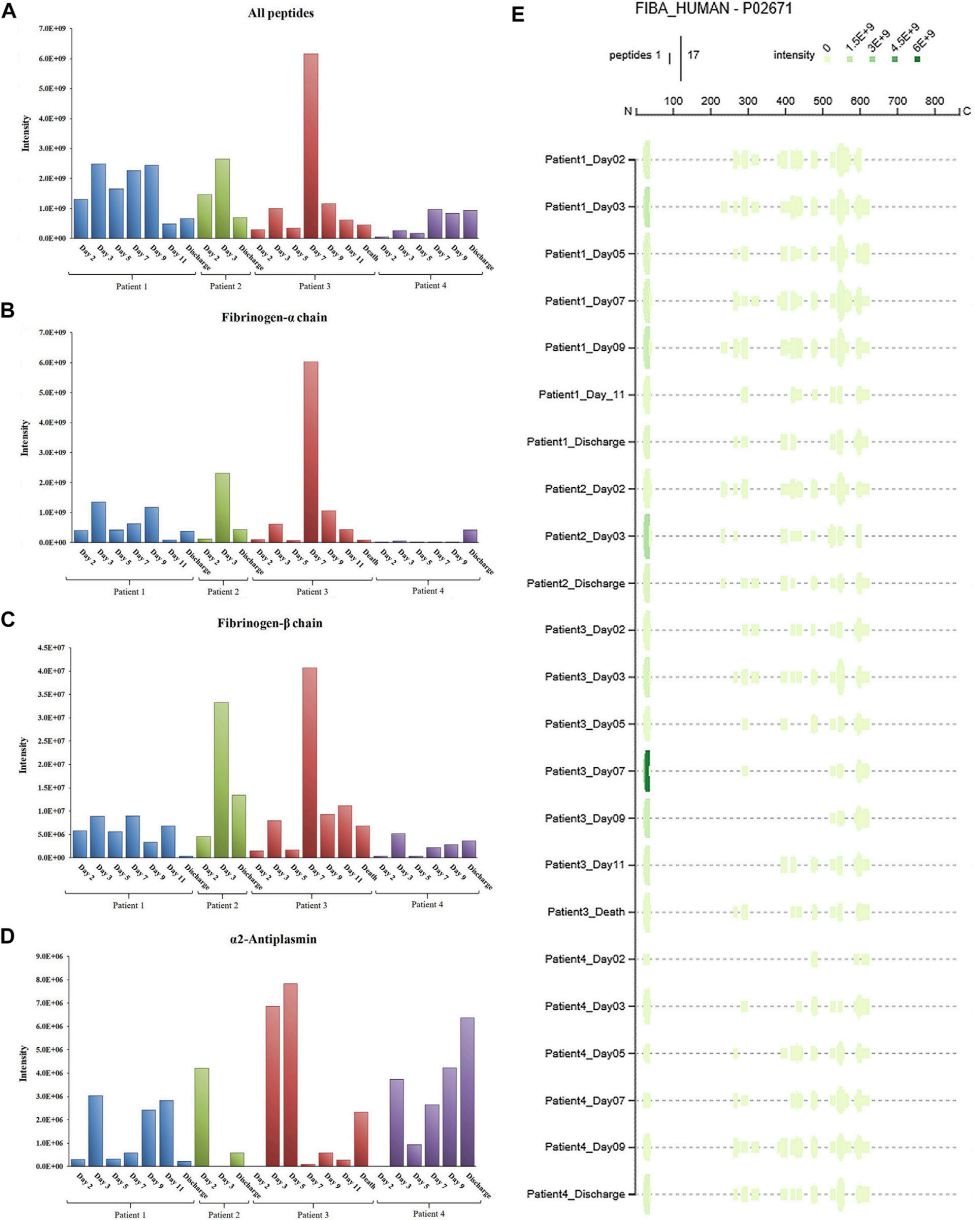
Total intensity as the sum of the intensities of the peptides detected in COVID-19 patient plasma: a) from the whole proteome **(A)**; b) from fibrinogen-α **(B)**; c) from fibrinogen-β **(C)**; d) from alpha-2-antiplasmin **(D)**. Peptigram representation of the cleavage pattern of fibrinogen-α in the four patients at each time point **(E)**.

Figure 5 summarizes the main results of the peptidomic analysis, considering the specific aims of our study. The sum of the intensities of all the peptide fragments detected at each time point from a total of 233 source proteins suggests marked proteolytic activity in Patient 3 on day 7 (Figure 5A). This ongoing protein degradation was particularly evident in fibrinogen-α and fibrinogen-β (Figures 5B, C). Of note, peptides derived from α2-antiplasmin (Figure 5D) had a characteristic trend in Patients 1, 3 and 4, with a progressive increase until day 5, followed by an abrupt reduction and a progressive increase thereafter. Figure 5E shows the unique patterns of protein cleavage for fibrinogen-α obtained by Peptigram analysis and the size and location of the fragments that suggest that the enhanced proteolytic activity seen on day 7 in Patient 3 increases the amount of circulating fibrinopeptide A (FPA).

The link to the complete peptidome profile as analyzed *via* Peptigram can be found at: http://bioware.ucd.ie/peptigram/job/7jbnkglrh290.

## 4 Discussion

Omics approaches (metabolomics, proteomics, peptidomics, transcriptomics, genomics) to study disease, including acute illness, have become increasingly popular due to the unique multiscale insights they provide that can result in better targeted treatments (Aletti et al., 2016a; Cambiaghi et al., 2017; Sharma et al., 2017; Bauzá-Martinez et al., 2018; Cambiaghi et al., 2018; Braga et al., 2019; Mangioni et al., 2020; Schuurman et al., 2021). Several contributions have already been made to the field of COVID-19 by proteomics (Messner et al., 2020; Demichev et al., 2021; Haas et al., 2021; McArdle et al., 2021).

We have previously leveraged -omics analyses to test and validate the hypothesis that dysregulated systemic proteolysis occurs in circulatory shock and degrades several proteins with an important role in the maintenance of physiologic homeostasis. To this end, we introduced a peptidomic-based assay, which estimates the magnitude of *in vivo* proteolysis by assessing the abundance and count of peptides in a plasma sample (Aletti et al., 2016b; Bauzá-Martinez et al., 2018). Analysis of the circulating peptidome by this method showed an association between enhanced proteolysis and mortality (Bauzá-Martinez et al., 2018) in patients with septic shock caused by bacterial infection. Therefore, we investigated whether a similar association may also be found in viral sepsis caused by COVID-19 infection with bacterial superinfection developing over the course of the ICU stay. In addition to our previously developed mass spectrometry-based peptidomic assay, we also quantified the proteolytic activity of proteases that are known to be activated in acute illness, such as enzymes responsible for hemostasis regulation (clotting factors and plasmin), and other serine proteases characterized by tryptic-like activity. The combination of peptidomic (and proteomic) data and enzymatic activity measurements during the course of patients’ disease process aimed to shed light on systemic intravascular proteolysis, which may play a pivotal role in the pathogenesis of potentially lethal coagulopathies seen in acute illness, given the crucial role of proteases in the regulation of the balance between coagulation and fibrinolysis.

Protease activity patterns showed a marked increase of Factor VII and Factor IX activity on day 7 in the patient who died of superinfection. In particular, the spike in Factor VII activity was unique amongst our sample cohort and represents an 80-fold increase relative to that found in healthy control plasma. Further, the same trend in activity was also found in two substrates identified by the cleavage sites Val-Arg and Leu-Arg, representative of tryptic-like proteolytic activity. Although the activity of all four substrates (Factor VII, Factor IX, Val-Arg, Leu-Arg) tended to decrease after the initial increase, the peak on day 7 in Patient 3 coincided with the diagnosis of bacterial superinfection and with an increase in peptide abundance, particularly from fibrinogen (as detailed in Figure 5). Most fibrinogen-derived peptides in Patient 3 on day 7 originated from the Fibrinopeptide A (FPA) domain, located at the N-terminal region of the fibrinogen Aα chain (Aα 20–35), which is normally generated by thrombin cleavage of fibrinogen upon activation of the clotting cascade. FPA is a known biomarker of the activation of the coagulation system and was shown to be related to elevated inflammation in sepsis, including COVID-19-induced sepsis (Ranucci et al., 2020). Although thrombin was not found to be overactivated in our patient sample, other serine proteases may contribute to fibrinogen cleavage at typical thrombin cleaving sites.

Further, our proteomic assay detected reduced levels of antithrombin-III, heparin cofactor II and, perhaps most importantly, Protein C in Patient 3 compared to the three surviving patients. These findings, especially the minimum of Protein C concentration measured on day 7, also hint at an impairment of hemostasis in this patient. The limited availability of one of the major circulating anticoagulant factors, such as Protein C, and of inhibitors responsible for thrombin regulation, such as antithrombin-III, regardless of thrombin expression and activity levels, and of etiology (pathological proteolysis, increased protein use, decreased expression, etc.) is suggestive of a possible imbalance between procoagulant and anticoagulant factors, with a potential impact on the risk for either thrombosis or bleeding. Future studies on the impairment induced by shock on hemostasis should address the dynamic changes in this balance, in order to further explore the possible associations between infection, enhanced proteolysis, and dysregulation of the clotting cascade and hemostasis in general. Non-etheless, while we cannot draw any conclusion regarding the possible trends of correlation between the expression of proteins or groups of proteins that could be affected by the progression of the disease due to the limitation of the clustering analysis that was applied to the single patient who didn't survive (as shown in Figure 4 for Patient 3), it is interesting to observe that there may be patterns of concomitant dysregulation in specific clotting factors and enzyme inhibitors with possible pathophysiological and clinical implications.

Consistent with our prior findings of enhanced proteolysis over time in patients who died of septic shock (Bauzá-Martinez et al., 2018), these results may indicate a possible relationship between a surge in proteolytic activity in concert with impairment of the coagulation cascade and fibrinolysis and worsening overall clinical course after development of bacterial infection following the initial progression of COVID-19 infection. This was clinically apparent in Patient 3 who had been on either phenylephrine or norepinephrine (but not both) for hypotension from admission to the ICU, and on day 7 necessitated the addition of two simultaneous pressor agents (norepinephrine and vasopressin) to maintain adequate hemodynamics. This pressor requirement subsequently increased the following day to three simultaneous pressor agents (norepinephrine, phenylephrine, and vasopressin) before later subsiding.

As COVID-19 infection has a strong association with coagulopathy (Asakura and Ogawa, 2020; Iba et al., 2020), we focused on the activity of enzymes involved in the clotting cascade and fibrinolysis regulation and other trypsin-like proteases, which could be characterized by a similar array of target proteins, including but not limited to coagulation enzymes and fibrinogen. Thromboembolism and reports of microvascular thrombi akin to disseminated intravascular coagulation (DIC) have been a common reported cause of death in patients admitted to the ICU with severe COVID-19. Although the cause of death in Patient 3 was classified as septic shock, the possible association between enhanced proteolytic activity, bacterial superinfection and disease severity is non-etheless of interest and could point to i) the presence of additional pathological mechanisms (i.e., proteolysis) that contribute to poor outcomes, and ii) the relationship between enzymatic activity patterns and bacterial superinfection that causes further illness, in addition to the original viral infection.

Our approach could also help disentangle the role of specific enzymes in the overall enhancement of systemwide proteolysis. In particular, we generated data potentially useful to better understand the pathophysiological alterations of biological systems regulated by proteases with competing functions. The observation that the activity of specific clotting factors was enhanced, while plasmin activity levels remained unaltered, suggests that the dynamic equilibrium between coagulation and fibrinolysis could be disrupted in acute illness. In turn, this could lead to hemostasis impairment and coagulopathies. In the absence of accurate measurements to predict hemostasis disorders, the combined use of quantitative enzymatic activity assay and mass spectrometry analysis of the circulating proteome and peptidome warrants further research to advance the understanding of the possible imbalance between enzymes with competing functions, as well as between proteases and their endogenous inhibitors.

The association between disease severity and -omics patterns is also supported by the measurements of other proteins that play a role in immunity and inflammation. For instance, the trends in the expression of ficolin-1 and VEGFR2, and to some extent C-reactive protein (which showed a decrease over time in all four patients but was much milder in Patient 3) correlate with the exacerbation of the inflammatory state of Patient 3 following diagnosis of bacterial superinfection. Ficolin-1 is a known activator of the complement system and innate immunity through recognition of pathogen-associated molecular patterns (PAMPs) (Bidula et al., 2019; Polycarpou et al., 2020), while VEGFR2 has been related to COVID-19 progression (Kong et al., 2020; Birnhuber et al., 2021). In fact, the patient with the least severe course of disease (Patient 2) had the lowest VEGFR2 concentration and, importantly, was the only patient who wasn't diagnosed with ARDS. Patient 4, on the other hand, had rheumatoid arthritis and subsequently greater VEGFR2 intensity. These observations highlight that the complex interplay between baseline comorbidities, disease severity and variability of immune and inflammatory responses prevents from interpreting conclusively the expression trends of complement factors and warrants further research in the future.

There are some limitations of this study that need to be acknowledged. First, the low sample size of the studied patient population and the non-uniform time distribution of the collected samples and relevant data hinders the ability to make stronger inferences than are made here; the robustness of the presented results would doubtlessly have been strengthened with a larger sample size and a more homogeneous database, especially considering the impact on the available -omics data. Therefore, no firm generalizations can be made about multiomics and proteolytic changes in COVID-19 patients based on the reported results. Second, the four participants in our study were characterized by significant inter-subject variability and heterogeneity, as shown for example by age distribution, duration of ICU stay and hospitalization and of days on/off mechanical ventilation, combination of antibiotic and vasopressor therapy, severity, incomplete coagulation panel measures, etc. While it is possible that age was a strong determinant of Patient 3’s outcome, the presence of bacterial superinfection is strongly suspected to have been responsible for the unique results that our approach generated. Third, the inclusion of a patient subgroup with acute illness other than COVID-19, for instance bacterial sepsis and septic shock, would have added an important control sample. Unfortunately, the emergency logistics of the ICUs during the first waves of the pandemic made it impossible to have access to other types of patients, and convenience sampling was the only realistic option for patient recruitment. Fourth, regarding data drop-out, this was a real-world observational study, and the clinical laboratory measurements and values reflect patient management of severely ill COVID-19 patients in the ICU. Fifth, the mass spectrometry approach was necessarily untargeted; if specific protein systems and products of their degradation were targeted, it is possible that potential alterations in these systems could be highlighted in greater detail. Finally, when comparing and integrating proteomics and peptidomics data, the availability of transcriptomics data, which weren't part of the experimental design of this observational study, could add information on protein regulation, and help understand to a greater depth the impact of abnormal proteolysis and protein synthesis, breakdown, and turnover.

In summary, we show here that the analysis of the circulating proteome and peptidome, coupled with quantitation of proteolytic activity in plasma of COVID-19 patients in time, is a powerful methodological tool that can provide pathophysiologically and clinically meaningful data. Our approach may prove particularly useful to more thoroughly describe how the activation and regulation of hemostasis, a complex protease-mediated system which plays a critical role in the management of acutely ill patients in the ICU, is impaired in septic shock. The association between proteolytic activity and protease expression patterns achieved by mass spectrometry investigation of plasma and bacterial superinfection in COVID-19 may also contribute to anticipate the signs of possible coagulation disorders and aid in the early diagnosis and delivery of a timely therapy. However, considering the limitations related to the low sample size of our study, its main contribution is represented by the proof-of-concept of the usefulness of the proposed experimental and analytical workflow. Despite the small number of subjects enrolled in the study, we tested as a rigorous approach as possible to quantitatively measure several important, but clinically neglected parameters of protease-mediated processes that may be fatally dysregulated in septic shock. Future research will have to thoroughly address the limitations of this report, with the goal of validating our preliminary observations about the proteolytic activity and degradation patterns of proteins involved in hemostasis regulation. This will be made possible by the application of the complex, multidata methodological approach utilized in this study to larger and more homogenous patient groups, characterized by comparable clinical representations and including different degrees of severity and specific manifestations of hemostasis disorders.

## Data Availability

The datasets presented in this study can be found in online repositories. The names of the repository and accession number can be found below: ProteomeXchange (http://proteomecentral.proteomexchange.org/cgi/GetDataset), PXD029181.
