# Supplementary material for: Plasma enzymatic activity, proteomics and peptidomics in COVID-19-induced sepsis: A novel approach for the analysis of hemostasis
Source: Front Mol Biosci. 2023 Jan 11;9:1051471. doi: 10.3389/fmolb.2022.1051471 (PMC9874325; doi:10.3389/fmolb.2022.1051471)
Supplement: Supplementary file 1 [file DataSheet4.PDF]

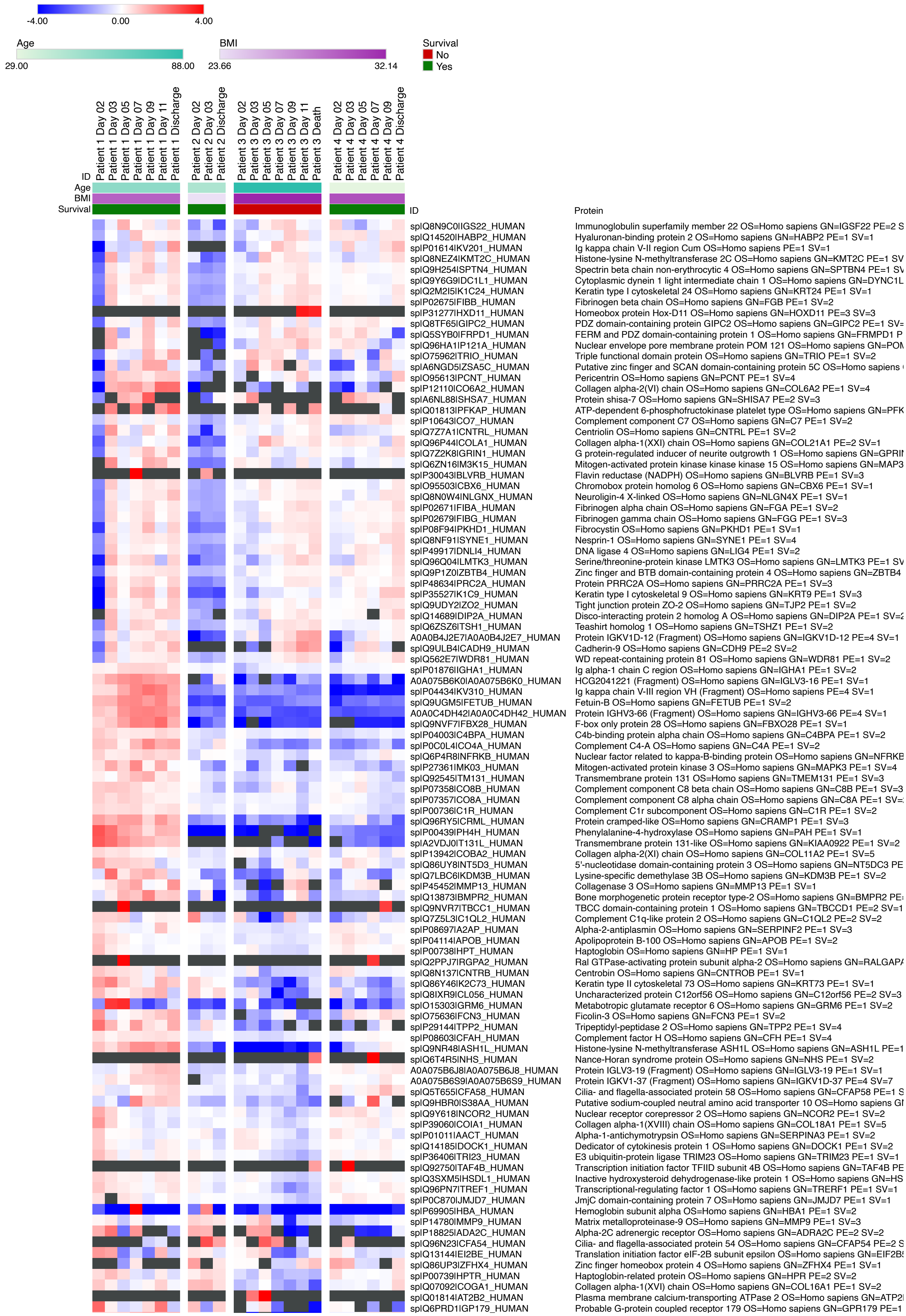
-4.00
0.00
4.00
Age
29.00
88.00
BMI
23.66
32.14
Survival
No
Yes
Patient 1 Day 02
Patient 1 Day 03
Patient 1 Day 05
Patient 1 Day 07
Patient 1 Day 09
Patient 1 Day 11
Patient 1 Discharge
Patient 2 Day 02
Patient 2 Day 03
Patient 2 Discharge
Patient 3 Day 02
Patient 3 Day 03
Patient 3 Day 05
Patient 3 Day 07
Patient 3 Day 09
Patient 3 Day 11
Patient 3 Death
Patient 4 Day 02
Patient 4 Day 03
Patient 4 Day 05
Patient 4 Day 07
Patient 4 Day 09
Patient 4 Discharge
ID
Age
BMI
Survival
ID
Protein
sp|Q8N9C0|IGS22_HUMAN Immunoglobulin superfamily member 22 OS=Homo sapiens GN=IGSF22 PE=2 S
sp|Q14520|HABP2_HUMAN Hyaluronan-binding protein 2 OS=Homo sapiens GN=HABP2 PE=1 SV=1
sp|P01614|KV201_HUMAN Ig kappa chain V-II region Cum OS=Homo sapiens PE=1 SV=1
sp|Q8NEZ4|KMT2C_HUMAN Histone-lysine N-methyltransferase 2C OS=Homo sapiens GN=KMT2C PE=1 SV
sp|Q9H254|SPTN4_HUMAN Spectrin beta chain non-erythrocytic 4 OS=Homo sapiens GN=SPTBN4 PE=1 SV
sp|Q9Y6G9|DC1L1_HUMAN Cytoplasmic dynein 1 light intermediate chain 1 OS=Homo sapiens GN=DYNC1L
sp|Q2M2I5|K1C24_HUMAN Keratin type I cytoskeletal 24 OS=Homo sapiens GN=KRT24 PE=1 SV=1
sp|P02675|FIBB_HUMAN Fibrinogen beta chain OS=Homo sapiens GN=FGB PE=1 SV=2
sp|P31277|HXD11_HUMAN Homeobox protein Hox-D11 OS=Homo sapiens GN=HOXD11 PE=3 SV=3
sp|Q8TF65|GIPC2_HUMAN PDZ domain-containing protein GIPC2 OS=Homo sapiens GN=GIPC2 PE=1 SV=
sp|Q5SYB0|FRPD1_HUMAN FERM and PDZ domain-containing protein 1 OS=Homo sapiens GN=FRMPD1 P
sp|Q96HA1|P121A_HUMAN Nuclear envelope pore membrane protein POM 121 OS=Homo sapiens GN=POM
sp|O75962|TRIO_HUMAN Triple functional domain protein OS=Homo sapiens GN=TRIO PE=1 SV=2
sp|A6NGD5|ZSA5C_HUMAN Putative zinc finger and SCAN domain-containing protein 5C OS=Homo sapiens
sp|O95613|PCNT_HUMAN Pericentrin OS=Homo sapiens GN=PCNT PE=1 SV=4
sp|P12110|CO6A2_HUMAN Collagen alpha-2(VI) chain OS=Homo sapiens GN=COL6A2 PE=1 SV=4
sp|A6NL88|SHSA7_HUMAN Protein shisa-7 OS=Homo sapiens GN=SHISA7 PE=2 SV=3
sp|Q01813|PFKAP_HUMAN ATP-dependent 6-phosphofructokinase platelet type OS=Homo sapiens GN=PFK
sp|P10643|CO7_HUMAN Complement component C7 OS=Homo sapiens GN=C7 PE=1 SV=2
sp|Q7Z7A1|CNTRL_HUMAN Centriolin OS=Homo sapiens GN=CNTRL PE=1 SV=2
sp|Q96P44|COLA1_HUMAN Collagen alpha-1(XXI) chain OS=Homo sapiens GN=COL21A1 PE=2 SV=1
sp|Q7Z2K8|GRIN1_HUMAN G protein-regulated inducer of neurite outgrowth 1 OS=Homo sapiens GN=GPRIN
sp|Q6ZN16|M3K15_HUMAN Mitogen-activated protein kinase kinase kinase 15 OS=Homo sapiens GN=MAP3
sp|P30043|BLVRB_HUMAN Flavin reductase (NADPH) OS=Homo sapiens GN=BLVRB PE=1 SV=3
sp|O95503|CBX6_HUMAN Chromobox protein homolog 6 OS=Homo sapiens GN=CBX6 PE=1 SV=1
sp|Q8N0W4|NLGNX_HUMAN Neuroligin-4 X-linked OS=Homo sapiens GN=NLGN4X PE=1 SV=1
sp|P02671|FIBA_HUMAN Fibrinogen alpha chain OS=Homo sapiens GN=FGA PE=1 SV=2
sp|P02679|FIBG_HUMAN Fibrinogen gamma chain OS=Homo sapiens GN=FGG PE=1 SV=3
sp|P08F94|PKHD1_HUMAN Fibrocystin OS=Homo sapiens GN=PKHD1 PE=1 SV=1
sp|Q8NF91|SYNE1_HUMAN Nesprin-1 OS=Homo sapiens GN=SYNE1 PE=1 SV=4
sp|P49917|DNLI4_HUMAN DNA ligase 4 OS=Homo sapiens GN=LIG4 PE=1 SV=2
sp|Q96Q04|LMTK3_HUMAN Serine/threonine-protein kinase LMTK3 OS=Homo sapiens GN=LMTK3 PE=1 SV
sp|Q9P1Z0|ZBTB4_HUMAN Zinc finger and BTB domain-containing protein 4 OS=Homo sapiens GN=ZBTB4
sp|P48634|PRC2A_HUMAN Protein PRRC2A OS=Homo sapiens GN=PRRC2A PE=1 SV=3
sp|P35527|K1C9_HUMAN Keratin type I cytoskeletal 9 OS=Homo sapiens GN=KRT9 PE=1 SV=3
sp|Q9UDY2|ZO2_HUMAN Tight junction protein ZO-2 OS=Homo sapiens GN=TJP2 PE=1 SV=2
sp|Q14689|DIP2A_HUMAN Disco-interacting protein 2 homolog A OS=Homo sapiens GN=DIP2A PE=1 SV=2
sp|Q6ZSZ6|TSH1_HUMAN Teashirt homolog 1 OS=Homo sapiens GN=TSHZ1 PE=1 SV=2
A0A0B4J2E7|A0A0B4J2E7_HUMAN Protein IGKV1D-12 (Fragment) OS=Homo sapiens GN=IGKV1D-12 PE=4 SV=1
sp|Q9ULB4|CADH9_HUMAN Cadherin-9 OS=Homo sapiens GN=CDH9 PE=2 SV=2
sp|Q562E7|WDR81_HUMAN WD repeat-containing protein 81 OS=Homo sapiens GN=WDR81 PE=1 SV=2
sp|P01876|IGHA1_HUMAN Ig alpha-1 chain C region OS=Homo sapiens GN=IGHA1 PE=1 SV=2
A0A075B6K0|A0A075B6K0_HUMAN HCG2041221 (Fragment) OS=Homo sapiens GN=IGLV3-16 PE=1 SV=1
sp|P04434|KV310_HUMAN Ig kappa chain V-III region VH (Fragment) OS=Homo sapiens PE=4 SV=1
sp|Q9UGM5|FETUB_HUMAN Fetuin-B OS=Homo sapiens GN=FETUB PE=1 SV=2
A0A0C4DH42|A0A0C4DH42_HUMAN Protein IGHV3-66 (Fragment) OS=Homo sapiens GN=IGHV3-66 PE=4 SV=1
sp|Q9NVF7|FBX28_HUMAN F-box only protein 28 OS=Homo sapiens GN=FBXO28 PE=1 SV=1
sp|P04003|C4BPA_HUMAN C4b-binding protein alpha chain OS=Homo sapiens GN=C4BPA PE=1 SV=2
sp|P0C0L4|CO4A_HUMAN Complement C4-A OS=Homo sapiens GN=C4A PE=1 SV=2
sp|Q6P4R8|NFRKB_HUMAN Nuclear factor related to kappa-B-binding protein OS=Homo sapiens GN=NFRKB
sp|P27361|MK03_HUMAN Mitogen-activated protein kinase 3 OS=Homo sapiens GN=MAPK3 PE=1 SV=4
sp|Q92545|TM131_HUMAN Transmembrane protein 131 OS=Homo sapiens GN=TMEM131 PE=1 SV=3
sp|P07358|CO8B_HUMAN Complement component C8 beta chain OS=Homo sapiens GN=C8B PE=1 SV=3
sp|P07357|CO8A_HUMAN Complement component C8 alpha chain OS=Homo sapiens GN=C8A PE=1 SV=
sp|P00736|C1R_HUMAN Complement C1r subcomponent OS=Homo sapiens GN=C1R PE=1 SV=2
sp|Q96RY5|CRML_HUMAN Protein cramped-like OS=Homo sapiens GN=CRAMP1 PE=1 SV=3
sp|P00439|PH4H_HUMAN Phenylalanine-4-hydroxylase OS=Homo sapiens GN=PAH PE=1 SV=1
sp|A2VDJ0|T131L_HUMAN Transmembrane protein 131-like OS=Homo sapiens GN=KIAA0922 PE=1 SV=2
sp|P13942|COBA2_HUMAN Collagen alpha-2(XI) chain OS=Homo sapiens GN=COL11A2 PE=1 SV=5
sp|Q86UY8|NT5D3_HUMAN 5'-nucleotidase domain-containing protein 3 OS=Homo sapiens GN=NT5DC3 PE
sp|Q7LBC6|KDM3B_HUMAN Lysine-specific demethylase 3B OS=Homo sapiens GN=KDM3B PE=1 SV=2
sp|P45452|MMP13_HUMAN Collagenase 3 OS=Homo sapiens GN=MMP13 PE=1 SV=1
sp|Q13873|BMPR2_HUMAN Bone morphogenetic protein receptor type-2 OS=Homo sapiens GN=BMPR2 PE=
sp|Q9NVR7|TBCC1_HUMAN TBCC domain-containing protein 1 OS=Homo sapiens GN=TBCCD1 PE=2 SV=1
sp|Q7Z5L3|C1QL2_HUMAN Complement C1q-like protein 2 OS=Homo sapiens GN=C1QL2 PE=2 SV=2
sp|P08697|A2AP_HUMAN Alpha-2-antiplasmin OS=Homo sapiens GN=SERPINF2 PE=1 SV=3
sp|P04114|APOB_HUMAN Apolipoprotein B-100 OS=Homo sapiens GN=APOB PE=1 SV=2
sp|P00738|HPT_HUMAN Haptoglobin OS=Homo sapiens GN=HP PE=1 SV=1
sp|Q2PPJ7|RGPA2_HUMAN Ral GTPase-activating protein subunit alpha-2 OS=Homo sapiens GN=RALGAPA
sp|Q8N137|CNTRB_HUMAN Centrobin OS=Homo sapiens GN=CNTROB PE=1 SV=1
sp|Q86Y46|K2C73_HUMAN Keratin type II cytoskeletal 73 OS=Homo sapiens GN=KRT73 PE=1 SV=1
sp|Q8IXR9|CL056_HUMAN Uncharacterized protein C12orf56 OS=Homo sapiens GN=C12orf56 PE=2 SV=3
sp|O15303|GRM6_HUMAN Metabotropic glutamate receptor 6 OS=Homo sapiens GN=GRM6 PE=1 SV=2
sp|O75636|FCN3_HUMAN Ficolin-3 OS=Homo sapiens GN=FCN3 PE=1 SV=2
sp|P29144|TPP2_HUMAN Tripeptidyl-peptidase 2 OS=Homo sapiens GN=TPP2 PE=1 SV=4
sp|P08603|CFAH_HUMAN Complement factor H OS=Homo sapiens GN=CFH PE=1 SV=4
sp|Q9NR48|ASH1L_HUMAN Histone-lysine N-methyltransferase ASH1L OS=Homo sapiens GN=ASH1L PE=1
sp|Q6T4R5|NHS_HUMAN Nance-Horan syndrome protein OS=Homo sapiens GN=NHS PE=1 SV=2
A0A075B6J8|A0A075B6J8_HUMAN Protein IGLV3-19 (Fragment) OS=Homo sapiens GN=IGLV3-19 PE=1 SV=1
A0A075B6S9|A0A075B6S9_HUMAN Protein IGKV1-37 (Fragment) OS=Homo sapiens GN=IGKV1D-37 PE=4 SV=7
sp|Q5T655|CFA58_HUMAN Cilia- and flagella-associated protein 58 OS=Homo sapiens GN=CFAP58 PE=1 S
sp|Q9HBR0|S38AA_HUMAN Putative sodium-coupled neutral amino acid transporter 10 OS=Homo sapiens GN
sp|Q9Y618|NCOR2_HUMAN Nuclear receptor corepressor 2 OS=Homo sapiens GN=NCOR2 PE=1 SV=2
sp|P39060|COIA1_HUMAN Collagen alpha-1(XVIII) chain OS=Homo sapiens GN=COL18A1 PE=1 SV=5
sp|P01011|AACT_HUMAN Alpha-1-antichymotrypsin OS=Homo sapiens GN=SERPINA3 PE=1 SV=2
sp|Q14185|DOCK1_HUMAN Dedicator of cytokinesis protein 1 OS=Homo sapiens GN=DOCK1 PE=1 SV=2
sp|P36406|TRI23_HUMAN E3 ubiquitin-protein ligase TRIM23 OS=Homo sapiens GN=TRIM23 PE=1 SV=1
sp|Q92750|TAF4B_HUMAN Transcription initiation factor TFIID subunit 4B OS=Homo sapiens GN=TAF4B PE
sp|Q3SXM5|HSDL1_HUMAN Inactive hydroxysteroid dehydrogenase-like protein 1 OS=Homo sapiens GN=HS
sp|Q96PN7|TREF1_HUMAN Transcriptional-regulating factor 1 OS=Homo sapiens GN=TRERF1 PE=1 SV=1
sp|P0C870|JMJD7_HUMAN JmjC domain-containing protein 7 OS=Homo sapiens GN=JMJD7 PE=1 SV=1
sp|P69905|HBA_HUMAN Hemoglobin subunit alpha OS=Homo sapiens GN=HBA1 PE=1 SV=2
sp|P14780|MMP9_HUMAN Matrix metalloproteinase-9 OS=Homo sapiens GN=MMP9 PE=1 SV=3
sp|P18825|ADA2C_HUMAN Alpha-2C adrenergic receptor OS=Homo sapiens GN=ADRA2C PE=2 SV=2
sp|Q96N23|CFA54_HUMAN Cilia- and flagella-associated protein 54 OS=Homo sapiens GN=CFAP54 PE=2 S
sp|Q13144|EI2BE_HUMAN Translation initiation factor eIF-2B subunit epsilon OS=Homo sapiens GN=EIF2B
sp|Q86UP3|ZFHX4_HUMAN Zinc finger homeobox protein 4 OS=Homo sapiens GN=ZFHX4 PE=1 SV=1
sp|P00739|HPTR_HUMAN Haptoglobin-related protein OS=Homo sapiens GN=HPR PE=2 SV=2
sp|Q07092|COGA1_HUMAN Collagen alpha-1(XVI) chain OS=Homo sapiens GN=COL16A1 PE=1 SV=2
sp|Q01814|AT2B2_HUMAN Plasma membrane calcium-transporting ATPase 2 OS=Homo sapiens GN=ATP2
sp|Q6PRD1|GP179_HUMAN Probable G-protein coupled receptor 179 OS=Homo sapiens GN=GPR179 PE=1

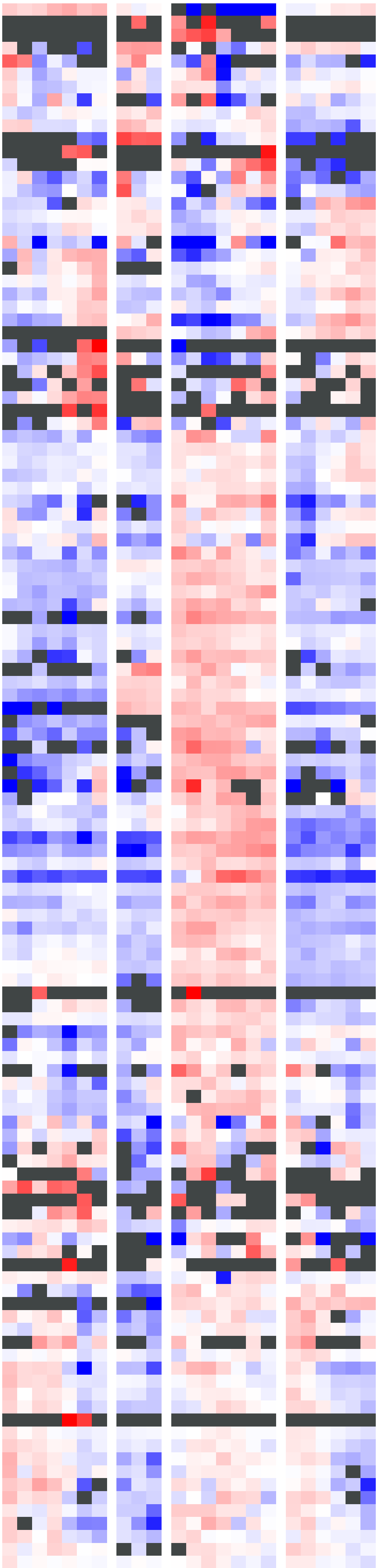

A0A075B6I9|A0A075B6I9_HUMAN	Protein IGLV7-46 (Fragment) OS=Homo sapiens GN=IGLV7-46 PE=1 SV=4
sp|P55196|AFAD_HUMAN	Afadin OS=Homo sapiens GN=MLLT4 PE=1 SV=3
sp|P29353|SHC1_HUMAN	SHC-transforming protein 1 OS=Homo sapiens GN=SHC1 PE=1 SV=4
A0A0B4J2B5|A0A0B4J2B5_HUMAN	Protein IGHV3OR16-9 (Fragment) OS=Homo sapiens GN=IGHV3OR16-9 PE=1
sp|Q5VY09|IER5_HUMAN	Immediate early response gene 5 protein OS=Homo sapiens GN=IER5 PE=1 SV
sp|O95359|TACC2_HUMAN	Transforming acidic coiled-coil-containing protein 2 OS=Homo sapiens GN=TACC
sp|Q12873|CHD3_HUMAN	Chromodomain-helicase-DNA-binding protein 3 OS=Homo sapiens GN=CHD3 P
sp|P59665|DEF1_HUMAN	Neutrophil defensin 1 OS=Homo sapiens GN=DEFA1 PE=1 SV=1
sp|P02765|FETUA_HUMAN	Alpha-2-HS-glycoprotein OS=Homo sapiens GN=AHSG PE=1 SV=1
sp|P01861|IGHG4_HUMAN	Ig gamma-4 chain C region OS=Homo sapiens GN=IGHG4 PE=1 SV=1
sp|P0CF74|LAC6_HUMAN	Ig lambda-6 chain C region OS=Homo sapiens GN=IGLC6 PE=4 SV=1
sp|Q96L96|ALPK3_HUMAN	Alpha-protein kinase 3 OS=Homo sapiens GN=ALPK3 PE=2 SV=2
sp|Q66K74|MAP1S_HUMAN	Microtubule-associated protein 1S OS=Homo sapiens GN=MAP1S PE=1 SV=2
sp|Q86V87|F16B2_HUMAN	Protein FAM160B2 OS=Homo sapiens GN=FAM160B2 PE=2 SV=2
sp|Q9P253|VPS18_HUMAN	Vacuolar protein sorting-associated protein 18 homolog OS=Homo sapiens GN=
sp|Q14896|MYPC3_HUMAN	Myosin-binding protein C cardiac-type OS=Homo sapiens GN=MYBPC3 PE=1 S
A0A0B4J1Y9|A0A0B4J1Y9_HUMAN	Protein IGHV3-72 (Fragment) OS=Homo sapiens GN=IGHV3-72 PE=1 SV=1
sp|P43652|AFAM_HUMAN	Afamin OS=Homo sapiens GN=AFM PE=1 SV=1
sp|P0C2W1|FBSP1_HUMAN	F-box/SPRY domain-containing protein 1 OS=Homo sapiens GN=FBXO45 PE=1
sp|P02751|FINC_HUMAN	Fibronectin OS=Homo sapiens GN=FN1 PE=1 SV=4
sp|P01764|HV323_HUMAN	Immunoglobulin heavy variable 3-23 OS=Homo sapiens GN=IGHV3-23 PE=1 SV
sp|Q16610|ECM1_HUMAN	Extracellular matrix protein 1 OS=Homo sapiens GN=ECM1 PE=1 SV=2
sp|P01599|KV107_HUMAN	Immunoglobulin kappa variable 1-17 OS=Homo sapiens GN=IGKV1-17 PE=1 SV
sp|Q92945|FUBP2_HUMAN	Far upstream element-binding protein 2 OS=Homo sapiens GN=KHSRP PE=1 S
sp|P02766|TTHY_HUMAN	Transthyretin OS=Homo sapiens GN=TTR PE=1 SV=1
sp|O94804|STK10_HUMAN	Serine/threonine-protein kinase 10 OS=Homo sapiens GN=STK10 PE=1 SV=1
sp|Q9UQD0|SCN8A_HUMAN	Sodium channel protein type 8 subunit alpha OS=Homo sapiens GN=SCN8A PE
sp|Q8N201|INT1_HUMAN	Integrator complex subunit 1 OS=Homo sapiens GN=INTS1 PE=1 SV=2
sp|Q9HCE3|ZN532_HUMAN	Zinc finger protein 532 OS=Homo sapiens GN=ZNF532 PE=1 SV=2
sp|Q8NB46|ANR52_HUMAN	Serine/threonine-protein phosphatase 6 regulatory ankyrin repeat subunit C OS=
sp|Q8TB24|RIN3_HUMAN	Ras and Rab interactor 3 OS=Homo sapiens GN=RIN3 PE=1 SV=4
sp|Q13129|RLF_HUMAN	Zinc finger protein Rlf OS=Homo sapiens GN=RLF PE=1 SV=2
sp|Q8NA03|FSIP1_HUMAN	Fibrous sheath-interacting protein 1 OS=Homo sapiens GN=FSIP1 PE=2 SV=1
sp|Q9NPG3|UBN1_HUMAN	Ubinuclein-1 OS=Homo sapiens GN=UBN1 PE=1 SV=2
sp|P01871|IGHM_HUMAN	Ig mu chain C region OS=Homo sapiens GN=IGHM PE=1 SV=3
A0A0B4J1U2|A0A0B4J1U2_HUMAN	Protein IGLV7-43 (Fragment) OS=Homo sapiens GN=IGLV7-43 PE=4 SV=1
sp|O43866|CD5L_HUMAN	CD5 antigen-like OS=Homo sapiens GN=CD5L PE=1 SV=1
sp|P06310|KV206_HUMAN	Ig kappa chain V-II region RPMI 6410 OS=Homo sapiens PE=4 SV=1
A0A0C4DH24|A0A0C4DH24_HUMAN	Protein IGKV6-21 (Fragment) OS=Homo sapiens GN=IGKV6-21 PE=1 SV=1
sp|Q15059|BRD3_HUMAN	Bromodomain-containing protein 3 OS=Homo sapiens GN=BRD3 PE=1 SV=1
sp|A6NDB9|PALM3_HUMAN	Paralemmin-3 OS=Homo sapiens GN=PALM3 PE=1 SV=2
sp|Q8IZF6|AGRG4_HUMAN	Adhesion G-protein coupled receptor G4 OS=Homo sapiens GN=ADGRG4 PE=2
sp|P08572|CO4A2_HUMAN	Collagen alpha-2(IV) chain OS=Homo sapiens GN=COL4A2 PE=1 SV=4
sp|P01859|IGHG2_HUMAN	Ig gamma-2 chain C region OS=Homo sapiens GN=IGHG2 PE=1 SV=2
A0A0B4J1V6|A0A0B4J1V6_HUMAN	Protein IGHV3-73 (Fragment) OS=Homo sapiens GN=IGHV3-73 PE=1 SV=1
sp|P01699|LV101_HUMAN	Ig lambda chain V-I region VOR OS=Homo sapiens PE=1 SV=1
sp|P01622|KV304_HUMAN	Ig kappa chain V-III region Ti OS=Homo sapiens PE=1 SV=1
sp|Q9BZ72|PITM2_HUMAN	Membrane-associated phosphatidylinositol transfer protein 2 OS=Homo sapiens
sp|P01023|A2MG_HUMAN	Alpha-2-macroglobulin OS=Homo sapiens GN=A2M PE=1 SV=3
sp|O14514|BAI1_HUMAN	Brain-specific angiogenesis inhibitor 1 OS=Homo sapiens GN=BAI1 PE=1 SV=2
sp|Q9HCU5|PREB_HUMAN	Prolactin regulatory element-binding protein OS=Homo sapiens GN=PREB PE=1
sp|Q9H251|CAD23_HUMAN	Cadherin-23 OS=Homo sapiens GN=CDH23 PE=1 SV=2
sp|P80748|LV302_HUMAN	Ig lambda chain V-III region LOI OS=Homo sapiens PE=1 SV=1
A0A0B4J1U7|A0A0B4J1U7_HUMAN	Protein IGHV6-1 (Fragment) OS=Homo sapiens GN=IGHV6-1 PE=4 SV=1
A0A075B6I0|A0A075B6I0_HUMAN	Protein IGLV8-61 (Fragment) OS=Homo sapiens GN=IGLV8-61 PE=1 SV=7
A0A0C4DH43|A0A0C4DH43_HUMAN	Uncharacterized protein (Fragment) OS=Homo sapiens PE=1 SV=1
sp|P01609|KV117_HUMAN	Ig kappa chain V-I region Scw OS=Homo sapiens PE=1 SV=1
sp|P01624|KV306_HUMAN	Ig kappa chain V-III region POM OS=Homo sapiens PE=1 SV=1
sp|Q8IXZ2|ZC3H3_HUMAN	Zinc finger CCCH domain-containing protein 3 OS=Homo sapiens GN=ZC3H3 P
sp|Q9UPM8|AP4E1_HUMAN	AP-4 complex subunit epsilon-1 OS=Homo sapiens GN=AP4E1 PE=1 SV=2
sp|Q05707|COEA1_HUMAN	Collagen alpha-1(XIV) chain OS=Homo sapiens GN=COL14A1 PE=1 SV=3
sp|Q9NT68|TEN2_HUMAN	Teneurin-2 OS=Homo sapiens GN=TENM2 PE=1 SV=3
sp|P0CG06|LAC3_HUMAN	Ig lambda-3 chain C regions OS=Homo sapiens GN=IGLC3 PE=1 SV=1
A0A075B6J3|A0A075B6J3_HUMAN	Protein IGLV3-27 (Fragment) OS=Homo sapiens GN=IGLV3-27 PE=1 SV=1
A0A0B4J2D9|A0A0B4J2D9_HUMAN	Protein IGKV1D-13 (Fragment) OS=Homo sapiens GN=IGKV1D-13 PE=1 SV=1
A0A0J9YXX1|A0A0J9YXX1_HUMAN	Uncharacterized protein (Fragment) OS=Homo sapiens PE=1 SV=1
A0A0C4DH68|A0A0C4DH68_HUMAN	Protein IGKV2-24 (Fragment) OS=Homo sapiens GN=IGKV2-24 PE=1 SV=1
sp|P01603|KV111_HUMAN	Ig kappa chain V-I region Ka OS=Homo sapiens PE=1 SV=1
sp|P01834|IGKC_HUMAN	Ig kappa chain C region OS=Homo sapiens GN=IGKC PE=1 SV=1
sp|Q9Y4D8|HECD4_HUMAN	Probable E3 ubiquitin-protein ligase HECTD4 OS=Homo sapiens GN=HECTD4
sp|Q99459|CDC5L_HUMAN	Cell division cycle 5-like protein OS=Homo sapiens GN=CDC5L PE=1 SV=2
A0A087WSY6|A0A087WSY6_HUMAN	Protein IGKV3D-15 (Fragment) OS=Homo sapiens GN=IGKV3D-15 PE=1 SV=6
sp|P01780|HV307_HUMAN	Immunoglobulin heavy variable 3-7 OS=Homo sapiens GN=IGHV3-7 PE=1 SV=2
sp|P06312|KV401_HUMAN	Immunoglobulin kappa variable 4-1 OS=Homo sapiens GN=IGKV4-1 PE=1 SV=1
A0A0C4DH25|A0A0C4DH25_HUMAN	Protein IGKV3D-20 (Fragment) OS=Homo sapiens GN=IGKV3D-20 PE=1 SV=1
sp|P01608|KV116_HUMAN	Ig kappa chain V-I region Roy OS=Homo sapiens PE=1 SV=1
sp|Q5SVQ8|ZBT41_HUMAN	Zinc finger and BTB domain-containing protein 41 OS=Homo sapiens GN=ZBTB
sp|Q8TAT5|NEIL3_HUMAN	Endonuclease 8-like 3 OS=Homo sapiens GN=NEIL3 PE=1 SV=3
sp|P43007|SATT_HUMAN	Neutral amino acid transporter A OS=Homo sapiens GN=SLC1A4 PE=1 SV=1
A0A0J9YY99|A0A0J9YY99_HUMAN	Uncharacterized protein (Fragment) OS=Homo sapiens PE=1 SV=1
sp|Q15149|PLEC_HUMAN	Plectin OS=Homo sapiens GN=PLEC PE=1 SV=3
A0A0G2JRQ6|A0A0G2JRQ6_HUMAN	Uncharacterized protein (Fragment) OS=Homo sapiens PE=1 SV=4
sp|Q8IZY2|ABCA7_HUMAN	ATP-binding cassette sub-family A member 7 OS=Homo sapiens GN=ABCA7 PE
sp|Q08380|LG3BP_HUMAN	Galectin-3-binding protein OS=Homo sapiens GN=LGALS3BP PE=1 SV=1
sp|Q9HBE1|PATZ1_HUMAN	POZ- AT hook- and zinc finger-containing protein 1 OS=Homo sapiens GN=PATZ
sp|P98169|ZXDB_HUMAN	Zinc finger X-linked protein ZXDB OS=Homo sapiens GN=ZXDB PE=2 SV=2
sp|Q8N2Y8|RUSC2_HUMAN	Iporin OS=Homo sapiens GN=RUSC2 PE=1 SV=3
sp|Q7Z6Z7|HUWE1_HUMAN	E3 ubiquitin-protein ligase HUWE1 OS=Homo sapiens GN=HUWE1 PE=1 SV=3
sp|Q8NI60|ADCK3_HUMAN	Atypical kinase ADCK3 mitochondrial OS=Homo sapiens GN=ADCK3 PE=1 SV=
sp|Q8N7J2|AMER2_HUMAN	APC membrane recruitment protein 2 OS=Homo sapiens GN=AMER2 PE=1 SV=
sp|P01762|HV311_HUMAN	Immunoglobulin heavy variable 3-11 OS=Homo sapiens GN=IGHV3-11 PE=1 SV
sp|Q9UPT8|ZC3H4_HUMAN	Zinc finger CCCH domain-containing protein 4 OS=Homo sapiens GN=ZC3H4 P
sp|P41229|KDM5C_HUMAN	Lysine-specific demethylase 5C OS=Homo sapiens GN=KDM5C PE=1 SV=2
sp|O00515|LAD1_HUMAN	Ladinin-1 OS=Homo sapiens GN=LAD1 PE=1 SV=2
sp|Q9NQX3|GEPH_HUMAN	Gephyrin OS=Homo sapiens GN=GPHN PE=1 SV=1
sp|P35222|CTNB1_HUMAN	Catenin beta-1 OS=Homo sapiens GN=CTNNB1 PE=1 SV=1
sp|P01833|PIGR_HUMAN	Polymeric immunoglobulin receptor OS=Homo sapiens GN=PIGR PE=1 SV=4
sp|P30613|KPYR_HUMAN	Pyruvate kinase PKLR OS=Homo sapiens GN=PKLR PE=1 SV=2
sp|Q9Y467|SALL2_HUMAN	Sal-like protein 2 OS=Homo sapiens GN=SALL2 PE=1 SV=4
sp|P01616|KV203_HUMAN	Ig kappa chain V-II region MIL OS=Homo sapiens PE=1 SV=1
sp|Q9HC10|OTOF_HUMAN	Otoferlin OS=Homo sapiens GN=OTOF PE=1 SV=3
sp|O75976|CBPD_HUMAN	Carboxypeptidase D OS=Homo sapiens GN=CPD PE=1 SV=2
sp|P02750|A2GL_HUMAN	Leucine-rich alpha-2-glycoprotein OS=Homo sapiens GN=LRG1 PE=1 SV=2
sp|Q2M1P5|KIF7_HUMAN	Kinesin-like protein KIF7 OS=Homo sapiens GN=KIF7 PE=1 SV=2
sp|P04004|VTNC_HUMAN	Vitronectin OS=Homo sapiens GN=VTN PE=1 SV=1
sp|P02741|CRP_HUMAN	C-reactive protein OS=Homo sapiens GN=CRP PE=1 SV=1
sp|P01009|A1AT_HUMAN	Alpha-1-antitrypsin OS=Homo sapiens GN=SERPINA1 PE=1 SV=3
sp|Q92954|PRG4_HUMAN	Proteoglycan 4 OS=Homo sapiens GN=PRG4 PE=1 SV=2
sp|Q5T011|SZT2_HUMAN	Protein SZT2 OS=Homo sapiens GN=SZT2 PE=1 SV=3
sp|Q5JWF2|GNAS1_HUMAN	Guanine nucleotide-binding protein G(s) subunit alpha isoforms XLas OS=Homo
sp|Q12802|AKP13_HUMAN	A-kinase anchor protein 13 OS=Homo sapiens GN=AKAP13 PE=1 SV=2
sp|O00716|E2F3_HUMAN	Transcription factor E2F3 OS=Homo sapiens GN=E2F3 PE=1 SV=1
sp|Q14241|ELOA1_HUMAN	Transcription elongation factor B polypeptide 3 OS=Homo sapiens GN=TCEB3 P
sp|O94913|PCF11_HUMAN	Pre-mRNA cleavage complex 2 protein Pcf11 OS=Homo sapiens GN=PCF11 PE
A0A1B0GTH6|A0A1B0GTH6_HUMAN	Protein LOC100506944 OS=Homo sapiens GN=LOC100506944 PE=4 SV=1
sp|Q14004|CDK13_HUMAN	Cyclin-dependent kinase 13 OS=Homo sapiens GN=CDK13 PE=1 SV=2
sp|Q96ST3|SIN3A_HUMAN	Paired amphipathic helix protein Sin3a OS=Homo sapiens GN=SIN3A PE=1 SV=
sp|Q13434|MKRN4_HUMAN	Putative E3 ubiquitin-protein ligase makorin-4 OS=Homo sapiens GN=MKRN4P
sp|P15923|TFE2_HUMAN	Transcription factor E2-alpha OS=Homo sapiens GN=TCF3 PE=1 SV=1
sp|P16070|CD44_HUMAN	CD44 antigen OS=Homo sapiens GN=CD44 PE=1 SV=3
sp|O15439|MRP4_HUMAN	Multidrug resistance-associated protein 4 OS=Homo sapiens GN=ABCC4 PE=1

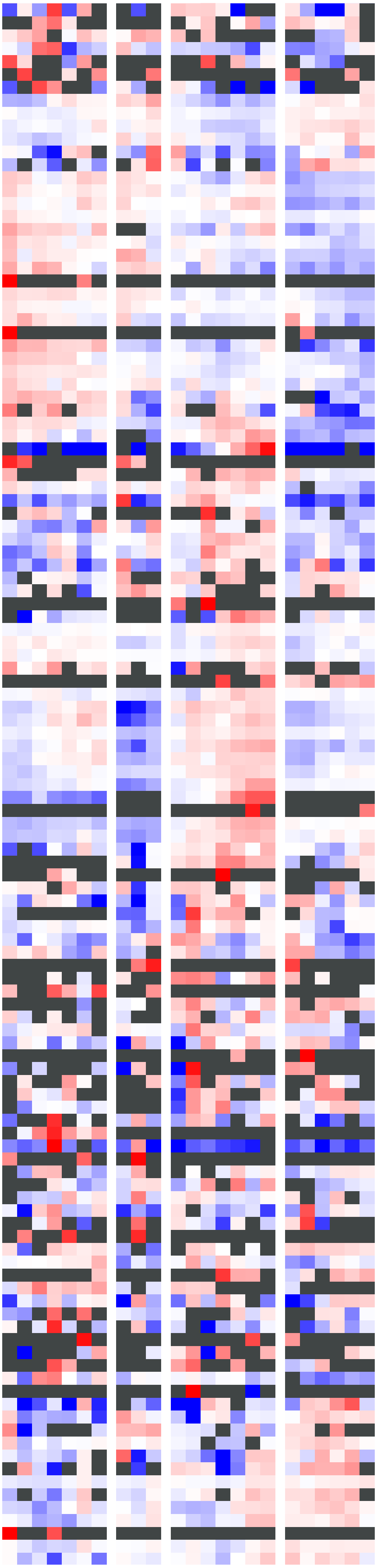

spIO95996IAPC2_HUMAN	Adenomatous polyposis coli protein 2 OS=Homo sapiens GN=APC2 PE=1 SV=1
spIQ13057ICOASY_HUMAN	Bifunctional coenzyme A synthase OS=Homo sapiens GN=COASY PE=1 SV=4
A0A0U1RR37IA0A0U1RR37_HUMAN	Protein LOC105376887 OS=Homo sapiens GN=LOC105376887 PE=4 SV=1
spIP01593IKV101_HUMAN	Ig kappa chain V-I region AG OS=Homo sapiens PE=1 SV=1
spIP48740IMASP1_HUMAN	Mannan-binding lectin serine protease 1 OS=Homo sapiens GN=MASP1 PE=1 S
spIP33151ICADH5_HUMAN	Cadherin-5 OS=Homo sapiens GN=CDH5 PE=1 SV=5
spIQ2Q1W2ILIN41_HUMAN	E3 ubiquitin-protein ligase TRIM71 OS=Homo sapiens GN=TRIM71 PE=1 SV=1
spIQ96PD5IPGRP2_HUMAN	N-acetylmuramoyl-L-alanine amidase OS=Homo sapiens GN=PGLYRP2 PE=1 S
spIP02768IALBU_HUMAN	Serum albumin OS=Homo sapiens GN=ALB PE=1 SV=2
spIP02790IHEMO_HUMAN	Hemopexin OS=Homo sapiens GN=HPX PE=1 SV=2
spIQ8NFW1ICOMA1_HUMAN	Collagen alpha-1(XXII) chain OS=Homo sapiens GN=COL22A1 PE=2 SV=2
spIP13645IK1C10_HUMAN	Keratin type I cytoskeletal 10 OS=Homo sapiens GN=KRT10 PE=1 SV=6
spIQ9H172IABCG4_HUMAN	ATP-binding cassette sub-family G member 4 OS=Homo sapiens GN=ABCG4 PE
A0A096LPE2IA0A096LPE2_HUMAN	Protein SAA2-SAA4 OS=Homo sapiens GN=SAA2-SAA4 PE=4 SV=1
spIP00734ITHRB_HUMAN	Prothrombin OS=Homo sapiens GN=F2 PE=1 SV=2
A0A0A0MT74IA0A0A0MT74_HUMAN	Protein IGKV1-16 (Fragment) OS=Homo sapiens GN=IGKV1-16 PE=1 SV=1
spIP00450ICERU_HUMAN	Ceruloplasmin OS=Homo sapiens GN=CP PE=1 SV=1
spIQ9H269IVPS16_HUMAN	Vacuolar protein sorting-associated protein 16 homolog OS=Homo sapiens GN=\
spIP02748ICO9_HUMAN	Complement component C9 OS=Homo sapiens GN=C9 PE=1 SV=2
A0A075B6Q5IA0A075B6Q5_HUMAN	Protein IGHV3-64 (Fragment) OS=Homo sapiens GN=IGHV3-64 PE=1 SV=1
spIP01767IHV353_HUMAN	Immunoglobulin heavy variable 3-53 OS=Homo sapiens GN=IGHV3-53 PE=1 SV
spIQ04756IHGFA_HUMAN	Hepatocyte growth factor activator OS=Homo sapiens GN=HGFAC PE=1 SV=1
B4E1Z4IB4E1Z4_HUMAN	Uncharacterized protein OS=Homo sapiens PE=1 SV=1
spIP04217IA1BG_HUMAN	Alpha-1B-glycoprotein OS=Homo sapiens GN=A1BG PE=1 SV=4
spIQ14624IITIH4_HUMAN	Inter-alpha-trypsin inhibitor heavy chain H4 OS=Homo sapiens GN=ITIH4 PE=1 S
spIQ14678IKANK1_HUMAN	KN motif and ankyrin repeat domain-containing protein 1 OS=Homo sapiens GN=
spIO75037IKI21B_HUMAN	Kinesin-like protein KIF21B OS=Homo sapiens GN=KIF21B PE=1 SV=2
spIP09871IC1S_HUMAN	Complement C1s subcomponent OS=Homo sapiens GN=C1S PE=1 SV=1
spIP13671ICO6_HUMAN	Complement component C6 OS=Homo sapiens GN=C6 PE=1 SV=3
spIA6NMZ7ICO6A6_HUMAN	Collagen alpha-6(VI) chain OS=Homo sapiens GN=COL6A6 PE=1 SV=2
spIQ96EB6ISIR1_HUMAN	NAD-dependent protein deacetylase sirtuin-1 OS=Homo sapiens GN=SIRT1 PE=
spIP31629IZEP2_HUMAN	Transcription factor HIVEP2 OS=Homo sapiens GN=HIVEP2 PE=1 SV=2
spIQ06033IITIH3_HUMAN	Inter-alpha-trypsin inhibitor heavy chain H3 OS=Homo sapiens GN=ITIH3 PE=1 S
spIP01619IKV301_HUMAN	Ig kappa chain V-III region B6 OS=Homo sapiens PE=1 SV=1
spIP01605IKV113_HUMAN	Ig kappa chain V-I region Lay OS=Homo sapiens PE=1 SV=1
spIQ9HBG6IIF122_HUMAN	Intraflagellar transport protein 122 homolog OS=Homo sapiens GN=IFT122 PE=1
spIQ8TB72IPUM2_HUMAN	Pumilio homolog 2 OS=Homo sapiens GN=PUM2 PE=1 SV=2
spIP45844IABCG1_HUMAN	ATP-binding cassette sub-family G member 1 OS=Homo sapiens GN=ABCG1 PE
spIQ7Z5P9IMUC19_HUMAN	Mucin-19 OS=Homo sapiens GN=MUC19 PE=1 SV=3
spIQ6P1M9IARMX5_HUMAN	Armadillo repeat-containing X-linked protein 5 OS=Homo sapiens GN=ARMCX5 I
spIP01782IHV321_HUMAN	Ig heavy chain V-III region DOB OS=Homo sapiens PE=1 SV=1
spIP06727IAPOA4_HUMAN	Apolipoprotein A-IV OS=Homo sapiens GN=APOA4 PE=1 SV=3
spIO75815IBCAR3_HUMAN	Breast cancer anti-estrogen resistance protein 3 OS=Homo sapiens GN=BCAR3
spIQ2LD37IK1109_HUMAN	Uncharacterized protein KIAA1109 OS=Homo sapiens GN=KIAA1109 PE=1 SV=:
spIQ9ULH4ILRFN2_HUMAN	Leucine-rich repeat and fibronectin type-III domain-containing protein 2 OS=Homo
spIP57679IEVC_HUMAN	Ellis-van Creveld syndrome protein OS=Homo sapiens GN=EVC PE=1 SV=1
spIQ8TBB5IKLDC4_HUMAN	Kelch domain-containing protein 4 OS=Homo sapiens GN=KLHDC4 PE=1 SV=1
spIQ01484IANK2_HUMAN	Ankyrin-2 OS=Homo sapiens GN=ANK2 PE=1 SV=4
A0A0A0MS15IA0A0A0MS15_HUMAN	Protein IGHV3-49 (Fragment) OS=Homo sapiens GN=IGHV3-49 PE=1 SV=1
spIQ9C0A1IZFHX2_HUMAN	Zinc finger homeobox protein 2 OS=Homo sapiens GN=ZFHX2 PE=2 SV=3
spIP02760IAMBP_HUMAN	Protein AMBP OS=Homo sapiens GN=AMBP PE=1 SV=1
spIA5YKK6ICNOT1_HUMAN	CCR4-NOT transcription complex subunit 1 OS=Homo sapiens GN=CNOT1 PE=
spIP08294ISODE_HUMAN	Extracellular superoxide dismutase [Cu-Zn] OS=Homo sapiens GN=SOD3 PE=1
spIP01019IANGT_HUMAN	Angiotensinogen OS=Homo sapiens GN=AGT PE=1 SV=1
A0A0C4DH69IA0A0C4DH69_HUMAN	Protein IGKV1-9 (Fragment) OS=Homo sapiens GN=IGKV1-9 PE=4 SV=1
spIP01602IKV110_HUMAN	Immunoglobulin kappa variable 1-5 OS=Homo sapiens GN=IGKV1-5 PE=1 SV=2
spIP04433IKV309_HUMAN	Ig kappa chain V-III region VG (Fragment) OS=Homo sapiens PE=1 SV=1
spIP04208ILV106_HUMAN	Ig lambda chain V-I region WAH OS=Homo sapiens PE=1 SV=1
spIQ9Y490ITLN1_HUMAN	Talin-1 OS=Homo sapiens GN=TLN1 PE=1 SV=3
spIP01857IIGHG1_HUMAN	Ig gamma-1 chain C region OS=Homo sapiens GN=IGHG1 PE=1 SV=1
A0A0C4DH31IA0A0C4DH31_HUMAN	Protein IGHV1-18 (Fragment) OS=Homo sapiens GN=IGHV1-18 PE=1 SV=1
A0A0G2JMI3IA0A0G2JMI3_HUMAN	Uncharacterized protein (Fragment) OS=Homo sapiens PE=1 SV=2
spIQ8TF39IZN483_HUMAN	Zinc finger protein 483 OS=Homo sapiens GN=ZNF483 PE=1 SV=3
spIP29120INEC1_HUMAN	Neuroendocrine convertase 1 OS=Homo sapiens GN=PCSK1 PE=1 SV=2
A0A075B6S5IA0A075B6S5_HUMAN	Protein IGKV1-27 (Fragment) OS=Homo sapiens GN=IGKV1-27 PE=1 SV=1
spIP53420ICO4A4_HUMAN	Collagen alpha-4(IV) chain OS=Homo sapiens GN=COL4A4 PE=1 SV=3
spIQ5VWW1IC1QL3_HUMAN	Complement C1q-like protein 3 OS=Homo sapiens GN=C1QL3 PE=2 SV=1
spIQ9NY28IGALT8_HUMAN	Probable polypeptide N-acetylgalactosaminyltransferase 8 OS=Homo sapiens GN
spIQ9UNE7ICHIP_HUMAN	E3 ubiquitin-protein ligase CHIP OS=Homo sapiens GN=STUB1 PE=1 SV=2
spIO94856INFASC_HUMAN	Neurofascin OS=Homo sapiens GN=NFASC PE=1 SV=4
spIQ96AX9IMIB2_HUMAN	E3 ubiquitin-protein ligase MIB2 OS=Homo sapiens GN=MIB2 PE=1 SV=3
spIQ5VVW2IGARL3_HUMAN	GTPase-activating Rap/Ran-GAP domain-like protein 3 OS=Homo sapiens GN=(
spIP08123ICO1A2_HUMAN	Collagen alpha-2(I) chain OS=Homo sapiens GN=COL1A2 PE=1 SV=7
spIP0DJI9ISAA2_HUMAN	Serum amyloid A-2 protein OS=Homo sapiens GN=SAA2 PE=1 SV=1
spIQ15147IPLCB4_HUMAN	1-phosphatidylinositol 4 5-bisphosphate phosphodiesterase beta-4 OS=Homo sa
spIP61769IB2MG_HUMAN	Beta-2-microglobulin OS=Homo sapiens GN=B2M PE=1 SV=1
spIO43900IPRIC3_HUMAN	Prickle-like protein 3 OS=Homo sapiens GN=PRICKLE3 PE=1 SV=2
spIQ08AD1ICAMP2_HUMAN	Calmodulin-regulated spectrin-associated protein 2 OS=Homo sapiens GN=CAM
spIQ8TEK3IDOT1L_HUMAN	Histone-lysine N-methyltransferase H3 lysine-79 specific OS=Homo sapiens GN=
spIQ9Y6Z5IMLAS1_HUMAN	Putative uncharacterized protein MLLT4-AS1 OS=Homo sapiens GN=MLLT4-AS
spIP25940ICO5A3_HUMAN	Collagen alpha-3(V) chain OS=Homo sapiens GN=COL5A3 PE=1 SV=3
spIP08779IK1C16_HUMAN	Keratin type I cytoskeletal 16 OS=Homo sapiens GN=KRT16 PE=1 SV=4
spIQ86YA3IZGRF1_HUMAN	Protein ZGRF1 OS=Homo sapiens GN=ZGRF1 PE=1 SV=3
spIQ8IZ69ITRM2A_HUMAN	tRNA (uracil-5-)-methyltransferase homolog A OS=Homo sapiens GN=TRMT2A I
spIQ02446ISP4_HUMAN	Transcription factor Sp4 OS=Homo sapiens GN=SP4 PE=1 SV=2
spIO95954IFTCD_HUMAN	Formimidoyltransferase-cyclodeaminase OS=Homo sapiens GN=FTCD PE=1 SV
spIP00915ICAH1_HUMAN	Carbonic anhydrase 1 OS=Homo sapiens GN=CA1 PE=1 SV=2
spIP11137IMTAP2_HUMAN	Microtubule-associated protein 2 OS=Homo sapiens GN=MAP2 PE=1 SV=4
spIP68871IHBB_HUMAN	Hemoglobin subunit beta OS=Homo sapiens GN=HBB PE=1 SV=2
spIQ96I13IABHD8_HUMAN	Protein ABHD8 OS=Homo sapiens GN=ABHD8 PE=2 SV=1
spIQ99973ITEP1_HUMAN	Telomerase protein component 1 OS=Homo sapiens GN=TEP1 PE=1 SV=2
spIQ9H2B4IS26A1_HUMAN	Sulfate anion transporter 1 OS=Homo sapiens GN=SLC26A1 PE=1 SV=2
spIO14776ITCRG1_HUMAN	Transcription elongation regulator 1 OS=Homo sapiens GN=TCERG1 PE=1 SV=:
spIQ02388ICO7A1_HUMAN	Collagen alpha-1(VII) chain OS=Homo sapiens GN=COL7A1 PE=1 SV=2
spIQ13127IREST_HUMAN	RE1-silencing transcription factor OS=Homo sapiens GN=REST PE=1 SV=3
spIQ86UL8IMAGI2_HUMAN	Membrane-associated guanylate kinase WW and PDZ domain-containing protein
spIO60493ISNX3_HUMAN	Sorting nexin-3 OS=Homo sapiens GN=SNX3 PE=1 SV=3
spIO75128ICOBL_HUMAN	Protein cordon-bleu OS=Homo sapiens GN=COBL PE=1 SV=2
spIQ12805IFBLN3_HUMAN	EGF-containing fibulin-like extracellular matrix protein 1 OS=Homo sapiens GN=E
spIQ8NFD5IARI1B_HUMAN	AT-rich interactive domain-containing protein 1B OS=Homo sapiens GN=ARID1B
spIP07202IPERT_HUMAN	Thyroid peroxidase OS=Homo sapiens GN=TPO PE=1 SV=4
spIQ9UHB9ISRP68_HUMAN	Signal recognition particle subunit SRP68 OS=Homo sapiens GN=SRP68 PE=1 S
spIP32119IPRDX2_HUMAN	Peroxiredoxin-2 OS=Homo sapiens GN=PRDX2 PE=1 SV=5
spIP01701ILV151_HUMAN	Immunoglobulin lambda variable 1-51 OS=Homo sapiens GN=IGLV1-51 PE=1 S\
spIA7KAX9IRHG32_HUMAN	Rho GTPase-activating protein 32 OS=Homo sapiens GN=ARHGAP32 PE=1 SV
spIQ8IWI9IMGAP_HUMAN	MAX gene-associated protein OS=Homo sapiens GN=MGA PE=1 SV=3
spIQ14993ICOJA1_HUMAN	Collagen alpha-1(XIX) chain OS=Homo sapiens GN=COL19A1 PE=1 SV=3
spIP27708IPYR1_HUMAN	CAD protein OS=Homo sapiens GN=CAD PE=1 SV=3
spIO15021IMAST4_HUMAN	Microtubule-associated serine/threonine-protein kinase 4 OS=Homo sapiens GN=
spIO75334ILIPA2_HUMAN	Liprin-alpha-2 OS=Homo sapiens GN=PPFIA2 PE=1 SV=2
spIQ9Y6N6ILAMC3_HUMAN	Laminin subunit gamma-3 OS=Homo sapiens GN=LAMC3 PE=1 SV=3
spIQ9UBW5IBIN2_HUMAN	Bridging integrator 2 OS=Homo sapiens GN=BIN2 PE=1 SV=3
spIP01719ILV501_HUMAN	Ig lambda chain V-V region DEL OS=Homo sapiens PE=1 SV=1
spIQ8IVT5IKSR1_HUMAN	Kinase suppressor of Ras 1 OS=Homo sapiens GN=KSR1 PE=1 SV=3
spIP02787ITRFE_HUMAN	Serotransferrin OS=Homo sapiens GN=TF PE=1 SV=3
spIP36896IACV1B_HUMAN	Activin receptor type-1B OS=Homo sapiens GN=ACVR1B PE=1 SV=1
spIP02656IAPOC3_HUMAN	Apolipoprotein C-III OS=Homo sapiens GN=APOC3 PE=1 SV=1
spIO95785IWIZ_HUMAN	Protein Wiz OS=Homo sapiens GN=WIZ PE=1 SV=2
spIQ86TL0IATG4D_HUMAN	Cysteine protease ATG4D OS=Homo sapiens GN=ATG4D PE=1 SV=1
spIQ01831IXPC_HUMAN	DNA repair protein complementing XP-C cells OS=Homo sapiens GN=XPC PE=1
spIP20848IA1ATR_HUMAN	Putative alpha-1-antitrypsin-related protein OS=Homo sapiens GN=SERPINA2 P

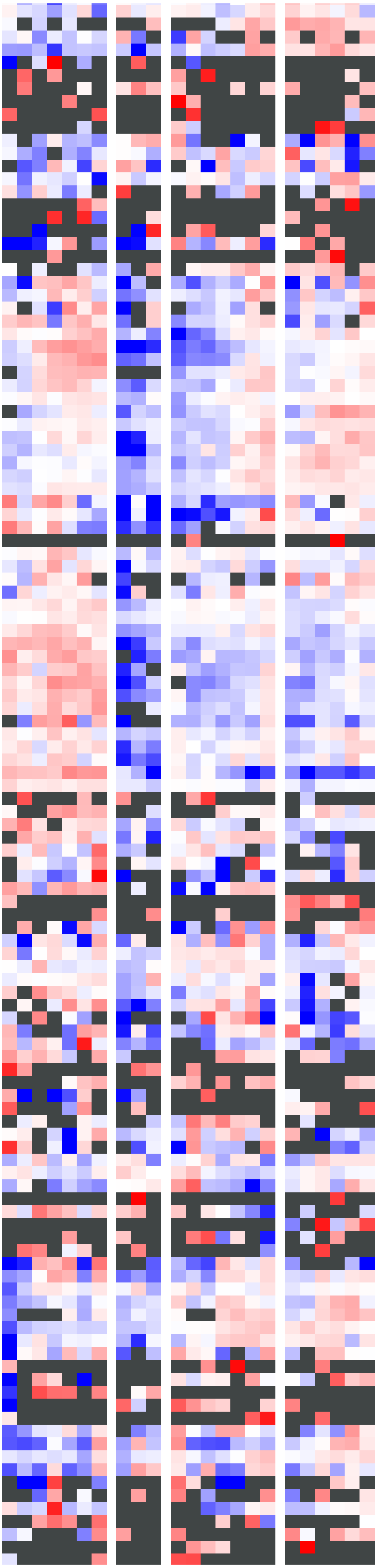

[illegible]	[illegible]
sp|P55287|CAD11_HUMAN	Cadherin-11 OS=Homo sapiens GN=CDH11 PE=2 SV=2
sp|Q99466|NOTC4_HUMAN	Neurogenic locus notch homolog protein 4 OS=Homo sapiens GN=NOTCH4 PE=
sp|O75154|RFIP3_HUMAN	Rab11 family-interacting protein 3 OS=Homo sapiens GN=RAB11FIP3 PE=1 SV=
sp|Q5GLZ8|HERC4_HUMAN	Probable E3 ubiquitin-protein ligase HERC4 OS=Homo sapiens GN=HERC4 PE=
sp|P04040|CATA_HUMAN	Catalase OS=Homo sapiens GN=CAT PE=1 SV=3
sp|P00746|CFAD_HUMAN	Complement factor D OS=Homo sapiens GN=CFD PE=1 SV=5
sp|Q5VUA4|ZN318_HUMAN	Zinc finger protein 318 OS=Homo sapiens GN=ZNF318 PE=1 SV=2
sp|P05543|THBG_HUMAN	Thyroxine-binding globulin OS=Homo sapiens GN=SERPINA7 PE=1 SV=2
sp|O15061|SYNEM_HUMAN	Synemin OS=Homo sapiens GN=SYNM PE=1 SV=2
A0A0J9YXQ4|A0A0J9YXQ4_HUMAN	Uncharacterized protein OS=Homo sapiens PE=1 SV=1
sp|Q8N715|CC185_HUMAN	Coiled-coil domain-containing protein 185 OS=Homo sapiens GN=CCDC185 PE=
sp|Q9Y2T7|YBOX2_HUMAN	Y-box-binding protein 2 OS=Homo sapiens GN=YBX2 PE=1 SV=2
sp|Q01082|SPTB2_HUMAN	Spectrin beta chain non-erythrocytic 1 OS=Homo sapiens GN=SPTBN1 PE=1 SV
sp|Q9P2P6|STAR9_HUMAN	StAR-related lipid transfer protein 9 OS=Homo sapiens GN=STARD9 PE=1 SV=3
sp|O14686|KMT2D_HUMAN	Histone-lysine N-methyltransferase 2D OS=Homo sapiens GN=KMT2D PE=1 SV
sp|Q9NSI6|BRWD1_HUMAN	Bromodomain and WD repeat-containing protein 1 OS=Homo sapiens GN=BRWI
sp|Q9HCJ0|TNR6C_HUMAN	Trinucleotide repeat-containing gene 6C protein OS=Homo sapiens GN=TNRC6C
sp|P51587|BRCA2_HUMAN	Breast cancer type 2 susceptibility protein OS=Homo sapiens GN=BRCA2 PE=1
sp|Q96JG9|ZN469_HUMAN	Zinc finger protein 469 OS=Homo sapiens GN=ZNF469 PE=2 SV=3
sp|Q96DF8|DGC14_HUMAN	Protein DGCR14 OS=Homo sapiens GN=DGCR14 PE=1 SV=1
sp|Q9BXT5|TEX15_HUMAN	Testis-expressed sequence 15 protein OS=Homo sapiens GN=TEX15 PE=2 SV=
A0A0C4DH90|A0A0C4DH90_HUMAN	Protein IGKV3OR2-268 (Fragment) OS=Homo sapiens GN=IGKV3OR2-268 PE=
sp|P11047|LAMC1_HUMAN	Laminin subunit gamma-1 OS=Homo sapiens GN=LAMC1 PE=1 SV=3
sp|P13647|K2C5_HUMAN	Keratin type II cytoskeletal 5 OS=Homo sapiens GN=KRT5 PE=1 SV=3
sp|P25440|BRD2_HUMAN	Bromodomain-containing protein 2 OS=Homo sapiens GN=BRD2 PE=1 SV=2
A0A0B4J1U3|A0A0B4J1U3_HUMAN	Protein IGLV1-36 (Fragment) OS=Homo sapiens GN=IGLV1-36 PE=1 SV=5
A0A0B4J1V0|A0A0B4J1V0_HUMAN	Protein IGHV3-15 (Fragment) OS=Homo sapiens GN=IGHV3-15 PE=1 SV=1
A0A0B4J1Z7|A0A0B4J1Z7_HUMAN	Protein IGKV1-39 (Fragment) OS=Homo sapiens GN=IGKV1-39 PE=4 SV=1
A0A0B4J1X5|A0A0B4J1X5_HUMAN	Protein IGHV3-74 (Fragment) OS=Homo sapiens GN=IGHV3-74 PE=1 SV=1
A0A075B6H9|A0A075B6H9_HUMAN	Protein IGLV4-69 (Fragment) OS=Homo sapiens GN=IGLV4-69 PE=1 SV=1
sp|Q7RTR2|NLRC3_HUMAN	Protein NLRC3 OS=Homo sapiens GN=NLRC3 PE=2 SV=2
A0A075B6K3|A0A075B6K3_HUMAN	Protein IGLV2-11 (Fragment) OS=Homo sapiens GN=IGLV2-11 PE=1 SV=1
sp|P25311|ZA2G_HUMAN	Zinc-alpha-2-glycoprotein OS=Homo sapiens GN=AZGP1 PE=1 SV=2
A0A075B6K4|A0A075B6K4_HUMAN	HCG2043238 (Fragment) OS=Homo sapiens GN=IGLV3-10 PE=1 SV=1
A0A075B6J0|A0A075B6J0_HUMAN	Protein IGLV1-40 (Fragment) OS=Homo sapiens GN=IGLV1-40 PE=1 SV=7
sp|P0DTD1|R1AB_SARS2	Replicase polyprotein 1ab OS=Severe acute respiratory syndrome coronavirus 2
sp|P35968|VGFR2_HUMAN	Vascular endothelial growth factor receptor 2 OS=Homo sapiens GN=KDR PE=1
sp|P01766|HV313_HUMAN	Immunoglobulin heavy variable 3-13 OS=Homo sapiens GN=IGHV3-13 PE=1 SV
sp|Q9H1R3|MYLK2_HUMAN	Myosin light chain kinase 2 skeletal/cardiac muscle OS=Homo sapiens GN=MYLI
sp|Q9BZC7|ABCA2_HUMAN	ATP-binding cassette sub-family A member 2 OS=Homo sapiens GN=ABCA2 PE
sp|Q9BXR6|FHR5_HUMAN	Complement factor H-related protein 5 OS=Homo sapiens GN=CFHR5 PE=1 SV
sp|Q8N3T1|GLT15_HUMAN	Polypeptide N-acetylgalactosaminyltransferase 15 OS=Homo sapiens GN=GALN
sp|Q9Y4F3|MARF1_HUMAN	Meiosis arrest female protein 1 OS=Homo sapiens GN=KIAA0430 PE=1 SV=6
sp|Q9NQT8|KI13B_HUMAN	Kinesin-like protein KIF13B OS=Homo sapiens GN=KIF13B PE=1 SV=2
sp|A8TX70|CO6A5_HUMAN	Collagen alpha-5(VI) chain OS=Homo sapiens GN=COL6A5 PE=1 SV=1
sp|Q9H8M5|CNNM2_HUMAN	Metal transporter CNNM2 OS=Homo sapiens GN=CNNM2 PE=1 SV=2
sp|P20851|C4BPB_HUMAN	C4b-binding protein beta chain OS=Homo sapiens GN=C4BPB PE=1 SV=1
sp|P0CG05|LAC2_HUMAN	Ig lambda-2 chain C regions OS=Homo sapiens GN=IGLC2 PE=1 SV=1
A0A0C4DH38|A0A0C4DH38_HUMAN	Protein IGHV5-51 (Fragment) OS=Homo sapiens GN=IGHV5-51 PE=1 SV=1
sp|O75145|LIPA3_HUMAN	Liprin-alpha-3 OS=Homo sapiens GN=PPFIA3 PE=1 SV=3
sp|Q14055|CO9A2_HUMAN	Collagen alpha-2(IX) chain OS=Homo sapiens GN=COL9A2 PE=1 SV=2
sp|Q9UN73|PCDA6_HUMAN	Protocadherin alpha-6 OS=Homo sapiens GN=PCDHA6 PE=2 SV=1
A0A0J2H0|A0A0J2H0_HUMAN	Protein IGHV1-69-2 (Fragment) OS=Homo sapiens GN=IGHV1-69-2 PE=1 SV=1
sp|Q3B7T1|EDRF1_HUMAN	Erythroid differentiation-related factor 1 OS=Homo sapiens GN=EDRF1 PE=1 SV
sp|Q9UPY8|MARE3_HUMAN	Microtubule-associated protein RP/EB family member 3 OS=Homo sapiens GN=I
sp|A9Z1Z3|FR1L4_HUMAN	Fer-1-like protein 4 OS=Homo sapiens GN=FER1L4 PE=2 SV=1
A0A0J9YX35|A0A0J9YX35_HUMAN	Uncharacterized protein (Fragment) OS=Homo sapiens PE=1 SV=1
sp|P21333|FLNA_HUMAN	Filamin-A OS=Homo sapiens GN=FLNA PE=1 SV=4
sp|Q9NYV4|CDK12_HUMAN	Cyclin-dependent kinase 12 OS=Homo sapiens GN=CDK12 PE=1 SV=2
sp|P01763|HV348_HUMAN	Immunoglobulin heavy variable 3-48 OS=Homo sapiens GN=IGHV3-48 PE=1 SV
sp|P07225|PROS_HUMAN	Vitamin K-dependent protein S OS=Homo sapiens GN=PROS1 PE=1 SV=1
sp|Q94819|KBTBB_HUMAN	Kelch repeat and BTB domain-containing protein 11 OS=Homo sapiens GN=KBT
sp|P02654|APOC1_HUMAN	Apolipoprotein C-I OS=Homo sapiens GN=APOC1 PE=1 SV=1
sp|Q6ZT07|TBCD9_HUMAN	TBC1 domain family member 9 OS=Homo sapiens GN=TBC1D9 PE=2 SV=2
sp|P52951|GBX2_HUMAN	Homeobox protein GBX-2 OS=Homo sapiens GN=GBX2 PE=2 SV=3
sp|Q96JE9|MAP6_HUMAN	Microtubule-associated protein 6 OS=Homo sapiens GN=MAP6 PE=1 SV=2
A0A0C4DH32|A0A0C4DH32_HUMAN	Protein IGHV3-20 (Fragment) OS=Homo sapiens GN=IGHV3-20 PE=1 SV=1
sp|Q8IZU2|WDR17_HUMAN	WD repeat-containing protein 17 OS=Homo sapiens GN=WDR17 PE=2 SV=2
sp|P06681|CO2_HUMAN	Complement C2 OS=Homo sapiens GN=C2 PE=1 SV=2
sp|Q14676|MDC1_HUMAN	Mediator of DNA damage checkpoint protein 1 OS=Homo sapiens GN=MDC1 PE
sp|P30566|PUR8_HUMAN	Adenylosuccinate lyase OS=Homo sapiens GN=ADSL PE=1 SV=2
sp|O60645|EXOC3_HUMAN	Exocyst complex component 3 OS=Homo sapiens GN=EXOC3 PE=1 SV=2
sp|O15417|TNC18_HUMAN	Trinucleotide repeat-containing gene 18 protein OS=Homo sapiens GN=TNRC18
sp|Q9P2D3|HTR5B_HUMAN	HEAT repeat-containing protein 5B OS=Homo sapiens GN=HEATR5B PE=1 SV=
sp|P01620|KV302_HUMAN	Ig kappa chain V-III region SIE OS=Homo sapiens PE=1 SV=1
sp|Q8IVF2|AHNK2_HUMAN	Protein AHNAK2 OS=Homo sapiens GN=AHNAK2 PE=1 SV=2
sp|Q9ULT8|HECD1_HUMAN	E3 ubiquitin-protein ligase HECTD1 OS=Homo sapiens GN=HECTD1 PE=1 SV=
sp|Q6P587|FAHD1_HUMAN	Acylpyruvase FAHD1 mitochondrial OS=Homo sapiens GN=FAHD1 PE=1 SV=2
sp|Q96M86|DNHD1_HUMAN	Dynein heavy chain domain-containing protein 1 OS=Homo sapiens GN=DNHD1
sp|Q5VT06|CE350_HUMAN	Centrosome-associated protein 350 OS=Homo sapiens GN=CEP350 PE=1 SV=1
sp|P20849|CO9A1_HUMAN	Collagen alpha-1(IX) chain OS=Homo sapiens GN=COL9A1 PE=1 SV=3
sp|Q15485|FCN2_HUMAN	Ficolin-2 OS=Homo sapiens GN=FCN2 PE=1 SV=2
sp|P51531|SMCA2_HUMAN	Probable global transcription activator SNF2L2 OS=Homo sapiens GN=SMARCA
sp|Q9Y5H5|PCDA9_HUMAN	Protocadherin alpha-9 OS=Homo sapiens GN=PCDHA9 PE=2 SV=1
sp|Q6E0U4|DMKN_HUMAN	Dermokine OS=Homo sapiens GN=DMKN PE=1 SV=3
sp|Q9HC84|MUC5B_HUMAN	Mucin-5B OS=Homo sapiens GN=MUC5B PE=1 SV=3
sp|Q9HC35|EMAL4_HUMAN	Echinoderm microtubule-associated protein-like 4 OS=Homo sapiens GN=EML4
sp|Q8WXX7|AUTS2_HUMAN	Autism susceptibility gene 2 protein OS=Homo sapiens GN=AUTS2 PE=1 SV=1
sp|P78509|RELN_HUMAN	Reelin OS=Homo sapiens GN=RELN PE=1 SV=3
A0A075B6R2|A0A075B6R2_HUMAN	Protein IGHV4-4 (Fragment) OS=Homo sapiens GN=IGHV4-4 PE=4 SV=2
sp|P04264|K2C1_HUMAN	Keratin type II cytoskeletal 1 OS=Homo sapiens GN=KRT1 PE=1 SV=6
sp|Q8N3D4|EH1L1_HUMAN	EH domain-binding protein 1-like protein 1 OS=Homo sapiens GN=EHBP1L1 PE=
sp|Q8TD84|DSCL1_HUMAN	Down syndrome cell adhesion molecule-like protein 1 OS=Homo sapiens GN=DS
sp|P07996|TSP1_HUMAN	Thrombospondin-1 OS=Homo sapiens GN=THBS1 PE=1 SV=2
sp|Q63HK5|TSH3_HUMAN	Teashirt homolog 3 OS=Homo sapiens GN=TSHZ3 PE=1 SV=2
sp|Q03692|COAA1_HUMAN	Collagen alpha-1(X) chain OS=Homo sapiens GN=COL10A1 PE=1 SV=2
sp|P46100|ATRX_HUMAN	Transcriptional regulator ATRX OS=Homo sapiens GN=ATRX PE=1 SV=5
sp|Q9UPA5|BSN_HUMAN	Protein bassoon OS=Homo sapiens GN=BSN PE=1 SV=4
sp|O75923|DYSF_HUMAN	Dysferlin OS=Homo sapiens GN=DYSF PE=1 SV=1
sp|P19652|A1AG2_HUMAN	Alpha-1-acid glycoprotein 2 OS=Homo sapiens GN=ORM2 PE=1 SV=2
sp|P04220|MUCB_HUMAN	Ig mu heavy chain disease protein OS=Homo sapiens PE=1 SV=1
sp|Q05BV3|EMAL5_HUMAN	Echinoderm microtubule-associated protein-like 5 OS=Homo sapiens GN=EML5
sp|Q03591|FHR1_HUMAN	Complement factor H-related protein 1 OS=Homo sapiens GN=CFHR1 PE=1 SV
sp|Q96F45|ZN503_HUMAN	Zinc finger protein 503 OS=Homo sapiens GN=ZNF503 PE=1 SV=1
sp|P53804|TTC3_HUMAN	E3 ubiquitin-protein ligase TTC3 OS=Homo sapiens GN=TTC3 PE=1 SV=2
sp|P01623|KV305_HUMAN	Ig kappa chain V-III region WOL OS=Homo sapiens PE=1 SV=1
sp|Q86SH2|ZAR1_HUMAN	Zygote arrest protein 1 OS=Homo sapiens GN=ZAR1 PE=2 SV=1
sp|P08913|ADA2A_HUMAN	Alpha-2A adrenergic receptor OS=Homo sapiens GN=ADRA2A PE=1 SV=3
sp|Q9UHF7|TRPS1_HUMAN	Zinc finger transcription factor Trps1 OS=Homo sapiens GN=TRPS1 PE=1 SV=2
sp|Q15154|PCM1_HUMAN	Pericentriolar material 1 protein OS=Homo sapiens GN=PCM1 PE=1 SV=4
sp|Q7L311|ARMX2_HUMAN	Armadillo repeat-containing X-linked protein 2 OS=Homo sapiens GN=ARMCX2 I
sp|Q8NC51|PAIRB_HUMAN	Plasminogen activator inhibitor 1 RNA-binding protein OS=Homo sapiens GN=SE
sp|O43237|DC1L2_HUMAN	Cytoplasmic dynein 1 light intermediate chain 2 OS=Homo sapiens GN=DYNC1L
sp|O60346|PHLP1_HUMAN	PH domain leucine-rich repeat-containing protein phosphatase 1 OS=Homo sapie
sp|Q9NY99|SNTG2_HUMAN	Gamma-2-syntrophin OS=Homo sapiens GN=SNTG2 PE=1 SV=2
sp|Q9HBB8|CDHR5_HUMAN	Cadherin-related family member 5 OS=Homo sapiens GN=CDHR5 PE=1 SV=3
sp|Q9H987|SYP2L_HUMAN	Synaptopodin 2-like protein OS=Homo sapiens GN=SYNPO2L PE=2 SV=3
sp|P18428|LBP_HUMAN	Lipopolysaccharide-binding protein OS=Homo sapiens GN=LBP PE=1 SV=3
sp|Q92625|ANS1A_HUMAN	Ankyrin repeat and SAM domain-containing protein 1A OS=Homo sapiens GN=A
sp|O43157|PLXB1_HUMAN	Plexin-B1 OS=Homo sapiens GN=PLXNB1 PE=1 SV=3
sp|Q92900|RENT1_HUMAN	Regulator of nonsense transcripts 1 OS=Homo sapiens GN=UPF1 PE=1 SV=2

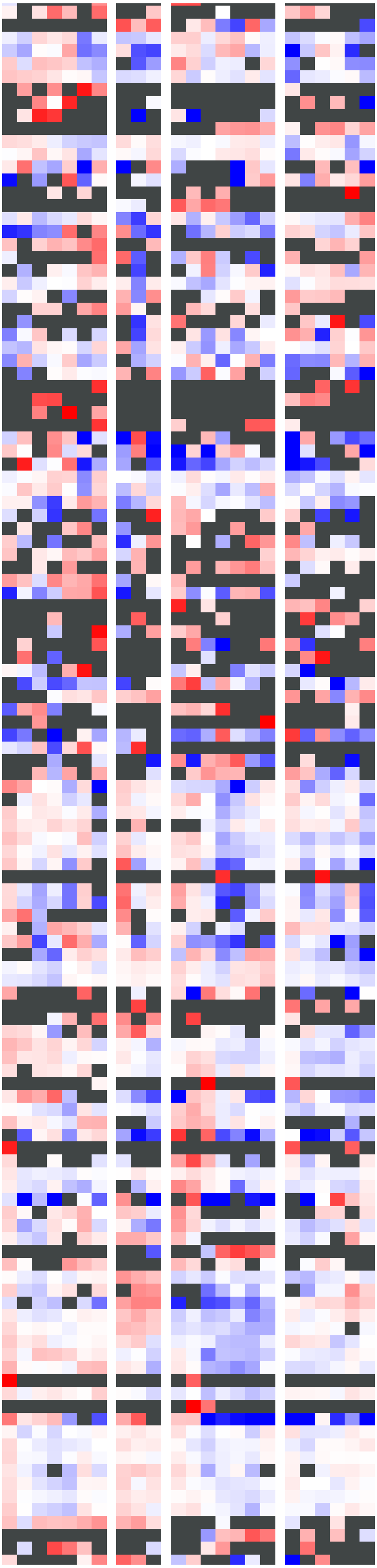

[illegible]
sp|Q9C093|SPEF2_HUMAN Sperm flagellar protein 2 OS=Homo sapiens GN=SPEF2 PE=1 SV=2
sp|P13611|CSPG2_HUMAN Versican core protein OS=Homo sapiens GN=VCAN PE=1 SV=3
sp|P51884|LUM_HUMAN Lumican OS=Homo sapiens GN=LUM PE=1 SV=2
sp|P06311|KV311_HUMAN Ig kappa chain V-III region IARC/BL41 OS=Homo sapiens PE=4 SV=1
sp|O00468|AGRIN_HUMAN Agrin OS=Homo sapiens GN=AGRN PE=1 SV=5
sp|Q8N392|RHG18_HUMAN Rho GTPase-activating protein 18 OS=Homo sapiens GN=ARHGAP18 PE=1 SV
sp|Q96NA8|TSNA1_HUMAN t-SNARE domain-containing protein 1 OS=Homo sapiens GN=TSNARE1 PE=1 S
sp|Q8N7B6|PACRL_HUMAN PACRG-like protein OS=Homo sapiens GN=PACRGL PE=1 SV=2
sp|P28300|LYOX_HUMAN Protein-lysine 6-oxidase OS=Homo sapiens GN=LOX PE=1 SV=2
sp|Q9UP79|ATS8_HUMAN A disintegrin and metalloproteinase with thrombospondin motifs 8 OS=Homo sap
sp|Q9BTC0|DIDO1_HUMAN Death-inducer obliterator 1 OS=Homo sapiens GN=DIDO1 PE=1 SV=5
A0A075B6S4|A0A075B6S4_HUMAN Protein IGKV1D-17 (Fragment) OS=Homo sapiens GN=IGKV1D-17 PE=4 SV=1
sp|Q9UKZ1|CNO11_HUMAN CCR4-NOT transcription complex subunit 11 OS=Homo sapiens GN=CNOT11 PE
A0A0C4DH33|A0A0C4DH33_HUMAN Protein IGHV1-24 (Fragment) OS=Homo sapiens GN=IGHV1-24 PE=1 SV=1
sp|P50213|IDH3A_HUMAN Isocitrate dehydrogenase [NAD] subunit alpha mitochondrial OS=Homo sapiens (
sp|Q01955|CO4A3_HUMAN Collagen alpha-3(IV) chain OS=Homo sapiens GN=COL4A3 PE=1 SV=3
sp|O60341|KDM1A_HUMAN Lysine-specific histone demethylase 1A OS=Homo sapiens GN=KDM1A PE=1 SV
sp|P08637|FCG3A_HUMAN Low affinity immunoglobulin gamma Fc region receptor III-A OS=Homo sapiens G
sp|P04406|G3P_HUMAN Glyceraldehyde-3-phosphate dehydrogenase OS=Homo sapiens GN=GAPDH PE
sp|Q15554|TERF2_HUMAN Telomeric repeat-binding factor 2 OS=Homo sapiens GN=TERF2 PE=1 SV=3
sp|Q96EV2|RBM33_HUMAN RNA-binding protein 33 OS=Homo sapiens GN=RBM33 PE=1 SV=3
sp|P03952|KLKB1_HUMAN Plasma kallikrein OS=Homo sapiens GN=KLKB1 PE=1 SV=1
sp|Q9UHV7|MED13_HUMAN Mediator of RNA polymerase II transcription subunit 13 OS=Homo sapiens GN=M
sp|Q8N1B3|FA58A_HUMAN Cyclin-related protein FAM58A OS=Homo sapiens GN=FAM58A PE=1 SV=2
sp|Q9UHC7|MKRN1_HUMAN E3 ubiquitin-protein ligase makorin-1 OS=Homo sapiens GN=MKRN1 PE=1 SV=
sp|Q9P281|BAHC1_HUMAN BAH and coiled-coil domain-containing protein 1 OS=Homo sapiens GN=BAHCC
sp|Q14956|GPNMB_HUMAN Transmembrane glycoprotein NMB OS=Homo sapiens GN=GPNMB PE=1 SV=2
sp|Q7Z408|CSMD2_HUMAN CUB and sushi domain-containing protein 2 OS=Homo sapiens GN=CSMD2 PE=
sp|Q9Y2T1|AXIN2_HUMAN Axin-2 OS=Homo sapiens GN=AXIN2 PE=1 SV=1
sp|Q9Y6D6|BIG1_HUMAN Brefeldin A-inhibited guanine nucleotide-exchange protein 1 OS=Homo sapiens (
sp|Q8TC56|FA71B_HUMAN Protein FAM71B OS=Homo sapiens GN=FAM71B PE=1 SV=2
sp|Q8TDG4|HELQ_HUMAN Helicase POLQ-like OS=Homo sapiens GN=HELQ PE=1 SV=2
sp|Q9P2D7|DYH1_HUMAN Dynein heavy chain 1 axonemal OS=Homo sapiens GN=DNAH1 PE=2 SV=4
sp|O00555|CAC1A_HUMAN Voltage-dependent P/Q-type calcium channel subunit alpha-1A OS=Homo sapien
sp|O15075|DCLK1_HUMAN Serine/threonine-protein kinase DCLK1 OS=Homo sapiens GN=DCLK1 PE=1 SV
sp|P33981|TTK_HUMAN Dual specificity protein kinase TTK OS=Homo sapiens GN=TTK PE=1 SV=2
sp|P20908|CO5A1_HUMAN Collagen alpha-1(V) chain OS=Homo sapiens GN=COL5A1 PE=1 SV=3
sp|Q96JE7|SC16B_HUMAN Protein transport protein Sec16B OS=Homo sapiens GN=SEC16B PE=1 SV=2
sp|P27918|PROP_HUMAN Properdin OS=Homo sapiens GN=CFP PE=1 SV=2
sp|Q8NFC6|BD1L1_HUMAN Biorientation of chromosomes in cell division protein 1-like 1 OS=Homo sapiens (
sp|O43918|AIRE_HUMAN Autoimmune regulator OS=Homo sapiens GN=AIRE PE=1 SV=1
sp|Q9H9Y2|RPF1_HUMAN Ribosome production factor 1 OS=Homo sapiens GN=RPF1 PE=1 SV=2
sp|Q13423|NNTM_HUMAN NAD(P) transhydrogenase mitochondrial OS=Homo sapiens GN=NNT PE=1 SV=
sp|Q15582|BGH3_HUMAN Transforming growth factor-beta-induced protein ig-h3 OS=Homo sapiens GN=TG
sp|A8MYA2|CX049_HUMAN Uncharacterized protein CXorf49 OS=Homo sapiens GN=CXorf49 PE=2 SV=3
sp|Q86UR5|RIMS1_HUMAN Regulating synaptic membrane exocytosis protein 1 OS=Homo sapiens GN=RIM
sp|Q96NY7|CLIC6_HUMAN Chloride intracellular channel protein 6 OS=Homo sapiens GN=CLIC6 PE=2 SV=
sp|Q9Y5Z7|HCFC2_HUMAN Host cell factor 2 OS=Homo sapiens GN=HCFC2 PE=1 SV=1
sp|Q8IX21|SLF2_HUMAN SMC5-SMC6 complex localization factor protein 2 OS=Homo sapiens GN=SLF2
sp|Q10571|MN1_HUMAN Transcriptional activator MN1 OS=Homo sapiens GN=MN1 PE=1 SV=3
sp|Q9BWU1|CDK19_HUMAN Cyclin-dependent kinase 19 OS=Homo sapiens GN=CDK19 PE=1 SV=1
sp|Q9NR82|KCNQ5_HUMAN Potassium voltage-gated channel subfamily KQT member 5 OS=Homo sapiens G
sp|Q9P2P1|NYNRI_HUMAN Protein NYNRIN OS=Homo sapiens GN=NYNRIN PE=2 SV=3
sp|P17936|IBP3_HUMAN Insulin-like growth factor-binding protein 3 OS=Homo sapiens GN=IGFBP3 PE=1
sp|B1AJZ9|FHAD1_HUMAN Forkhead-associated domain-containing protein 1 OS=Homo sapiens GN=FHAD
sp|Q96QC0|PP1RA_HUMAN Serine/threonine-protein phosphatase 1 regulatory subunit 10 OS=Homo sapiens
sp|O95071|UBR5_HUMAN E3 ubiquitin-protein ligase UBR5 OS=Homo sapiens GN=UBR5 PE=1 SV=2
sp|Q9ULD9|ZN608_HUMAN Zinc finger protein 608 OS=Homo sapiens GN=ZNF608 PE=1 SV=4
sp|Q9BQ89|F110A_HUMAN Protein FAM110A OS=Homo sapiens GN=FAM110A PE=1 SV=1
sp|Q96MM6|HS12B_HUMAN Heat shock 70 kDa protein 12B OS=Homo sapiens GN=HSPA12B PE=1 SV=2
sp|P78362|SRPK2_HUMAN SRSF protein kinase 2 OS=Homo sapiens GN=SRPK2 PE=1 SV=3
sp|P10275|ANDR_HUMAN Androgen receptor OS=Homo sapiens GN=AR PE=1 SV=3
sp|Q5VST9|OBSCN_HUMAN Obscurin OS=Homo sapiens GN=OBSCN PE=1 SV=3
sp|P08185|CBG_HUMAN Corticosteroid-binding globulin OS=Homo sapiens GN=SERPINA6 PE=1 SV=1
sp|P02652|APOA2_HUMAN Apolipoprotein A-II OS=Homo sapiens GN=APOA2 PE=1 SV=1
sp|P05155|IC1_HUMAN Plasma protease C1 inhibitor OS=Homo sapiens GN=SERPING1 PE=1 SV=2
sp|O14511|NRG2_HUMAN Pro-neuregulin-2 membrane-bound isoform OS=Homo sapiens GN=NRG2 PE=1
sp|Q13425|SNTB2_HUMAN Beta-2-syntrophin OS=Homo sapiens GN=SNTB2 PE=1 SV=1
sp|Q5T1R4|ZEP3_HUMAN Transcription factor HIVEP3 OS=Homo sapiens GN=HIVEP3 PE=2 SV=1
sp|O15047|SET1A_HUMAN Histone-lysine N-methyltransferase SETD1A OS=Homo sapiens GN=SETD1A PE
sp|Q2KJY2|KI26B_HUMAN Kinesin-like protein KIF26B OS=Homo sapiens GN=KIF26B PE=2 SV=1
sp|P12259|FA5_HUMAN Coagulation factor V OS=Homo sapiens GN=F5 PE=1 SV=4
sp|Q9Y2F5|ICE1_HUMAN Little elongation complex subunit 1 OS=Homo sapiens GN=ICE1 PE=1 SV=5
sp|Q99490|AGAP2_HUMAN Arf-GAP with GTPase ANK repeat and PH domain-containing protein 2 OS=Hom
sp|P02647|APOA1_HUMAN Apolipoprotein A-I OS=Homo sapiens GN=APOA1 PE=1 SV=1
sp|Q96MC4|C295L_HUMAN CEP295 N-terminal-like protein OS=Homo sapiens GN=CEP295NL PE=2 SV=1
sp|Q9UQ35|SRRM2_HUMAN Serine/arginine repetitive matrix protein 2 OS=Homo sapiens GN=SRRM2 PE=1
sp|P02655|APOC2_HUMAN Apolipoprotein C-II OS=Homo sapiens GN=APOC2 PE=1 SV=1
sp|Q494U1|PKHN1_HUMAN Pleckstrin homology domain-containing family N member 1 OS=Homo sapiens G
sp|P48382|RFX5_HUMAN DNA-binding protein RFX5 OS=Homo sapiens GN=RFX5 PE=1 SV=1
sp|P07360|CO8G_HUMAN Complement component C8 gamma chain OS=Homo sapiens GN=C8G PE=1 SV
sp|P15169|CBPN_HUMAN Carboxypeptidase N catalytic chain OS=Homo sapiens GN=CPN1 PE=1 SV=1
sp|Q86XX4|FRAS1_HUMAN Extracellular matrix protein FRAS1 OS=Homo sapiens GN=FRAS1 PE=1 SV=2
sp|Q9C0K0|BC11B_HUMAN B-cell lymphoma/leukemia 11B OS=Homo sapiens GN=BCL11B PE=1 SV=1
Q9P1D2|Q9P1D2_HUMAN PRO2472 OS=Homo sapiens PE=2 SV=1
sp|P06396|GELS_HUMAN Gelsolin OS=Homo sapiens GN=GSN PE=1 SV=1
sp|P01621|KV303_HUMAN Ig kappa chain V-III region NG9 (Fragment) OS=Homo sapiens PE=2 SV=1
sp|Q9P217|ZSWM5_HUMAN Zinc finger SWIM domain-containing protein 5 OS=Homo sapiens GN=ZSWIM5 F
sp|Q16799|RTN1_HUMAN Reticulon-1 OS=Homo sapiens GN=RTN1 PE=1 SV=1
sp|Q69YQ0|CYTSA_HUMAN Cytospin-A OS=Homo sapiens GN=SPECC1L PE=1 SV=2
sp|Q99714|HCD2_HUMAN 3-hydroxyacyl-CoA dehydrogenase type-2 OS=Homo sapiens GN=HSD17B10 PE
sp|Q3SYB3|FX4L6_HUMAN Forkhead box protein D4-like 6 OS=Homo sapiens GN=FOXD4L6 PE=1 SV=2
sp|P20742|PZP_HUMAN Pregnancy zone protein OS=Homo sapiens GN=PZP PE=1 SV=4
sp|Q9UM73|ALK_HUMAN ALK tyrosine kinase receptor OS=Homo sapiens GN=ALK PE=1 SV=3
M0QZD8|M0QZD8_HUMAN Uncharacterized protein OS=Homo sapiens PE=1 SV=2
sp|Q9NUA8|ZBT40_HUMAN Zinc finger and BTB domain-containing protein 40 OS=Homo sapiens GN=ZBTB4
sp|Q86TB3|ALPK2_HUMAN Alpha-protein kinase 2 OS=Homo sapiens GN=ALPK2 PE=2 SV=3
sp|O60299|LZTS3_HUMAN Leucine zipper putative tumor suppressor 3 OS=Homo sapiens GN=LZTS3 PE=2
sp|P01877|IGHA2_HUMAN Ig alpha-2 chain C region OS=Homo sapiens GN=IGHA2 PE=1 SV=3
sp|Q04759|KPCT_HUMAN Protein kinase C theta type OS=Homo sapiens GN=PRKCQ PE=1 SV=3
sp|P01880|IGHD_HUMAN Ig delta chain C region OS=Homo sapiens GN=IGHD PE=1 SV=2
sp|O14791|APOL1_HUMAN Apolipoprotein L1 OS=Homo sapiens GN=APOL1 PE=1 SV=5
A0A075B6I4|A0A075B6I4_HUMAN Protein IGLV10-54 (Fragment) OS=Homo sapiens GN=IGLV10-54 PE=1 SV=1
sp|Q86VF2|IGFN1_HUMAN Immunoglobulin-like and fibronectin type III domain-containing protein 1 OS=Hom
sp|P01700|LV102_HUMAN Ig lambda chain V-I region HA OS=Homo sapiens PE=1 SV=1
sp|P27169|PON1_HUMAN Serum paraoxonase/arylesterase 1 OS=Homo sapiens GN=PON1 PE=1 SV=3
sp|Q92995|UBP13_HUMAN Ubiquitin carboxyl-terminal hydrolase 13 OS=Homo sapiens GN=USP13 PE=1 SV
sp|P12107|COBA1_HUMAN Collagen alpha-1(XI) chain OS=Homo sapiens GN=COL11A1 PE=1 SV=4
sp|Q9UMN6|KMT2B_HUMAN Histone-lysine N-methyltransferase 2B OS=Homo sapiens GN=KMT2B PE=1 SV
sp|Q99607|ELF4_HUMAN ETS-related transcription factor Elf-4 OS=Homo sapiens GN=ELF4 PE=1 SV=1
sp|A6NC98|CC88B_HUMAN Coiled-coil domain-containing protein 88B OS=Homo sapiens GN=CCDC88B PE
sp|P02452|CO1A1_HUMAN Collagen alpha-1(I) chain OS=Homo sapiens GN=COL1A1 PE=1 SV=5
sp|P02743|SAMP_HUMAN Serum amyloid P-component OS=Homo sapiens GN=APCS PE=1 SV=2
sp|O94901|SUN1_HUMAN SUN domain-containing protein 1 OS=Homo sapiens GN=SUN1 PE=1 SV=3
sp|Q8WXH0|SYNE2_HUMAN Nesprin-2 OS=Homo sapiens GN=SYNE2 PE=1 SV=3
sp|P05997|CO5A2_HUMAN Collagen alpha-2(V) chain OS=Homo sapiens GN=COL5A2 PE=1 SV=3
sp|Q9C0F0|ASXL3_HUMAN Putative Polycomb group protein ASXL3 OS=Homo sapiens GN=ASXL3 PE=2 SV
A0A0C4DH36|A0A0C4DH36_HUMAN Protein IGHV3-38 (Fragment) OS=Homo sapiens GN=IGHV3-38 PE=1 SV=1
sp|Q9Y3Q4|HCN4_HUMAN Potassium/sodium hyperpolarization-activated cyclic nucleotide-gated channel 4 (
sp|P17655|CAN2_HUMAN Calpain-2 catalytic subunit OS=Homo sapiens GN=CAPN2 PE=1 SV=6
[illegible]

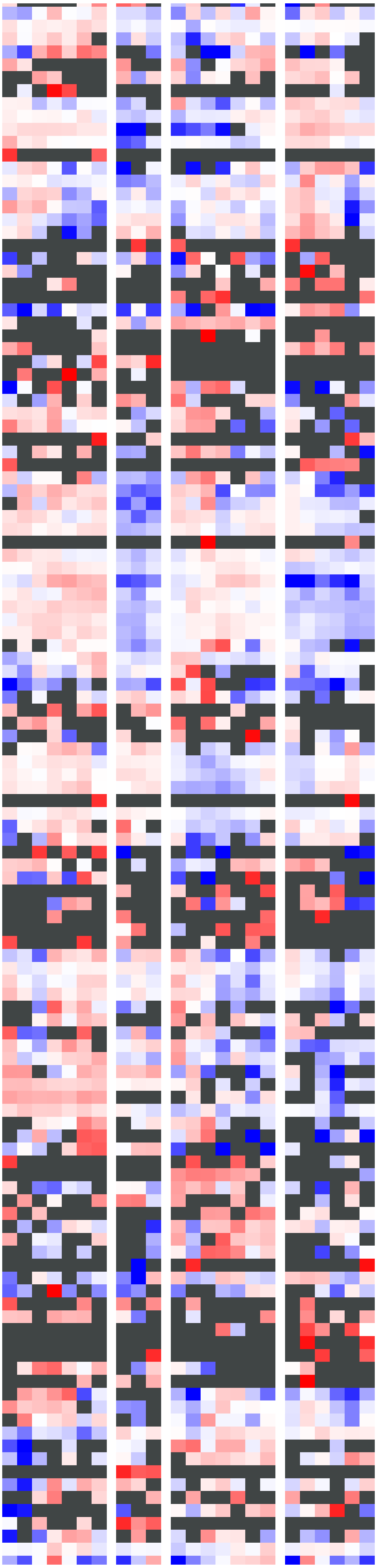

[illegible]
sp|Q8WXS5|CCG8_HUMAN Voltage-dependent calcium channel gamma-8 subunit OS=Homo sapiens GN=C
sp|Q5TH69|BIG3_HUMAN Brefeldin A-inhibited guanine nucleotide-exchange protein 3 OS=Homo sapiens
sp|P40939|ECHA_HUMAN Trifunctional enzyme subunit alpha mitochondrial OS=Homo sapiens GN=HADH
sp|Q86UE4|LYRIC_HUMAN Protein LYRIC OS=Homo sapiens GN=MTDH PE=1 SV=2
sp|Q86U17|SPA11_HUMAN Serpin A11 OS=Homo sapiens GN=SERPINA11 PE=2 SV=2
sp|Q6IN84|MRM1_HUMAN rRNA methyltransferase 1 mitochondrial OS=Homo sapiens GN=MRM1 PE=1 SV
sp|Q8NCD3|HJURP_HUMAN Holliday junction recognition protein OS=Homo sapiens GN=HJURP PE=1 SV=2
sp|P0C0L5|CO4B_HUMAN Complement C4-B OS=Homo sapiens GN=C4B PE=1 SV=2
sp|Q96IY4|CBPB2_HUMAN Carboxypeptidase B2 OS=Homo sapiens GN=CPB2 PE=1 SV=2
sp|P36980|FHR2_HUMAN Complement factor H-related protein 2 OS=Homo sapiens GN=CFHR2 PE=1 SV
sp|Q8WWN8|ARAP3_HUMAN Arf-GAP with Rho-GAP domain ANK repeat and PH domain-containing protein 3
sp|Q9NYQ6|CELR1_HUMAN Cadherin EGF LAG seven-pass G-type receptor 1 OS=Homo sapiens GN=CELS
sp|O60902|SHOX2_HUMAN Short stature homeobox protein 2 OS=Homo sapiens GN=SHOX2 PE=2 SV=4
sp|Q9UQP3|TENN_HUMAN Tenascin-N OS=Homo sapiens GN=TNN PE=1 SV=2
sp|Q96JQ0|PCD16_HUMAN Protocadherin-16 OS=Homo sapiens GN=DCHS1 PE=1 SV=1
sp|Q9BXI3|5NT1A_HUMAN Cytosolic 5'-nucleotidase 1A OS=Homo sapiens GN=NT5C1A PE=2 SV=1
sp|Q09666|AHNK_HUMAN Neuroblast differentiation-associated protein AHNAK OS=Homo sapiens GN=AH
sp|P55036|PSMD4_HUMAN 26S proteasome non-ATPase regulatory subunit 4 OS=Homo sapiens GN=PSM
sp|Q6ZMY3|SPOC1_HUMAN SPOC domain-containing protein 1 OS=Homo sapiens GN=SPOCD1 PE=2 SV=
sp|P06331|HV434_HUMAN Immunoglobulin heavy variable 4-34 OS=Homo sapiens GN=IGHV4-34 PE=1 SV
sp|P47897|SYQ_HUMAN Glutamine--tRNA ligase OS=Homo sapiens GN=QARS PE=1 SV=1
sp|P22681|CBL_HUMAN E3 ubiquitin-protein ligase CBL OS=Homo sapiens GN=CBL PE=1 SV=2
sp|Q711Q0|CJ071_HUMAN Uncharacterized protein C10orf71 OS=Homo sapiens GN=C10orf71 PE=1 SV=2
sp|Q5TID7|CC181_HUMAN Coiled-coil domain-containing protein 181 OS=Homo sapiens GN=CCDC181 PE
sp|P01594|KV102_HUMAN Ig kappa chain V-I region AU OS=Homo sapiens PE=1 SV=1
sp|O95390|GDF11_HUMAN Growth/differentiation factor 11 OS=Homo sapiens GN=GDF11 PE=1 SV=1
sp|P04431|KV123_HUMAN Ig kappa chain V-I region Walker OS=Homo sapiens PE=1 SV=1
S4R3C0|S4R3C0_HUMAN Uncharacterized protein (Fragment) OS=Homo sapiens PE=4 SV=1
sp|Q9H5I5|PIEZ2_HUMAN Piezo-type mechanosensitive ion channel component 2 OS=Homo sapiens GN=
sp|O14727|APAF_HUMAN Apoptotic protease-activating factor 1 OS=Homo sapiens GN=APAF1 PE=1 SV=
sp|Q15697|ZN174_HUMAN Zinc finger protein 174 OS=Homo sapiens GN=ZNF174 PE=1 SV=1
A0A087WSX0|A0A087WSX0_HUMAN Protein IGLV5-45 (Fragment) OS=Homo sapiens GN=IGLV5-45 PE=1 SV=1
sp|P06702|S10A9_HUMAN Protein S100-A9 OS=Homo sapiens GN=S100A9 PE=1 SV=1
sp|P46379|BAG6_HUMAN Large proline-rich protein BAG6 OS=Homo sapiens GN=BAG6 PE=1 SV=2
sp|P08571|CD14_HUMAN Monocyte differentiation antigen CD14 OS=Homo sapiens GN=CD14 PE=1 SV=
A0A0B4J1V8|A0A0B4J1V8_HUMAN HCG2039996 OS=Homo sapiens GN=PPAN-P2RY11 PE=3 SV=1
sp|Q9P219|DAPLE_HUMAN Protein Daple OS=Homo sapiens GN=CCDC88C PE=1 SV=3
sp|Q14789|GOGB1_HUMAN Golgin subfamily B member 1 OS=Homo sapiens GN=GOLGB1 PE=1 SV=2
sp|Q66K41|Z385C_HUMAN Zinc finger protein 385C OS=Homo sapiens GN=ZNF385C PE=1 SV=2
sp|P21817|RYR1_HUMAN Ryanodine receptor 1 OS=Homo sapiens GN=RYR1 PE=1 SV=3
sp|P01024|CO3_HUMAN Complement C3 OS=Homo sapiens GN=C3 PE=1 SV=2
sp|Q96KN2|CNDP1_HUMAN Beta-Ala-His dipeptidase OS=Homo sapiens GN=CNDP1 PE=1 SV=4
sp|O60673|DPOLZ_HUMAN DNA polymerase zeta catalytic subunit OS=Homo sapiens GN=REV3L PE=1 SV
sp|P01591|IGJ_HUMAN Immunoglobulin J chain OS=Homo sapiens GN=JCHAIN PE=1 SV=4
sp|Q8IZQ1|WDFY3_HUMAN WD repeat and FYVE domain-containing protein 3 OS=Homo sapiens GN=WDF
sp|P02745|C1QA_HUMAN Complement C1q subcomponent subunit A OS=Homo sapiens GN=C1QA PE=1
sp|P02747|C1QC_HUMAN Complement C1q subcomponent subunit C OS=Homo sapiens GN=C1QC PE=1
sp|P02746|C1QB_HUMAN Complement C1q subcomponent subunit B OS=Homo sapiens GN=C1QB PE=1
sp|Q9NZR2|LRP1B_HUMAN Low-density lipoprotein receptor-related protein 1B OS=Homo sapiens GN=LRP1
sp|C9JLR9|CK095_HUMAN Uncharacterized protein C11orf95 OS=Homo sapiens GN=C11orf95 PE=2 SV=1
sp|Q9Y4H2|IRS2_HUMAN Insulin receptor substrate 2 OS=Homo sapiens GN=IRS2 PE=1 SV=2
sp|P11177|ODPB_HUMAN Pyruvate dehydrogenase E1 component subunit beta mitochondrial OS=Homo s
sp|Q96C57|CL043_HUMAN Uncharacterized protein C12orf43 OS=Homo sapiens GN=C12orf43 PE=1 SV=2
sp|Q8TDY2|RBCC1_HUMAN RB1-inducible coiled-coil protein 1 OS=Homo sapiens GN=RB1CC1 PE=1 SV=3
sp|O75069|TMCC2_HUMAN Transmembrane and coiled-coil domains protein 2 OS=Homo sapiens GN=TMC
sp|P01743|HV146_HUMAN Immunoglobulin heavy variable 1-46 OS=Homo sapiens GN=IGHV1-46 PE=1 SV
sp|Q92908|GATA6_HUMAN Transcription factor GATA-6 OS=Homo sapiens GN=GATA6 PE=1 SV=2
sp|P03951|FA11_HUMAN Coagulation factor XI OS=Homo sapiens GN=F11 PE=1 SV=1
sp|P19827|ITIH1_HUMAN Inter-alpha-trypsin inhibitor heavy chain H1 OS=Homo sapiens GN=ITIH1 PE=1
sp|P10909|CLUS_HUMAN Clusterin OS=Homo sapiens GN=CLU PE=1 SV=1
sp|P05546|HEP2_HUMAN Heparin cofactor 2 OS=Homo sapiens GN=SERPIND1 PE=1 SV=3
sp|P0CG38|POTEI_HUMAN POTE ankyrin domain family member I OS=Homo sapiens GN=POTEI PE=3 SV
sp|P01042|KNG1_HUMAN Kininogen-1 OS=Homo sapiens GN=KNG1 PE=1 SV=2
sp|Q02880|TOP2B_HUMAN DNA topoisomerase 2-beta OS=Homo sapiens GN=TOP2B PE=1 SV=3
sp|Q5JSZ5|PRC2B_HUMAN Protein PRRC2B OS=Homo sapiens GN=PRRC2B PE=1 SV=2
sp|A5PLN7|F149A_HUMAN Protein FAM149A OS=Homo sapiens GN=FAM149A PE=2 SV=2
sp|P00740|FA9_HUMAN Coagulation factor IX OS=Homo sapiens GN=F9 PE=1 SV=2
sp|Q2UY09|COSA1_HUMAN Collagen alpha-1(XXVIII) chain OS=Homo sapiens GN=COL28A1 PE=2 SV=2
sp|Q9UKI8|TLK1_HUMAN Serine/threonine-protein kinase tousled-like 1 OS=Homo sapiens GN=TLK1 PE=
sp|Q9HBQ8|GGA2B_HUMAN Putative golgin subfamily A member 2B OS=Homo sapiens GN=GOLGA2P5 PE=
sp|Q15911|ZFHX3_HUMAN Zinc finger homeobox protein 3 OS=Homo sapiens GN=ZFHX3 PE=1 SV=2
sp|Q9UPR0|PLCL2_HUMAN Inactive phospholipase C-like protein 2 OS=Homo sapiens GN=PLCL2 PE=1 SV
sp|Q9BWP8|COL11_HUMAN Collectin-11 OS=Homo sapiens GN=COLEC11 PE=1 SV=1
sp|P35908|K22E_HUMAN Keratin type II cytoskeletal 2 epidermal OS=Homo sapiens GN=KRT2 PE=1 SV=
sp|P02763|A1AG1_HUMAN Alpha-1-acid glycoprotein 1 OS=Homo sapiens GN=ORM1 PE=1 SV=1
sp|Q96KQ4|ASPP1_HUMAN Apoptosis-stimulating of p53 protein 1 OS=Homo sapiens GN=PPP1R13B PE=1
sp|Q9P2E9|RRBP1_HUMAN Ribosome-binding protein 1 OS=Homo sapiens GN=RRBP1 PE=1 SV=4
sp|Q9Y2K3|MYH15_HUMAN Myosin-15 OS=Homo sapiens GN=MYH15 PE=1 SV=5
sp|H7C350|CC188_HUMAN Coiled-coil domain-containing protein 188 OS=Homo sapiens GN=CCDC188 PE
sp|O60548|FOXD2_HUMAN Forkhead box protein D2 OS=Homo sapiens GN=FOXD2 PE=1 SV=2
sp|Q9Y5H3|PCDGA_HUMAN Protocadherin gamma-A10 OS=Homo sapiens GN=PCDHGA10 PE=2 SV=1
sp|Q6EKJ0|GTD2B_HUMAN General transcription factor II-I repeat domain-containing protein 2B OS=Homo s
A0A0B4J1V1|A0A0B4J1V1_HUMAN Protein IGHV3-21 (Fragment) OS=Homo sapiens GN=IGHV3-21 PE=1 SV=1
sp|Q6GQQ9|OTU7B_HUMAN OTU domain-containing protein 7B OS=Homo sapiens GN=OTUD7B PE=1 SV=1
sp|O60502|OGA_HUMAN Protein O-GlcNAcase OS=Homo sapiens GN=MGEA5 PE=1 SV=2
sp|Q01668|CAC1D_HUMAN Voltage-dependent L-type calcium channel subunit alpha-1D OS=Homo sapiens
sp|P11310|ACADM_HUMAN Medium-chain specific acyl-CoA dehydrogenase mitochondrial OS=Homo sapien
sp|Q7Z3S7|CA2D4_HUMAN Voltage-dependent calcium channel subunit alpha-2/delta-4 OS=Homo sapiens
sp|Q9BYK8|HELZ2_HUMAN Helicase with zinc finger domain 2 OS=Homo sapiens GN=HELZ2 PE=1 SV=6
sp|O60285|NUAK1_HUMAN NUAK family SNF1-like kinase 1 OS=Homo sapiens GN=NUAK1 PE=1 SV=1
sp|P49327|FAS_HUMAN Fatty acid synthase OS=Homo sapiens GN=FASN PE=1 SV=3
sp|E9PAV3|NACAM_HUMAN Nascent polypeptide-associated complex subunit alpha muscle-specific form OS=
sp|Q5T0T0|MARH8_HUMAN E3 ubiquitin-protein ligase MARCH8 OS=Homo sapiens GN=MARCH8 PE=1 SV
sp|Q14995|NR1D2_HUMAN Nuclear receptor subfamily 1 group D member 2 OS=Homo sapiens GN=NR1D2
sp|P53597|SUCA_HUMAN Succinate--CoA ligase [ADP/GDP-forming] subunit alpha mitochondrial OS=Hom
sp|Q9BWT6|MND1_HUMAN Meiotic nuclear division protein 1 homolog OS=Homo sapiens GN=MND1 PE=1
sp|Q2TAZ0|ATG2A_HUMAN Autophagy-related protein 2 homolog A OS=Homo sapiens GN=ATG2A PE=1 SV
sp|Q96GX5|GWL_HUMAN Serine/threonine-protein kinase greatwall OS=Homo sapiens GN=MASTL PE=1
sp|P23142|FBLN1_HUMAN Fibulin-1 OS=Homo sapiens GN=FBLN1 PE=1 SV=4
sp|P02042|HBD_HUMAN Hemoglobin subunit delta OS=Homo sapiens GN=HBD PE=1 SV=2
sp|Q92973|TNPO1_HUMAN Transportin-1 OS=Homo sapiens GN=TNPO1 PE=1 SV=2
sp|O14828|SCAM3_HUMAN Secretory carrier-associated membrane protein 3 OS=Homo sapiens GN=SCAM
sp|Q13905|RPGF1_HUMAN Rap guanine nucleotide exchange factor 1 OS=Homo sapiens GN=RAPGEF1 PE
sp|Q96SZ4|ZSC10_HUMAN Zinc finger and SCAN domain-containing protein 10 OS=Homo sapiens GN=ZSC
sp|Q8WYB5|KAT6B_HUMAN Histone acetyltransferase KAT6B OS=Homo sapiens GN=KAT6B PE=1 SV=3
sp|Q9Y5I4|PCDC2_HUMAN Protocadherin alpha-C2 OS=Homo sapiens GN=PCDHAC2 PE=2 SV=1
sp|P27816|MAP4_HUMAN Microtubule-associated protein 4 OS=Homo sapiens GN=MAP4 PE=1 SV=3
sp|Q6PKG0|LARP1_HUMAN La-related protein 1 OS=Homo sapiens GN=LARP1 PE=1 SV=2
sp|Q13094|LCP2_HUMAN Lymphocyte cytosolic protein 2 OS=Homo sapiens GN=LCP2 PE=1 SV=1
sp|Q6P158|DHX57_HUMAN Putative ATP-dependent RNA helicase DHX57 OS=Homo sapiens GN=DHX57 P
sp|P22792|CPN2_HUMAN Carboxypeptidase N subunit 2 OS=Homo sapiens GN=CPN2 PE=1 SV=3
sp|P29474|NOS3_HUMAN Nitric oxide synthase endothelial OS=Homo sapiens GN=NOS3 PE=1 SV=3
sp|Q12888|TP53B_HUMAN Tumor suppressor p53-binding protein 1 OS=Homo sapiens GN=TP53BP1 PE=1
sp|Q6AZY7|SCAR3_HUMAN Scavenger receptor class A member 3 OS=Homo sapiens GN=SCARA3 PE=1 S
sp|Q9Y2L9|LRCH1_HUMAN Leucine-rich repeat and calponin homology domain-containing protein 1 OS=Hom
sp|Q05469|LIPS_HUMAN Hormone-sensitive lipase OS=Homo sapiens GN=LIPE PE=1 SV=4
sp|Q15424|SAFB1_HUMAN Scaffold attachment factor B1 OS=Homo sapiens GN=SAFB PE=1 SV=4
sp|O43639|NCK2_HUMAN Cytoplasmic protein NCK2 OS=Homo sapiens GN=NCK2 PE=1 SV=2
sp|Q15814|TBCC_HUMAN Tubulin-specific chaperone C OS=Homo sapiens GN=TBCC PE=1 SV=2
sp|P02649|APOE_HUMAN Apolipoprotein E OS=Homo sapiens GN=APOE PE=1 SV=1
[illegible]

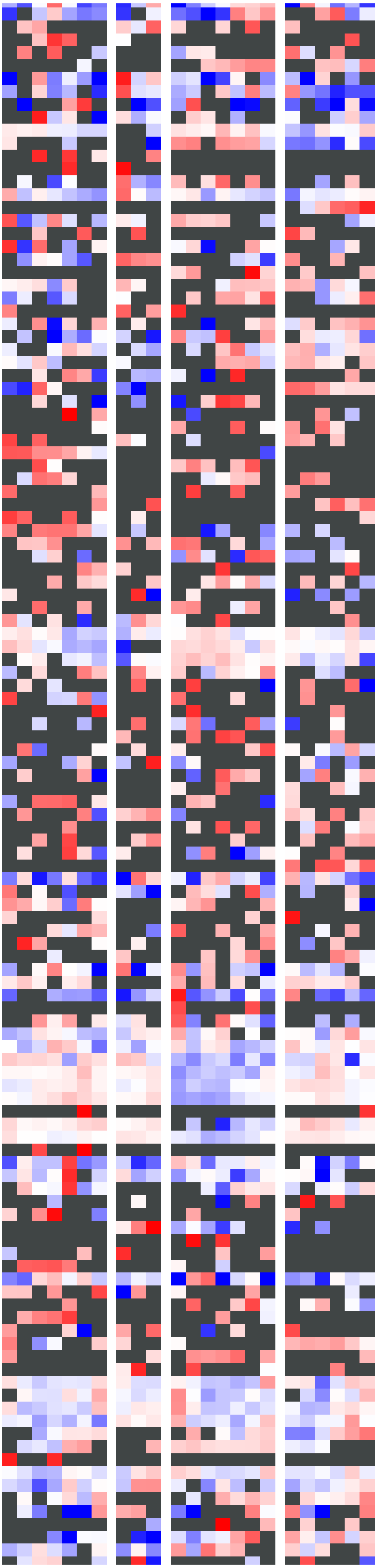

sp|Q92786|RREB1_HUMAN Ras-responsive element-binding protein 1 OS=Homo sapiens GN=RREB1 PE=1
sp|Q9P2S6|ANKY1_HUMAN Ankyrin repeat and MYND domain-containing protein 1 OS=Homo sapiens GN=A
sp|Q8IVT2|MISP_HUMAN Mitotic interactor and substrate of PLK1 OS=Homo sapiens GN=MISP PE=1 SV=
sp|Q8WZ75|ROBO4_HUMAN Roundabout homolog 4 OS=Homo sapiens GN=ROBO4 PE=1 SV=1
sp|Q16512|PKN1_HUMAN Serine/threonine-protein kinase N1 OS=Homo sapiens GN=PKN1 PE=1 SV=2
sp|Q8N9H9|CA127_HUMAN Uncharacterized protein C1orf127 OS=Homo sapiens GN=C1orf127 PE=2 SV=2
sp|Q8NEY1|NAV1_HUMAN Neuron navigator 1 OS=Homo sapiens GN=NAV1 PE=1 SV=2
sp|P49792|RBP2_HUMAN E3 SUMO-protein ligase RanBP2 OS=Homo sapiens GN=RANBP2 PE=1 SV=2
sp|A8K0R7|ZN839_HUMAN Zinc finger protein 839 OS=Homo sapiens GN=ZNF839 PE=2 SV=1
sp|Q9UPV0|CE164_HUMAN Centrosomal protein of 164 kDa OS=Homo sapiens GN=CEP164 PE=1 SV=3
sp|Q13103|SPP24_HUMAN Secreted phosphoprotein 24 OS=Homo sapiens GN=SPP2 PE=1 SV=1
sp|Q9Y600|CSAD_HUMAN Cysteine sulfinic acid decarboxylase OS=Homo sapiens GN=CSAD PE=1 SV=2
sp|A0M8Q6|LAC7_HUMAN Ig lambda-7 chain C region OS=Homo sapiens GN=IGLC7 PE=4 SV=2
sp|P01814|HV270_HUMAN Immunoglobulin heavy variable 2-70 OS=Homo sapiens GN=IGHV2-70 PE=1 SV
sp|Q24JP5|T132A_HUMAN Transmembrane protein 132A OS=Homo sapiens GN=TMEM132A PE=1 SV=1
sp|Q7Z434|MAVS_HUMAN Mitochondrial antiviral-signaling protein OS=Homo sapiens GN=MAVS PE=1 SV=
A0A075B7D0|A0A075B7D0_HUMAN Protein IGHV1OR15-1 (Fragment) OS=Homo sapiens GN=IGHV1OR15-1 PE=1
sp|O60741|HCN1_HUMAN Potassium/sodium hyperpolarization-activated cyclic nucleotide-gated channel 1
sp|Q9HCK8|CHD8_HUMAN Chromodomain-helicase-DNA-binding protein 8 OS=Homo sapiens GN=CHD8 P
sp|Q9BV73|CP250_HUMAN Centrosome-associated protein CEP250 OS=Homo sapiens GN=CEP250 PE=1
sp|P80362|KV125_HUMAN Ig kappa chain V-I region WAT OS=Homo sapiens PE=1 SV=1
sp|Q9BYT3|STK33_HUMAN Serine/threonine-protein kinase 33 OS=Homo sapiens GN=STK33 PE=1 SV=1
sp|P53814|SMTN_HUMAN Smoothelin OS=Homo sapiens GN=SMTN PE=1 SV=7
sp|O75369|FLNB_HUMAN Filamin-B OS=Homo sapiens GN=FLNB PE=1 SV=2
sp|P58397|ATS12_HUMAN A disintegrin and metalloproteinase with thrombospondin motifs 12 OS=Homo sa
sp|Q15131|CDK10_HUMAN Cyclin-dependent kinase 10 OS=Homo sapiens GN=CDK10 PE=1 SV=1
sp|Q9UFH2|DYH17_HUMAN Dynein heavy chain 17 axonemal OS=Homo sapiens GN=DNAH17 PE=1 SV=2
sp|Q8TEJ3|SH3R3_HUMAN SH3 domain-containing RING finger protein 3 OS=Homo sapiens GN=SH3RF3 P
sp|O15015|ZN646_HUMAN Zinc finger protein 646 OS=Homo sapiens GN=ZNF646 PE=1 SV=1
sp|Q96J94|PIWL1_HUMAN Piwi-like protein 1 OS=Homo sapiens GN=PIWIL1 PE=1 SV=1
sp|Q02985|FHR3_HUMAN Complement factor H-related protein 3 OS=Homo sapiens GN=CFHR3 PE=1 SV
sp|Q86UQ4|ABCAD_HUMAN ATP-binding cassette sub-family A member 13 OS=Homo sapiens GN=ABCA13
sp|Q2WGJ9|FR1L6_HUMAN Fer-1-like protein 6 OS=Homo sapiens GN=FER1L6 PE=2 SV=2
sp|Q9P2M7|CING_HUMAN Cingulin OS=Homo sapiens GN=CGN PE=1 SV=2
sp|Q6WKZ4|RFIP1_HUMAN Rab11 family-interacting protein 1 OS=Homo sapiens GN=RAB11FIP1 PE=1 SV=
sp|P01712|LV209_HUMAN Ig lambda chain V-II region WIN OS=Homo sapiens PE=1 SV=1
sp|Q8TED0|UTP15_HUMAN U3 small nucleolar RNA-associated protein 15 homolog OS=Homo sapiens GN=
sp|Q2M3G0|ABCB5_HUMAN ATP-binding cassette sub-family B member 5 OS=Homo sapiens GN=ABCB5 PE
sp|P07954|FUMH_HUMAN Fumarate hydratase mitochondrial OS=Homo sapiens GN=FH PE=1 SV=3
sp|O75534|CSDE1_HUMAN Cold shock domain-containing protein E1 OS=Homo sapiens GN=CSDE1 PE=1
sp|O95171|SCEL_HUMAN Sciellin OS=Homo sapiens GN=SCEL PE=1 SV=2
sp|P11226|MBL2_HUMAN Mannose-binding protein C OS=Homo sapiens GN=MBL2 PE=1 SV=2
sp|Q8NDL9|CBPC5_HUMAN Cytosolic carboxypeptidase-like protein 5 OS=Homo sapiens GN=AGBL5 PE=2
A0A075B7D8|A0A075B7D8_HUMAN Protein IGHV3OR15-7 (Fragment) OS=Homo sapiens GN=IGHV3OR15-7 PE=1
sp|O95782|AP2A1_HUMAN AP-2 complex subunit alpha-1 OS=Homo sapiens GN=AP2A1 PE=1 SV=3
A0A075B6P5|A0A075B6P5_HUMAN Protein IGKV2-28 (Fragment) OS=Homo sapiens GN=IGKV2D-28 PE=1 SV=1
sp|B9ZVM9|TCP2L_HUMAN T-complex protein 10A homolog 2 OS=Homo sapiens GN=TCP10L2 PE=3 SV=2
sp|Q8IZL8|PELP1_HUMAN Proline- glutamic acid- and leucine-rich protein 1 OS=Homo sapiens GN=PELP1
sp|P55317|FOXA1_HUMAN Hepatocyte nuclear factor 3-alpha OS=Homo sapiens GN=FOXA1 PE=1 SV=2
sp|P31939|PUR9_HUMAN Bifunctional purine biosynthesis protein PURH OS=Homo sapiens GN=ATIC PE=
A0A087WSY4|A0A087WSY4_HUMAN Protein IGHV4-31 (Fragment) OS=Homo sapiens GN=IGHV4-31 PE=4 SV=1
sp|Q17RG1|KCD19_HUMAN BTB/POZ domain-containing protein KCTD19 OS=Homo sapiens GN=KCTD19 P
sp|Q9UNW9|NOVA2_HUMAN RNA-binding protein Nova-2 OS=Homo sapiens GN=NOVA2 PE=1 SV=1
sp|Q9Y4B5|MTCL1_HUMAN Microtubule cross-linking factor 1 OS=Homo sapiens GN=MTCL1 PE=1 SV=5
sp|Q9C0J8|WDR33_HUMAN pre-mRNA 3' end processing protein WDR33 OS=Homo sapiens GN=WDR33 PE
sp|P78381|S35A2_HUMAN UDP-galactose translocator OS=Homo sapiens GN=SLC35A2 PE=1 SV=1
sp|Q8NBR0|P5I13_HUMAN Tumor protein p53-inducible protein 13 OS=Homo sapiens GN=TP53I13 PE=2 S
sp|Q86Y38|XYLT1_HUMAN Xylosyltransferase 1 OS=Homo sapiens GN=XYLT1 PE=1 SV=1
sp|Q00872|MYPC1_HUMAN Myosin-binding protein C slow-type OS=Homo sapiens GN=MYBPC1 PE=1 SV=
sp|P37288|V1AR_HUMAN Vasopressin V1a receptor OS=Homo sapiens GN=AVPR1A PE=1 SV=1
sp|Q8N9B5|JMY_HUMAN Junction-mediating and -regulatory protein OS=Homo sapiens GN=JMY PE=1 S
sp|Q7Z406|MYH14_HUMAN Myosin-14 OS=Homo sapiens GN=MYH14 PE=1 SV=2
sp|Q92618|ZN516_HUMAN Zinc finger protein 516 OS=Homo sapiens GN=ZNF516 PE=1 SV=1
sp|P49908|SEPP1_HUMAN Selenoprotein P OS=Homo sapiens GN=SEPP1 PE=1 SV=3
sp|Q9NZC3|GDE1_HUMAN Glycerophosphodiester phosphodiesterase 1 OS=Homo sapiens GN=GDE1 PE=
sp|Q6P6C2|ALKB5_HUMAN RNA demethylase ALKBH5 OS=Homo sapiens GN=ALKBH5 PE=1 SV=2
sp|Q75VX8|GARE2_HUMAN GRB2-associated and regulator of MAPK protein 2 OS=Homo sapiens GN=GAR
sp|P09172|DOPO_HUMAN Dopamine beta-hydroxylase OS=Homo sapiens GN=DBH PE=1 SV=3
sp|Q5VWQ0|RSBN1_HUMAN Round spermatid basic protein 1 OS=Homo sapiens GN=RSBN1 PE=1 SV=2
sp|Q15735|PI5PA_HUMAN Phosphatidylinositol 4 5-bisphosphate 5-phosphatase A OS=Homo sapiens GN=
sp|Q5JUK3|KCNT1_HUMAN Potassium channel subfamily T member 1 OS=Homo sapiens GN=KCNT1 PE=1
sp|Q9NZW4|DSPP_HUMAN Dentin sialophosphoprotein OS=Homo sapiens GN=DSPP PE=1 SV=2
sp|Q96PM9|Z385A_HUMAN Zinc finger protein 385A OS=Homo sapiens GN=ZNF385A PE=1 SV=2
sp|P15814|IGLL1_HUMAN Immunoglobulin lambda-like polypeptide 1 OS=Homo sapiens GN=IGLL1 PE=1
sp|Q13233|M3K1_HUMAN Mitogen-activated protein kinase kinase kinase 1 OS=Homo sapiens GN=MAP3K
sp|Q8WTP8|AEN_HUMAN Apoptosis-enhancing nuclease OS=Homo sapiens GN=AEN PE=1 SV=2
sp|Q2TAC2|CCD57_HUMAN Coiled-coil domain-containing protein 57 OS=Homo sapiens GN=CCDC57 PE=1
sp|O00570|SOX1_HUMAN Transcription factor SOX-1 OS=Homo sapiens GN=SOX1 PE=1 SV=2
A0A075B6R9|A0A075B6R9_HUMAN Protein IGKV2D-24 (Fragment) OS=Homo sapiens GN=IGKV2D-24 PE=4 SV=1
sp|Q5T6F2|UBAP2_HUMAN Ubiquitin-associated protein 2 OS=Homo sapiens GN=UBAP2 PE=1 SV=1
sp|Q5SYE7|NHSL1_HUMAN NHS-like protein 1 OS=Homo sapiens GN=NHSL1 PE=1 SV=2
sp|Q07954|LRP1_HUMAN Prolow-density lipoprotein receptor-related protein 1 OS=Homo sapiens GN=LRP
sp|Q9NZP8|C1RL_HUMAN Complement C1r subcomponent-like protein OS=Homo sapiens GN=C1RL PE=1
sp|P02753|RET4_HUMAN Retinol-binding protein 4 OS=Homo sapiens GN=RBP4 PE=1 SV=3
sp|P02749|APOH_HUMAN Beta-2-glycoprotein 1 OS=Homo sapiens GN=APOH PE=1 SV=3
sp|P04196|HRG_HUMAN Histidine-rich glycoprotein OS=Homo sapiens GN=HRG PE=1 SV=1
sp|Q8TDN4|CABL1_HUMAN CDK5 and ABL1 enzyme substrate 1 OS=Homo sapiens GN=CABLES1 PE=1 S
sp|P04070|PROC_HUMAN Vitamin K-dependent protein C OS=Homo sapiens GN=PROC PE=1 SV=1
sp|P01008|ANT3_HUMAN Antithrombin-III OS=Homo sapiens GN=SERPINC1 PE=1 SV=1
sp|O15297|PPM1D_HUMAN Protein phosphatase 1D OS=Homo sapiens GN=PPM1D PE=1 SV=1
sp|P18206|VINC_HUMAN Vinculin OS=Homo sapiens GN=VCL PE=1 SV=4
sp|O00327|BMAL1_HUMAN Aryl hydrocarbon receptor nuclear translocator-like protein 1 OS=Homo sapiens
sp|Q16676|FOXD1_HUMAN Forkhead box protein D1 OS=Homo sapiens GN=FOXD1 PE=2 SV=1
sp|P15822|ZEP1_HUMAN Zinc finger protein 40 OS=Homo sapiens GN=HIVEP1 PE=1 SV=3
sp|P02788|TRFL_HUMAN Lactotransferrin OS=Homo sapiens GN=LTF PE=1 SV=6
sp|Q96PZ7|CSMD1_HUMAN CUB and sushi domain-containing protein 1 OS=Homo sapiens GN=CSMD1 PE=
sp|Q9BQS8|FYCO1_HUMAN FYVE and coiled-coil domain-containing protein 1 OS=Homo sapiens GN=FYCO
sp|Q9UDT6|CLIP2_HUMAN CAP-Gly domain-containing linker protein 2 OS=Homo sapiens GN=CLIP2 PE=1
sp|P18136|KV313_HUMAN Ig kappa chain V-III region HIC OS=Homo sapiens PE=1 SV=2
sp|Q9UPX8|SHAN2_HUMAN SH3 and multiple ankyrin repeat domains protein 2 OS=Homo sapiens GN=SHA
sp|O75376|NCOR1_HUMAN Nuclear receptor corepressor 1 OS=Homo sapiens GN=NCOR1 PE=1 SV=2
A0A075B6J9|A0A075B6J9_HUMAN HCG1782423 (Fragment) OS=Homo sapiens GN=IGLV2-18 PE=1 SV=1
sp|Q9HCE0|EPG5_HUMAN Ectopic P granules protein 5 homolog OS=Homo sapiens GN=EPG5 PE=1 SV=2
sp|Q15835|RK_HUMAN Rhodopsin kinase OS=Homo sapiens GN=GRK1 PE=1 SV=1
sp|Q12789|TF3C1_HUMAN General transcription factor 3C polypeptide 1 OS=Homo sapiens GN=GTF3C1 P
sp|Q9UBN7|HDAC6_HUMAN Histone deacetylase 6 OS=Homo sapiens GN=HDAC6 PE=1 SV=2
sp|Q9BQS7|HEPH_HUMAN Hephaestin OS=Homo sapiens GN=HEPH PE=1 SV=3
sp|P02458|CO2A1_HUMAN Collagen alpha-1(II) chain OS=Homo sapiens GN=COL2A1 PE=1 SV=3
sp|P05090|APOD_HUMAN Apolipoprotein D OS=Homo sapiens GN=APOD PE=1 SV=1
sp|Q5T0F9|C2D1B_HUMAN Coiled-coil and C2 domain-containing protein 1B OS=Homo sapiens GN=CC2D1
sp|Q14517|FAT1_HUMAN Protocadherin Fat 1 OS=Homo sapiens GN=FAT1 PE=1 SV=2
A0A075B6K5|A0A075B6K5_HUMAN HCG2043239 (Fragment) OS=Homo sapiens GN=IGLV3-9 PE=1 SV=1
A0A075B6J4|A0A075B6J4_HUMAN Protein IGLV3-25 (Fragment) OS=Homo sapiens GN=IGLV3-25 PE=1 SV=2
sp|P84550|SKOR1_HUMAN SKI family transcriptional corepressor 1 OS=Homo sapiens GN=SKOR1 PE=1 S
sp|Q9NWS8|RMND1_HUMAN Required for meiotic nuclear division protein 1 homolog OS=Homo sapiens GN=
sp|O14638|ENPP3_HUMAN Ectonucleotide pyrophosphatase/phosphodiesterase family member 3 OS=Homo
sp|Q6PJG6|BRAT1_HUMAN BRCA1-associated ATM activator 1 OS=Homo sapiens GN=BRAT1 PE=1 SV=2
sp|Q7Z392|TPC11_HUMAN Trafficking protein particle complex subunit 11 OS=Homo sapiens GN=TRAPPC1
sp|Q9Y3S1|WNK2_HUMAN Serine/threonine-protein kinase WNK2 OS=Homo sapiens GN=WNK2 PE=1 SV=
sp|Q68E01|INT3_HUMAN Integrator complex subunit 3 OS=Homo sapiens GN=INTS3 PE=1 SV=1
D6RGX4|D6RGX4_HUMAN Protein FAM90A26 OS=Homo sapiens GN=FAM90A26 PE=4 SV=1
sp|P59510|ATS20_HUMAN A disintegrin and metalloproteinase with thrombospondin motifs 20 OS=Homo sa

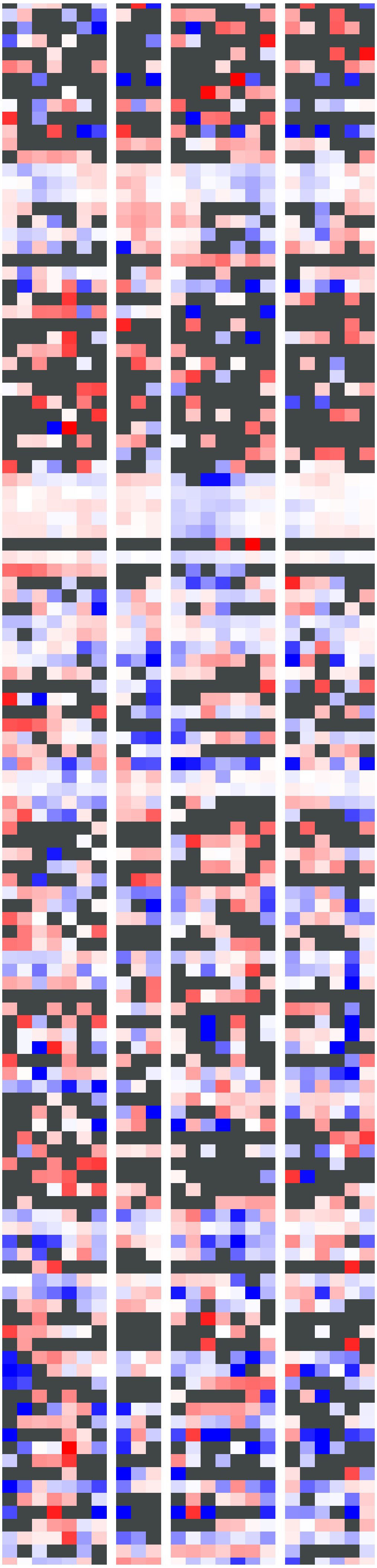

sp|P59510|ATS20_HUMAN A disintegrin and metalloproteinase with thrombospondin motifs 20 OS=Homo sa
sp|Q8WXG9|GPR98_HUMAN G-protein coupled receptor 98 OS=Homo sapiens GN=GPR98 PE=1 SV=2
sp|Q6K0P9|IFIX_HUMAN Pyrin and HIN domain-containing protein 1 OS=Homo sapiens GN=PYHIN1 PE=
sp|Q9NP31|SH22A_HUMAN SH2 domain-containing protein 2A OS=Homo sapiens GN=SH2D2A PE=1 SV=3
A0A075B6S2|A0A075B6S2_HUMAN Protein IGKV2D-29 (Fragment) OS=Homo sapiens GN=IGKV2D-29 PE=1 SV=1
sp|Q9H6K5|PRR36_HUMAN Proline-rich protein 36 OS=Homo sapiens GN=PRR36 PE=1 SV=2
sp|Q5JPE7|NOMO2_HUMAN Nodal modulator 2 OS=Homo sapiens GN=NOMO2 PE=1 SV=1
sp|Q8WVV9|HNRLL_HUMAN Heterogeneous nuclear ribonucleoprotein L-like OS=Homo sapiens GN=HNRNPL
sp|P04180|LCAT_HUMAN Phosphatidylcholine-sterol acyltransferase OS=Homo sapiens GN=LCAT PE=1 S
sp|Q9Y4G8|RPGF2_HUMAN Rap guanine nucleotide exchange factor 2 OS=Homo sapiens GN=RAPGEF2 PE
sp|Q5CZ79|AN20B_HUMAN Ankyrin repeat domain-containing protein 20B OS=Homo sapiens GN=ANKRD20
sp|Q13428|TCOF_HUMAN Treacle protein OS=Homo sapiens GN=TCOF1 PE=1 SV=3
sp|Q8TBF4|ZCRB1_HUMAN Zinc finger CCHC-type and RNA-binding motif-containing protein 1 OS=Homo sa
sp|Q8NE71|ABCF1_HUMAN ATP-binding cassette sub-family F member 1 OS=Homo sapiens GN=ABCF1 PE
sp|Q13049|TRI32_HUMAN E3 ubiquitin-protein ligase TRIM32 OS=Homo sapiens GN=TRIM32 PE=1 SV=2
sp|Q15406|NR6A1_HUMAN Nuclear receptor subfamily 6 group A member 1 OS=Homo sapiens GN=NR6A1
sp|Q8WUF5|IASPP_HUMAN RelA-associated inhibitor OS=Homo sapiens GN=PPP1R13L PE=1 SV=4
sp|A6NKL6|T200C_HUMAN Transmembrane protein 200C OS=Homo sapiens GN=TMEM200C PE=2 SV=2
sp|Q9BVL4|SELO_HUMAN Selenoprotein O OS=Homo sapiens GN=SELO PE=2 SV=3
sp|Q7Z6J2|GRASP_HUMAN General receptor for phosphoinositides 1-associated scaffold protein OS=Homo s
sp|P23141|EST1_HUMAN Liver carboxylesterase 1 OS=Homo sapiens GN=CES1 PE=1 SV=2
sp|Q6ZMW2|ZN782_HUMAN Zinc finger protein 782 OS=Homo sapiens GN=ZNF782 PE=2 SV=1
sp|Q9P260|K1468_HUMAN LisH domain and HEAT repeat-containing protein KIAA1468 OS=Homo sapiens G
sp|Q7Z5N4|SDK1_HUMAN Protein sidekick-1 OS=Homo sapiens GN=SDK1 PE=2 SV=3
sp|A8MW92|P20L1_HUMAN PHD finger protein 20-like protein 1 OS=Homo sapiens GN=PHF20L1 PE=1 SV=
sp|P50579|MAP2_HUMAN Methionine aminopeptidase 2 OS=Homo sapiens GN=METAP2 PE=1 SV=1
sp|P35716|SOX11_HUMAN Transcription factor SOX-11 OS=Homo sapiens GN=SOX11 PE=1 SV=2
sp|O60333|KIF1B_HUMAN Kinesin-like protein KIF1B OS=Homo sapiens GN=KIF1B PE=1 SV=5
sp|O00268|TAF4_HUMAN Transcription initiation factor TFIID subunit 4 OS=Homo sapiens GN=TAF4 PE=1
sp|Q96PX6|CC85A_HUMAN Coiled-coil domain-containing protein 85A OS=Homo sapiens GN=CCDC85A PE
sp|P05160|F13B_HUMAN Coagulation factor XIII B chain OS=Homo sapiens GN=F13B PE=1 SV=3
sp|Q9H7P9|PKHG2_HUMAN Pleckstrin homology domain-containing family G member 2 OS=Homo sapiens G
sp|Q9Y566|SHAN1_HUMAN SH3 and multiple ankyrin repeat domains protein 1 OS=Homo sapiens GN=SHAN
sp|Q9Y4G6|TLN2_HUMAN Talin-2 OS=Homo sapiens GN=TLN2 PE=1 SV=4
sp|Q13635|PTC1_HUMAN Protein patched homolog 1 OS=Homo sapiens GN=PTCH1 PE=1 SV=2
sp|O00391|QSOX1_HUMAN Sulfhydryl oxidase 1 OS=Homo sapiens GN=QSOX1 PE=1 SV=3
sp|O95197|RTN3_HUMAN Reticulon-3 OS=Homo sapiens GN=RTN3 PE=1 SV=2
sp|O95445|APOM_HUMAN Apolipoprotein M OS=Homo sapiens GN=APOM PE=1 SV=2
sp|P02774|VTDB_HUMAN Vitamin D-binding protein OS=Homo sapiens GN=GC PE=1 SV=1
sp|P19823|ITIH2_HUMAN Inter-alpha-trypsin inhibitor heavy chain H2 OS=Homo sapiens GN=ITIH2 PE=1 S
sp|P00747|PLMN_HUMAN Plasminogen OS=Homo sapiens GN=PLG PE=1 SV=2
sp|P01031|CO5_HUMAN Complement C5 OS=Homo sapiens GN=C5 PE=1 SV=4
sp|O15014|ZN609_HUMAN Zinc finger protein 609 OS=Homo sapiens GN=ZNF609 PE=1 SV=2
sp|P05156|CFAI_HUMAN Complement factor I OS=Homo sapiens GN=CFI PE=1 SV=2
sp|P06317|LV603_HUMAN Ig lambda chain V-VI region SUT OS=Homo sapiens PE=1 SV=1
sp|O95817|BAG3_HUMAN BAG family molecular chaperone regulator 3 OS=Homo sapiens GN=BAG3 PE=1
sp|P38570|ITAE_HUMAN Integrin alpha-E OS=Homo sapiens GN=ITGAE PE=1 SV=3
sp|Q13439|GOGA4_HUMAN Golgin subfamily A member 4 OS=Homo sapiens GN=GOLGA4 PE=1 SV=1
sp|Q76L83|ASXL2_HUMAN Putative Polycomb group protein ASXL2 OS=Homo sapiens GN=ASXL2 PE=1 S
sp|Q9H7P6|MB12B_HUMAN Multivesicular body subunit 12B OS=Homo sapiens GN=MVB12B PE=1 SV=2
sp|O75038|PLCH2_HUMAN 1-phosphatidylinositol 4 5-bisphosphate phosphodiesterase eta-2 OS=Homo sapi
sp|Q5THJ4|VP13D_HUMAN Vacuolar protein sorting-associated protein 13D OS=Homo sapiens GN=VPS13D
sp|Q9Y4E8|UBP15_HUMAN Ubiquitin carboxyl-terminal hydrolase 15 OS=Homo sapiens GN=USP15 PE=1 S
A0A0J9YVY3|A0A0J9YVY3_HUMAN Uncharacterized protein (Fragment) OS=Homo sapiens PE=1 SV=1
sp|Q96M02|CJ090_HUMAN Centrosomal protein C10orf90 OS=Homo sapiens GN=C10orf90 PE=2 SV=2
sp|Q9HB63|NET4_HUMAN Netrin-4 OS=Homo sapiens GN=NTN4 PE=1 SV=2
sp|P37840|SYUA_HUMAN Alpha-synuclein OS=Homo sapiens GN=SNCA PE=1 SV=1
sp|Q2VWA4|SKOR2_HUMAN SKI family transcriptional corepressor 2 OS=Homo sapiens GN=SKOR2 PE=1 S
sp|P49454|CENPF_HUMAN Centromere protein F OS=Homo sapiens GN=CENPF PE=1 SV=2
sp|Q9UKK3|PARP4_HUMAN Poly [ADP-ribose] polymerase 4 OS=Homo sapiens GN=PARP4 PE=1 SV=3
sp|P00748|FA12_HUMAN Coagulation factor XII OS=Homo sapiens GN=F12 PE=1 SV=3
sp|Q96T58|MINT_HUMAN Msx2-interacting protein OS=Homo sapiens GN=SPEN PE=1 SV=1
sp|Q8IXJ9|ASXL1_HUMAN Putative Polycomb group protein ASXL1 OS=Homo sapiens GN=ASXL1 PE=1 S
D6RIA3|D6RIA3_HUMAN Protein LOC285556 OS=Homo sapiens GN=LOC285556 PE=4 SV=1
sp|Q13751|LAMB3_HUMAN Laminin subunit beta-3 OS=Homo sapiens GN=LAMB3 PE=1 SV=1
sp|Q02833|RASF7_HUMAN Ras association domain-containing protein 7 OS=Homo sapiens GN=RASSF7 PE
sp|Q96SC8|DMTA2_HUMAN Doublesex- and mab-3-related transcription factor A2 OS=Homo sapiens GN=DM
sp|P02538|K2C6A_HUMAN Keratin type II cytoskeletal 6A OS=Homo sapiens GN=KRT6A PE=1 SV=3
sp|P55011|S12A2_HUMAN Solute carrier family 12 member 2 OS=Homo sapiens GN=SLC12A2 PE=1 SV=1
sp|O15018|PDZD2_HUMAN PDZ domain-containing protein 2 OS=Homo sapiens GN=PDZD2 PE=1 SV=4
sp|Q14050|CO9A3_HUMAN Collagen alpha-3(IX) chain OS=Homo sapiens GN=COL9A3 PE=1 SV=2
sp|Q12955|ANK3_HUMAN Ankyrin-3 OS=Homo sapiens GN=ANK3 PE=1 SV=3
sp|Q6ZUX3|F179A_HUMAN Protein FAM179A OS=Homo sapiens GN=FAM179A PE=2 SV=2
sp|O94761|RECQ4_HUMAN ATP-dependent DNA helicase Q4 OS=Homo sapiens GN=RECQL4 PE=1 SV=1
sp|P04275|VWF_HUMAN von Willebrand factor OS=Homo sapiens GN=VWF PE=1 SV=4
sp|Q9Y2X9|ZN281_HUMAN Zinc finger protein 281 OS=Homo sapiens GN=ZNF281 PE=1 SV=1
sp|Q96HR3|MED30_HUMAN Mediator of RNA polymerase II transcription subunit 30 OS=Homo sapiens GN=M
sp|P04278|SHBG_HUMAN Sex hormone-binding globulin OS=Homo sapiens GN=SHBG PE=1 SV=2
sp|P09874|PARP1_HUMAN Poly [ADP-ribose] polymerase 1 OS=Homo sapiens GN=PARP1 PE=1 SV=4
sp|Q96JB1|DYH8_HUMAN Dynein heavy chain 8 axonemal OS=Homo sapiens GN=DNAH8 PE=1 SV=2
sp|Q9C009|FOXQ1_HUMAN Forkhead box protein Q1 OS=Homo sapiens GN=FOXQ1 PE=2 SV=2
sp|O75691|UTP20_HUMAN Small subunit processome component 20 homolog OS=Homo sapiens GN=UTP2
sp|P35663|CYLC1_HUMAN Cylicin-1 OS=Homo sapiens GN=CYLC1 PE=2 SV=2
sp|Q6NS38|ALKB2_HUMAN DNA oxidative demethylase ALKBH2 OS=Homo sapiens GN=ALKBH2 PE=1 SV=
sp|Q92793|CBP_HUMAN CREB-binding protein OS=Homo sapiens GN=CREBBP PE=1 SV=3
A0A0B4J1U1|A0A0B4J1U1_HUMAN Protein IGLV1-44 (Fragment) OS=Homo sapiens GN=IGLV1-44 PE=1 SV=5
sp|A6NLU5|VTM2B_HUMAN V-set and transmembrane domain-containing protein 2B OS=Homo sapiens GN=
sp|O75161|NPHP4_HUMAN Nephrocystin-4 OS=Homo sapiens GN=NPHP4 PE=1 SV=2
sp|P35858|ALS_HUMAN Insulin-like growth factor-binding protein complex acid labile subunit OS=Homo sa
sp|Q8TF72|SHRM3_HUMAN Protein Shroom3 OS=Homo sapiens GN=SHROOM3 PE=1 SV=2
sp|P29376|LTK_HUMAN Leukocyte tyrosine kinase receptor OS=Homo sapiens GN=LTK PE=1 SV=3
sp|P04206|KV307_HUMAN Ig kappa chain V-III region GOL OS=Homo sapiens PE=1 SV=1
sp|Q15399|TLR1_HUMAN Toll-like receptor 1 OS=Homo sapiens GN=TLR1 PE=1 SV=3
sp|P07305|H10_HUMAN Histone H1.0 OS=Homo sapiens GN=H1F0 PE=1 SV=3
sp|Q9NWH9|SLTM_HUMAN SAFB-like transcription modulator OS=Homo sapiens GN=SLTM PE=1 SV=2
sp|P02461|CO3A1_HUMAN Collagen alpha-1(III) chain OS=Homo sapiens GN=COL3A1 PE=1 SV=4
sp|Q9P267|MBD5_HUMAN Methyl-CpG-binding domain protein 5 OS=Homo sapiens GN=MBD5 PE=1 SV=3
sp|Q9UEW3|MARCO_HUMAN Macrophage receptor MARCO OS=Homo sapiens GN=MARCO PE=1 SV=1
sp|Q7LFL8|CXXC5_HUMAN CXXC-type zinc finger protein 5 OS=Homo sapiens GN=CXXC5 PE=1 SV=1
sp|Q92797|SYMPK_HUMAN Symplekin OS=Homo sapiens GN=SYMPK PE=1 SV=2
sp|Q4ZHG4|FNDC1_HUMAN Fibronectin type III domain-containing protein 1 OS=Homo sapiens GN=FNDC1 P
sp|P12109|CO6A1_HUMAN Collagen alpha-1(VI) chain OS=Homo sapiens GN=COL6A1 PE=1 SV=3
sp|Q96PE1|AGRA2_HUMAN Adhesion G protein-coupled receptor A2 OS=Homo sapiens GN=ADGRA2 PE=1
sp|Q9Y5Y9|SCNAA_HUMAN Sodium channel protein type 10 subunit alpha OS=Homo sapiens GN=SCN10A P
sp|Q6KC79|NIPBL_HUMAN Nipped-B-like protein OS=Homo sapiens GN=NIPBL PE=1 SV=2
sp|Q08378|GOGA3_HUMAN Golgin subfamily A member 3 OS=Homo sapiens GN=GOLGA3 PE=1 SV=2
sp|Q32MK0|MYLK3_HUMAN Myosin light chain kinase 3 OS=Homo sapiens GN=MYLK3 PE=1 SV=3
sp|P04209|LV211_HUMAN Ig lambda chain V-II region NIG-84 OS=Homo sapiens PE=1 SV=1
sp|Q96G46|DUS3L_HUMAN tRNA-dihydrouridine(47) synthase [NAD(P)(+)]-like OS=Homo sapiens GN=DUS3
sp|Q14669|TRIPC_HUMAN E3 ubiquitin-protein ligase TRIP12 OS=Homo sapiens GN=TRIP12 PE=1 SV=1
sp|Q9P1D8|YP008_HUMAN Putative uncharacterized protein PRO2289 OS=Homo sapiens GN=PRO2289 PE
sp|Q8TDG2|ACTT1_HUMAN Actin-related protein T1 OS=Homo sapiens GN=ACTRT1 PE=2 SV=2
sp|Q96L73|NSD1_HUMAN Histone-lysine N-methyltransferase H3 lysine-36 and H4 lysine-20 specific OS=H
sp|Q8TF21|ANR24_HUMAN Ankyrin repeat domain-containing protein 24 OS=Homo sapiens GN=ANKRD24 P
sp|Q15021|CND1_HUMAN Condensin complex subunit 1 OS=Homo sapiens GN=NCAPD2 PE=1 SV=3
sp|Q86VR7|VS10L_HUMAN V-set and immunoglobulin domain-containing protein 10-like OS=Homo sapiens G
sp|P05187|PPB1_HUMAN Alkaline phosphatase placental type OS=Homo sapiens GN=ALPP PE=1 SV=2
sp|Q96A09|FA46B_HUMAN Protein FAM46B OS=Homo sapiens GN=FAM46B PE=1 SV=2
sp|Q9Y3M8|STA13_HUMAN StAR-related lipid transfer protein 13 OS=Homo sapiens GN=STARD13 PE=1 SV
sp|Q6ZSY5|PPR3F_HUMAN Protein phosphatase 1 regulatory subunit 3F OS=Homo sapiens GN=PPP1R3F P
sp|Q96QT6|PHF12_HUMAN PHD finger protein 12 OS=Homo sapiens GN=PHF12 PE=1 SV=2
[illegible]

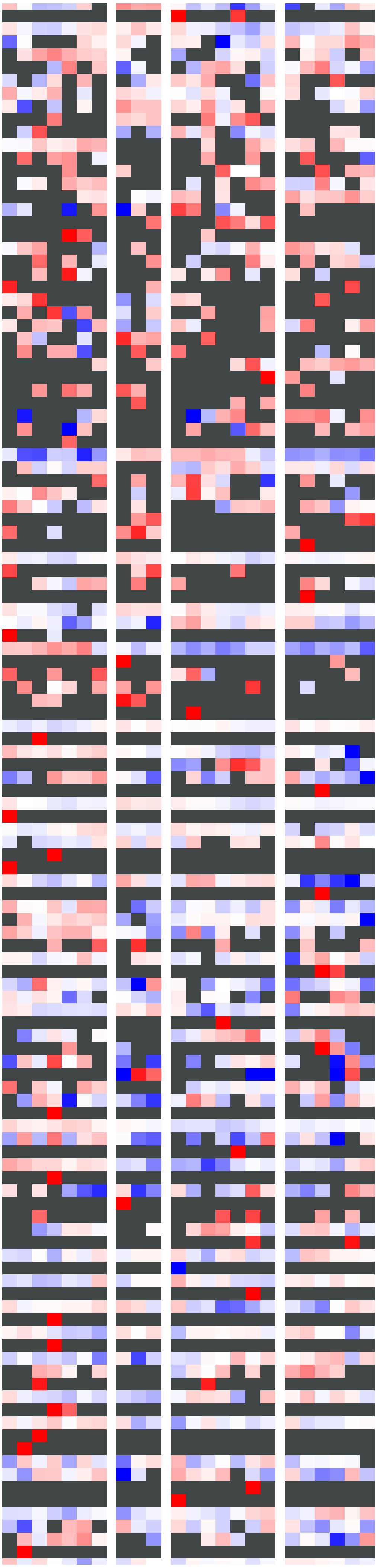
sp|Q02952|AKA12_HUMAN A-kinase anchor protein 12 OS=Homo sapiens GN=AKAP12 PE=1 SV=4
sp|Q9UPY3|DICER_HUMAN Endoribonuclease Dicer OS=Homo sapiens GN=DICER1 PE=1 SV=3
sp|P14923|PLAK_HUMAN Junction plakoglobin OS=Homo sapiens GN=JUP PE=1 SV=3
sp|Q86V81|THOC4_HUMAN THO complex subunit 4 OS=Homo sapiens GN=ALYREF PE=1 SV=3
sp|Q9NUV9|GIMA4_HUMAN GTPase IMAP family member 4 OS=Homo sapiens GN=GIMAP4 PE=1 SV=1
sp|Q6P1W5|CA094_HUMAN Uncharacterized protein C1orf94 OS=Homo sapiens GN=C1orf94 PE=1 SV=2
sp|Q08345|DDR1_HUMAN Epithelial discoidin domain-containing receptor 1 OS=Homo sapiens GN=DDR1 F
sp|Q14690|RRP5_HUMAN Protein RRP5 homolog OS=Homo sapiens GN=PDCD11 PE=1 SV=3
sp|Q9C0G0|ZN407_HUMAN Zinc finger protein 407 OS=Homo sapiens GN=ZNF407 PE=1 SV=2
sp|P63261|ACTG_HUMAN Actin cytoplasmic 2 OS=Homo sapiens GN=ACTG1 PE=1 SV=1
sp|Q9H0W5|CCDC8_HUMAN Coiled-coil domain-containing protein 8 OS=Homo sapiens GN=CCDC8 PE=1 SV
sp|Q13492|PICAL_HUMAN Phosphatidylinositol-binding clathrin assembly protein OS=Homo sapiens GN=PI
sp|Q9C0D6|FHDC1_HUMAN FH2 domain-containing protein 1 OS=Homo sapiens GN=FHDC1 PE=1 SV=2
sp|Q9UMF0|ICAM5_HUMAN Intercellular adhesion molecule 5 OS=Homo sapiens GN=ICAM5 PE=1 SV=3
sp|Q9UKA4|AKA11_HUMAN A-kinase anchor protein 11 OS=Homo sapiens GN=AKAP11 PE=1 SV=1
sp|Q86VN1|VPS36_HUMAN Vacuolar protein-sorting-associated protein 36 OS=Homo sapiens GN=VPS36 PE
sp|P78363|ABCA4_HUMAN Retinal-specific ATP-binding cassette transporter OS=Homo sapiens GN=ABCA4
sp|Q15262|PTPRK_HUMAN Receptor-type tyrosine-protein phosphatase kappa OS=Homo sapiens GN=PTPF
sp|P01768|HV330_HUMAN Immunoglobulin heavy variable 3-30 OS=Homo sapiens GN=IGHV3-30 PE=1 SV
sp|Q9Y5B0|CTDP1_HUMAN RNA polymerase II subunit A C-terminal domain phosphatase OS=Homo sapiens
sp|Q7Z333|SETX_HUMAN Probable helicase senataxin OS=Homo sapiens GN=SETX PE=1 SV=4
sp|Q9BQG1|SYT3_HUMAN Synaptotagmin-3 OS=Homo sapiens GN=SYT3 PE=2 SV=1
sp|P26640|SYVC_HUMAN Valine--tRNA ligase OS=Homo sapiens GN=VARS PE=1 SV=4
sp|A6NCS6|CB072_HUMAN Uncharacterized protein C2orf72 OS=Homo sapiens GN=C2orf72 PE=1 SV=2
sp|P51124|GRAM_HUMAN Granzyme M OS=Homo sapiens GN=GZMM PE=1 SV=2
sp|P18858|DNLI1_HUMAN DNA ligase 1 OS=Homo sapiens GN=LIG1 PE=1 SV=1
sp|Q14980|NUMA1_HUMAN Nuclear mitotic apparatus protein 1 OS=Homo sapiens GN=NUMA1 PE=1 SV=2
sp|P24462|CP3A7_HUMAN Cytochrome P450 3A7 OS=Homo sapiens GN=CYP3A7 PE=1 SV=2
sp|P55058|PLTP_HUMAN Phospholipid transfer protein OS=Homo sapiens GN=PLTP PE=1 SV=1
A0A0C4DH35|A0A0C4DH35_HUMAN Protein IGHV3-35 (Fragment) OS=Homo sapiens GN=IGHV3-35 PE=1 SV=1
sp|Q03169|TNAP2_HUMAN Tumor necrosis factor alpha-induced protein 2 OS=Homo sapiens GN=TNFAIP2
sp|Q86UU1|PHLB1_HUMAN Pleckstrin homology-like domain family B member 1 OS=Homo sapiens GN=PHL
sp|P27987|IP3KB_HUMAN Inositol-trisphosphate 3-kinase B OS=Homo sapiens GN=ITPKB PE=1 SV=5
sp|Q5T0Z8|CF132_HUMAN Uncharacterized protein C6orf132 OS=Homo sapiens GN=C6orf132 PE=1 SV=4
sp|P01613|KV121_HUMAN Ig kappa chain V-I region Ni OS=Homo sapiens PE=1 SV=1
sp|Q8IYA6|CKP2L_HUMAN Cytoskeleton-associated protein 2-like OS=Homo sapiens GN=CKAP2L PE=1 SV
sp|Q13615|MTMR3_HUMAN Myotubularin-related protein 3 OS=Homo sapiens GN=MTMR3 PE=1 SV=3
sp|Q8NDG6|TDRD9_HUMAN Putative ATP-dependent RNA helicase TDRD9 OS=Homo sapiens GN=TDRD9 F
sp|Q8N307|MUC20_HUMAN Mucin-20 OS=Homo sapiens GN=MUC20 PE=1 SV=3
sp|Q92563|TICN2_HUMAN Testican-2 OS=Homo sapiens GN=SPOCK2 PE=1 SV=1
sp|Q9Y6R4|M3K4_HUMAN Mitogen-activated protein kinase kinase kinase 4 OS=Homo sapiens GN=MAP3K
sp|Q709C8|VP13C_HUMAN Vacuolar protein sorting-associated protein 13C OS=Homo sapiens GN=VPS13C
sp|P14151|LYAM1_HUMAN L-selectin OS=Homo sapiens GN=SELL PE=1 SV=2
sp|P46109|CRKL_HUMAN Crk-like protein OS=Homo sapiens GN=CRKL PE=1 SV=1
sp|Q96P48|ARAP1_HUMAN Arf-GAP with Rho-GAP domain ANK repeat and PH domain-containing protein 1
sp|Q9NUQ8|ABCF3_HUMAN ATP-binding cassette sub-family F member 3 OS=Homo sapiens GN=ABCF3 PE
sp|O00458|IFRD1_HUMAN Interferon-related developmental regulator 1 OS=Homo sapiens GN=IFRD1 PE=
sp|O60218|AK1BA_HUMAN Aldo-keto reductase family 1 member B10 OS=Homo sapiens GN=AKR1B10 PE
sp|P0DJI8|SAA1_HUMAN Serum amyloid A-1 protein OS=Homo sapiens GN=SAA1 PE=1 SV=1
sp|P00441|SODC_HUMAN Superoxide dismutase [Cu-Zn] OS=Homo sapiens GN=SOD1 PE=1 SV=2
sp|P01860|IGHG3_HUMAN Ig gamma-3 chain C region OS=Homo sapiens GN=IGHG3 PE=1 SV=2
sp|Q9NZ56|FMN2_HUMAN Formin-2 OS=Homo sapiens GN=FMN2 PE=1 SV=4
sp|Q17R98|ZN827_HUMAN Zinc finger protein 827 OS=Homo sapiens GN=ZNF827 PE=1 SV=1
sp|Q6NUT3|MFS12_HUMAN Major facilitator superfamily domain-containing protein 12 OS=Homo sapiens GN
sp|Q12946|FOXF1_HUMAN Forkhead box protein F1 OS=Homo sapiens GN=FOXF1 PE=1 SV=2
sp|O15213|WDR46_HUMAN WD repeat-containing protein 46 OS=Homo sapiens GN=WDR46 PE=1 SV=3
sp|Q13547|HDAC1_HUMAN Histone deacetylase 1 OS=Homo sapiens GN=HDAC1 PE=1 SV=1
sp|O15083|ERC2_HUMAN ERC protein 2 OS=Homo sapiens GN=ERC2 PE=1 SV=3
sp|Q6ZRS4|CC129_HUMAN Coiled-coil domain-containing protein 129 OS=Homo sapiens GN=CCDC129 PE
sp|Q9ULL0|K1210_HUMAN Uncharacterized protein KIAA1210 OS=Homo sapiens GN=KIAA1210 PE=2 SV=
sp|Q14151|SAFB2_HUMAN Scaffold attachment factor B2 OS=Homo sapiens GN=SAFB2 PE=1 SV=1
sp|Q9HCM7|FBSL_HUMAN Fibrosin-1-like protein OS=Homo sapiens GN=FBRSL1 PE=1 SV=4
sp|Q92616|GCN1_HUMAN eIF-2-alpha kinase activator GCN1 OS=Homo sapiens GN=GCN1 PE=1 SV=6
sp|P10809|CH60_HUMAN 60 kDa heat shock protein mitochondrial OS=Homo sapiens GN=HSPD1 PE=1 S
sp|Q86WI1|PKHL1_HUMAN Fibrocystin-L OS=Homo sapiens GN=PKHD1L1 PE=2 SV=2
sp|P55265|DSRAD_HUMAN Double-stranded RNA-specific adenosine deaminase OS=Homo sapiens GN=AD
sp|P69892|HBG2_HUMAN Hemoglobin subunit gamma-2 OS=Homo sapiens GN=HBG2 PE=1 SV=2
sp|Q09028|RBBP4_HUMAN Histone-binding protein RBBP4 OS=Homo sapiens GN=RBBP4 PE=1 SV=3
sp|Q9Y336|SIGL9_HUMAN Sialic acid-binding Ig-like lectin 9 OS=Homo sapiens GN=SIGLEC9 PE=1 SV=2
sp|Q8TDM6|DLG5_HUMAN Disks large homolog 5 OS=Homo sapiens GN=DLG5 PE=1 SV=4
sp|A6H8Y1|BDP1_HUMAN Transcription factor TFIIIB component B'' homolog OS=Homo sapiens GN=BDP1
sp|Q9NPJ6|MED4_HUMAN Mediator of RNA polymerase II transcription subunit 4 OS=Homo sapiens GN=ME
sp|Q96JI7|SPTCS_HUMAN Spatacsin OS=Homo sapiens GN=SPG11 PE=1 SV=3
sp|Q03252|LMNB2_HUMAN Lamin-B2 OS=Homo sapiens GN=LMNB2 PE=1 SV=4
sp|Q92841|DDX17_HUMAN Probable ATP-dependent RNA helicase DDX17 OS=Homo sapiens GN=DDX17 F
sp|Q9C0B7|TNG6_HUMAN Transport and Golgi organization protein 6 homolog OS=Homo sapiens GN=TAN
sp|Q9H0J4|QRIC2_HUMAN Glutamine-rich protein 2 OS=Homo sapiens GN=QRICH2 PE=1 SV=1
sp|Q13045|FLII_HUMAN Protein flightless-1 homolog OS=Homo sapiens GN=FLII PE=1 SV=2
sp|Q13387|JIP2_HUMAN C-Jun-amino-terminal kinase-interacting protein 2 OS=Homo sapiens GN=MAPK
sp|Q70EL4|UBP43_HUMAN Ubiquitin carboxyl-terminal hydrolase 43 OS=Homo sapiens GN=USP43 PE=1 S
sp|A6NJZ7|RIM3C_HUMAN RIMS-binding protein 3C OS=Homo sapiens GN=RIMBP3C PE=1 SV=3
sp|P04198|MYCN_HUMAN N-myc proto-oncogene protein OS=Homo sapiens GN=MYCN PE=1 SV=2
sp|Q9BYB0|SHAN3_HUMAN SH3 and multiple ankyrin repeat domains protein 3 OS=Homo sapiens GN=SHAN
sp|A0AVI2|FR1L5_HUMAN Fer-1-like protein 5 OS=Homo sapiens GN=FER1L5 PE=2 SV=2
sp|O95153|RIMB1_HUMAN Peripheral-type benzodiazepine receptor-associated protein 1 OS=Homo sapiens
sp|Q7Z628|ARHG8_HUMAN Neuroepithelial cell-transforming gene 1 protein OS=Homo sapiens GN=NET1 PE
sp|Q13162|PRDX4_HUMAN Peroxiredoxin-4 OS=Homo sapiens GN=PRDX4 PE=1 SV=1
sp|Q5T1N1|AKND1_HUMAN Protein AKNAD1 OS=Homo sapiens GN=AKNAD1 PE=2 SV=3
sp|Q8IVF4|DYH10_HUMAN Dynein heavy chain 10 axonemal OS=Homo sapiens GN=DNAH10 PE=1 SV=4
sp|Q9Y285|SYFA_HUMAN Phenylalanine--tRNA ligase alpha subunit OS=Homo sapiens GN=FARSA PE=1
sp|Q9H479|FN3K_HUMAN Fructosamine-3-kinase OS=Homo sapiens GN=FN3K PE=1 SV=1
sp|P62937|PPIA_HUMAN Peptidyl-prolyl cis-trans isomerase A OS=Homo sapiens GN=PPIA PE=1 SV=2
sp|C9J798|RAS4B_HUMAN Ras GTPase-activating protein 4B OS=Homo sapiens GN=RASA4B PE=3 SV=2
sp|Q14767|LTBP2_HUMAN Latent-transforming growth factor beta-binding protein 2 OS=Homo sapiens GN=I
sp|Q86SQ0|PHLB2_HUMAN Pleckstrin homology-like domain family B member 2 OS=Homo sapiens GN=PHL
sp|Q9NPP4|NLRC4_HUMAN NLR family CARD domain-containing protein 4 OS=Homo sapiens GN=NLRC4 P
sp|Q2T9J0|TYSD1_HUMAN Peroxisomal leader peptide-processing protease OS=Homo sapiens GN=TYSND
sp|P53618|COPB_HUMAN Coatomer subunit beta OS=Homo sapiens GN=COPB1 PE=1 SV=3
sp|Q9NYQ7|CELR3_HUMAN Cadherin EGF LAG seven-pass G-type receptor 3 OS=Homo sapiens GN=CELS
sp|Q70YC5|ZN365_HUMAN Protein ZNF365 OS=Homo sapiens GN=ZNF365 PE=1 SV=3
sp|O75064|DEN4B_HUMAN DENN domain-containing protein 4B OS=Homo sapiens GN=DENND4B PE=1 SV
sp|Q00975|CAC1B_HUMAN Voltage-dependent N-type calcium channel subunit alpha-1B OS=Homo sapiens
sp|Q9H6Z4|RANB3_HUMAN Ran-binding protein 3 OS=Homo sapiens GN=RANBP3 PE=1 SV=1
sp|Q13868|EXOS2_HUMAN Exosome complex component RRP4 OS=Homo sapiens GN=EXOSC2 PE=1 SV
sp|O43491|E41L2_HUMAN Band 4.1-like protein 2 OS=Homo sapiens GN=EPB41L2 PE=1 SV=1
sp|Q9UBZ9|REV1_HUMAN DNA repair protein REV1 OS=Homo sapiens GN=REV1 PE=1 SV=1
sp|Q12882|DPYD_HUMAN Dihydropyrimidine dehydrogenase [NADP(+)] OS=Homo sapiens GN=DPYD PE=
E9PCH4|E9PCH4_HUMAN Uncharacterized protein OS=Homo sapiens PE=4 SV=1
sp|P39880|CUX1_HUMAN Homeobox protein cut-like 1 OS=Homo sapiens GN=CUX1 PE=1 SV=3
sp|Q9NUD5|ZCHC3_HUMAN Zinc finger CCHC domain-containing protein 3 OS=Homo sapiens GN=ZCCHC3
sp|Q9H322|VCX2_HUMAN Variable charge X-linked protein 2 OS=Homo sapiens GN=VCX2 PE=2 SV=3
sp|P43251|BTD_HUMAN Biotinidase OS=Homo sapiens GN=BTD PE=1 SV=2
sp|Q9Y663|HS3SA_HUMAN Heparan sulfate glucosamine 3-O-sulfotransferase 3A1 OS=Homo sapiens GN=H
sp|O43593|HAIR_HUMAN Lysine-specific demethylase hairless OS=Homo sapiens GN=HR PE=1 SV=5
sp|O00159|MYO1C_HUMAN Unconventional myosin-Ic OS=Homo sapiens GN=MYO1C PE=1 SV=4
sp|Q14674|ESPL1_HUMAN Separin OS=Homo sapiens GN=ESPL1 PE=1 SV=3
sp|Q9H7N4|SFR19_HUMAN Splicing factor arginine/serine-rich 19 OS=Homo sapiens GN=SCAF1 PE=1 SV=
sp|Q7RTS7|K2C74_HUMAN Keratin type II cytoskeletal 74 OS=Homo sapiens GN=KRT74 PE=1 SV=2
sp|Q68CQ1|MROH7_HUMAN Maestro heat-like repeat-containing protein family member 7 OS=Homo sapiens
sp|Q9BRS8|LARP6_HUMAN La-related protein 6 OS=Homo sapiens GN=LARP6 PE=1 SV=1
sp|Q08830|FGL1_HUMAN Fibrinogen-like protein 1 OS=Homo sapiens GN=FGL1 PE=1 SV=3

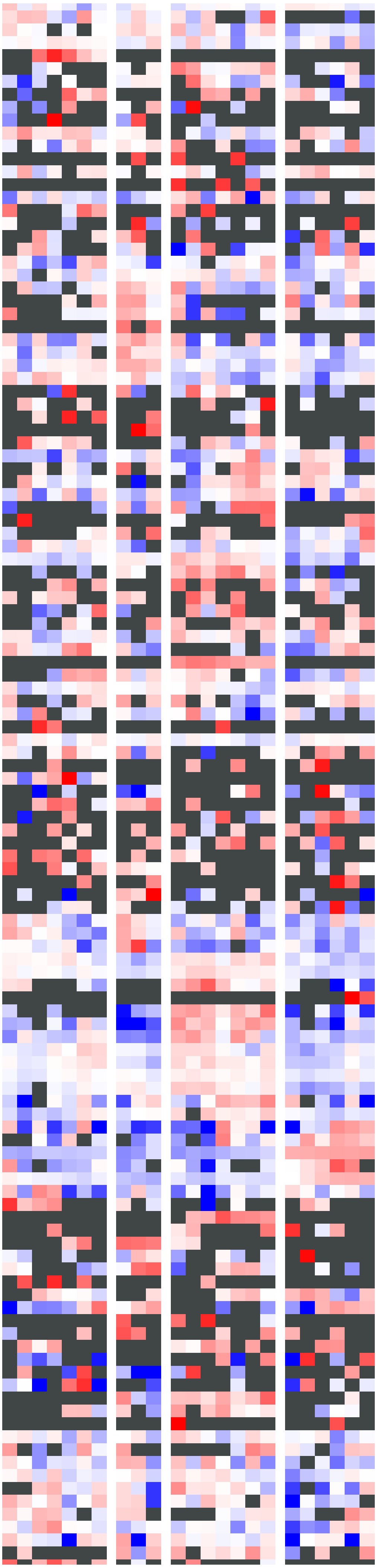

sp|P49916|DNLI3_HUMAN	DNA ligase 3 OS=Homo sapiens GN=LIG3 PE=1 SV=2
sp|Q6V1P9|PCD23_HUMAN	Protocadherin-23 OS=Homo sapiens GN=DCHS2 PE=2 SV=1
sp|Q9NR63|CP26B_HUMAN	Cytochrome P450 26B1 OS=Homo sapiens GN=CYP26B1 PE=1 SV=1
sp|Q9Y6R7|FCGBP_HUMAN	IgGFc-binding protein OS=Homo sapiens GN=FCGBP PE=1 SV=3
sp|O43147|SGSM2_HUMAN	Small G protein signaling modulator 2 OS=Homo sapiens GN=SGSM2 PE=1 SV=
sp|Q14207|NPAT_HUMAN	Protein NPAT OS=Homo sapiens GN=NPAT PE=1 SV=3
sp|Q5T0W9|FA83B_HUMAN	Protein FAM83B OS=Homo sapiens GN=FAM83B PE=1 SV=1
sp|Q9P0L2|MARK1_HUMAN	Serine/threonine-protein kinase MARK1 OS=Homo sapiens GN=MARK1 PE=1 S
sp|Q05516|ZBT16_HUMAN	Zinc finger and BTB domain-containing protein 16 OS=Homo sapiens GN=ZBTB
sp|Q562F6|SGO2_HUMAN	Shugoshin 2 OS=Homo sapiens GN=SGO2 PE=1 SV=2
sp|O00187|MASP2_HUMAN	Mannan-binding lectin serine protease 2 OS=Homo sapiens GN=MASP2 PE=1 S
sp|O96028|NSD2_HUMAN	Histone-lysine N-methyltransferase NSD2 OS=Homo sapiens GN=WHSC1 PE=1
sp|Q96L91|EP400_HUMAN	E1A-binding protein p400 OS=Homo sapiens GN=EP400 PE=1 SV=4
sp|Q8N1X5|YF001_HUMAN	Uncharacterized protein FLJ37310 OS=Homo sapiens PE=2 SV=1
sp|P06309|KV205_HUMAN	Ig kappa chain V-II region GM607 (Fragment) OS=Homo sapiens PE=4 SV=1
sp|A6NNE9|MARHB_HUMAN	E3 ubiquitin-protein ligase MARCH11 OS=Homo sapiens GN=MARCH11 PE=2 S
sp|Q70CQ4|UBP31_HUMAN	Ubiquitin carboxyl-terminal hydrolase 31 OS=Homo sapiens GN=USP31 PE=2 S
sp|Q15672|TWST1_HUMAN	Twist-related protein 1 OS=Homo sapiens GN=TWIST1 PE=1 SV=1
sp|Q9ULJ7|ANR50_HUMAN	Ankyrin repeat domain-containing protein 50 OS=Homo sapiens GN=ANKRD50 F
sp|Q9Y520|PRC2C_HUMAN	Protein PRRC2C OS=Homo sapiens GN=PRRC2C PE=1 SV=4
sp|P21359|NF1_HUMAN	Neurofibromin OS=Homo sapiens GN=NF1 PE=1 SV=2
sp|Q9UHY8|FEZ2_HUMAN	Fasciculation and elongation protein zeta-2 OS=Homo sapiens GN=FEZ2 PE=1 S
sp|Q9P2D1|CHD7_HUMAN	Chromodomain-helicase-DNA-binding protein 7 OS=Homo sapiens GN=CHD7 P
sp|P46940|IQGA1_HUMAN	Ras GTPase-activating-like protein IQGAP1 OS=Homo sapiens GN=IQGAP1 PE
sp|Q9HB15|KCNKC_HUMAN	Potassium channel subfamily K member 12 OS=Homo sapiens GN=KCNK12 PE
sp|Q13255|GRM1_HUMAN	Metabotropic glutamate receptor 1 OS=Homo sapiens GN=GRM1 PE=1 SV=3
sp|A7E2V4|ZSWM8_HUMAN	Zinc finger SWIM domain-containing protein 8 OS=Homo sapiens GN=ZSWIM8 F
sp|Q8IWN7|RP1L1_HUMAN	Retinitis pigmentosa 1-like 1 protein OS=Homo sapiens GN=RP1L1 PE=1 SV=4
sp|Q9BZF2|OSBL7_HUMAN	Oxysterol-binding protein-related protein 7 OS=Homo sapiens GN=OSBPL7 PE=
sp|Q9C0B2|CFA74_HUMAN	Cilia- and flagella-associated protein 74 OS=Homo sapiens GN=CFAP74 PE=2 S
sp|Q8IWY9|CDAN1_HUMAN	Codanin-1 OS=Homo sapiens GN=CDAN1 PE=1 SV=4
sp|Q8N1G0|ZN687_HUMAN	Zinc finger protein 687 OS=Homo sapiens GN=ZNF687 PE=1 SV=1
sp|Q9P2A4|ABI3_HUMAN	ABI gene family member 3 OS=Homo sapiens GN=ABI3 PE=1 SV=2
sp|Q8IU81|I2BP1_HUMAN	Interferon regulatory factor 2-binding protein 1 OS=Homo sapiens GN=IRF2BP1 I
sp|Q5VTB9|RN220_HUMAN	E3 ubiquitin-protein ligase RNF220 OS=Homo sapiens GN=RNF220 PE=1 SV=1
sp|Q9BQI6|SLF1_HUMAN	SMC5-SMC6 complex localization factor protein 1 OS=Homo sapiens GN=SLF1
sp|Q01167|FOXK2_HUMAN	Forkhead box protein K2 OS=Homo sapiens GN=FOXK2 PE=1 SV=3
sp|Q9P225|DYH2_HUMAN	Dynein heavy chain 2 axonemal OS=Homo sapiens GN=DNAH2 PE=2 SV=3
sp|Q00839|HNRPU_HUMAN	Heterogeneous nuclear ribonucleoprotein U OS=Homo sapiens GN=HNRNPU P
sp|P01611|KV119_HUMAN	Ig kappa chain V-I region Wes OS=Homo sapiens PE=1 SV=1
sp|P12036|NFH_HUMAN	Neurofilament heavy polypeptide OS=Homo sapiens GN=NEFH PE=1 SV=4
sp|Q96PN6|ADCYA_HUMAN	Adenylate cyclase type 10 OS=Homo sapiens GN=ADCY10 PE=1 SV=3
sp|Q14332|FZD2_HUMAN	Frizzled-2 OS=Homo sapiens GN=FZD2 PE=1 SV=1
sp|P02462|CO4A1_HUMAN	Collagen alpha-1(IV) chain OS=Homo sapiens GN=COL4A1 PE=1 SV=3
sp|Q6GYQ0|RGPA1_HUMAN	Ral GTPase-activating protein subunit alpha-1 OS=Homo sapiens GN=RALGAP
sp|A5PKW4|PSD1_HUMAN	PH and SEC7 domain-containing protein 1 OS=Homo sapiens GN=PSD PE=1 S
sp|Q5T8A7|PPR26_HUMAN	Protein phosphatase 1 regulatory subunit 26 OS=Homo sapiens GN=PPP1R26 F
sp|Q04725|TLE2_HUMAN	Transducin-like enhancer protein 2 OS=Homo sapiens GN=TLE2 PE=1 SV=2
sp|Q9NX62|IMPA3_HUMAN	Inositol monophosphatase 3 OS=Homo sapiens GN=IMPAD1 PE=1 SV=1
sp|Q3KQU3|MA7D1_HUMAN	MAP7 domain-containing protein 1 OS=Homo sapiens GN=MAP7D1 PE=1 SV=1
sp|P08519|APOA_HUMAN	Apolipoprotein(a) OS=Homo sapiens GN=LPA PE=1 SV=1
sp|A6NFK2|GRCR2_HUMAN	Glutaredoxin domain-containing cysteine-rich protein 2 OS=Homo sapiens GN=G
sp|O95714|HERC2_HUMAN	E3 ubiquitin-protein ligase HERC2 OS=Homo sapiens GN=HERC2 PE=1 SV=2
sp|Q9HCH0|NCK5L_HUMAN	Nck-associated protein 5-like OS=Homo sapiens GN=NCKAP5L PE=1 SV=2
sp|O60885|BRD4_HUMAN	Bromodomain-containing protein 4 OS=Homo sapiens GN=BRD4 PE=1 SV=2
sp|Q9UJX6|ANC2_HUMAN	Anaphase-promoting complex subunit 2 OS=Homo sapiens GN=ANAPC2 PE=1 S
sp|P78563|RED1_HUMAN	Double-stranded RNA-specific editase 1 OS=Homo sapiens GN=ADARB1 PE=1
sp|P06887|LV108_HUMAN	Ig lambda chain V-I region MEM OS=Homo sapiens PE=1 SV=1
sp|Q8N9V7|TOPZ1_HUMAN	Testis- and ovary-specific PAZ domain-containing protein 1 OS=Homo sapiens G
sp|Q9H7S9|ZN703_HUMAN	Zinc finger protein 703 OS=Homo sapiens GN=ZNF703 PE=1 SV=1
sp|O43612|OREX_HUMAN	Orexin OS=Homo sapiens GN=HCRT PE=1 SV=1
sp|Q96JA1|LRIG1_HUMAN	Leucine-rich repeats and immunoglobulin-like domains protein 1 OS=Homo sapie
sp|Q8WXE0|CSKI2_HUMAN	Caskin-2 OS=Homo sapiens GN=CASKIN2 PE=1 SV=2
sp|Q9UHI5|LAT2_HUMAN	Large neutral amino acids transporter small subunit 2 OS=Homo sapiens GN=SL
sp|Q9P2Y4|ZN219_HUMAN	Zinc finger protein 219 OS=Homo sapiens GN=ZNF219 PE=1 SV=2
sp|Q9Y6Z7|COL10_HUMAN	Collectin-10 OS=Homo sapiens GN=COLEC10 PE=2 SV=2
sp|A6NKG5|RTL1_HUMAN	Retrotransposon-like protein 1 OS=Homo sapiens GN=RTL1 PE=3 SV=3
sp|Q9NX46|ARHL2_HUMAN	Poly(ADP-ribose) glycohydrolase ARH3 OS=Homo sapiens GN=ADPRHL2 PE=1
sp|P80108|PHLD_HUMAN	Phosphatidylinositol-glycan-specific phospholipase D OS=Homo sapiens GN=GP
sp|Q8IVE3|PKHH2_HUMAN	Pleckstrin homology domain-containing family H member 2 OS=Homo sapiens G
sp|O00257|CBX4_HUMAN	E3 SUMO-protein ligase CBX4 OS=Homo sapiens GN=CBX4 PE=1 SV=3
sp|Q17RW2|COOA1_HUMAN	Collagen alpha-1(XXIV) chain OS=Homo sapiens GN=COL24A1 PE=1 SV=2
sp|Q13620|CUL4B_HUMAN	Cullin-4B OS=Homo sapiens GN=CUL4B PE=1 SV=4
sp|Q10570|CPSF1_HUMAN	Cleavage and polyadenylation specificity factor subunit 1 OS=Homo sapiens GN=
sp|P36955|PEDF_HUMAN	Pigment epithelium-derived factor OS=Homo sapiens GN=SERPINF1 PE=1 SV=
A0A0B4J1X8|A0A0B4J1X8_HUMAN	Protein IGHV3-43 (Fragment) OS=Homo sapiens GN=IGHV3-43 PE=1 SV=1
sp|Q92859|NEO1_HUMAN	Neogenin OS=Homo sapiens GN=NEO1 PE=1 SV=2
sp|P42356|PI4KA_HUMAN	Phosphatidylinositol 4-kinase alpha OS=Homo sapiens GN=PI4KA PE=1 SV=4
sp|Q7Z2K6|ERMP1_HUMAN	Endoplasmic reticulum metallopeptidase 1 OS=Homo sapiens GN=ERMP1 PE=1
sp|Q5SZK8|FREM2_HUMAN	FRAS1-related extracellular matrix protein 2 OS=Homo sapiens GN=FREM2 PE=
sp|Q9Y6F6|MRVI1_HUMAN	Protein MRVI1 OS=Homo sapiens GN=MRVI1 PE=1 SV=2
sp|Q8TF30|WHAMM_HUMAN	WASP homolog-associated protein with actin membranes and microtubules OS=
sp|Q9BQ75|CMS1_HUMAN	Protein CMSS1 OS=Homo sapiens GN=CMSS1 PE=1 SV=2
sp|B9A064|IGLL5_HUMAN	Immunoglobulin lambda-like polypeptide 5 OS=Homo sapiens GN=IGLL5 PE=2 S
sp|Q5M8T2|S35D3_HUMAN	Solute carrier family 35 member D3 OS=Homo sapiens GN=SLC35D3 PE=2 SV=
sp|O00602|FCN1_HUMAN	Ficolin-1 OS=Homo sapiens GN=FCN1 PE=1 SV=2
sp|Q8TE49|OTU7A_HUMAN	OTU domain-containing protein 7A OS=Homo sapiens GN=OTUD7A PE=1 SV=1
sp|Q9BYP7|WNK3_HUMAN	Serine/threonine-protein kinase WNK3 OS=Homo sapiens GN=WNK3 PE=1 SV=
sp|P00519|ABL1_HUMAN	Tyrosine-protein kinase ABL1 OS=Homo sapiens GN=ABL1 PE=1 SV=4
A0A075B6J7|A0A075B6J7_HUMAN	Protein IGLV3-21 (Fragment) OS=Homo sapiens GN=IGLV3-21 PE=1 SV=1
sp|Q9Y6V0|PCLO_HUMAN	Protein piccolo OS=Homo sapiens GN=PCLO PE=1 SV=4
sp|Q7Z7G1|CLNK_HUMAN	Cytokine-dependent hematopoietic cell linker OS=Homo sapiens GN=CLNK PE=
sp|Q68DK7|MSL1_HUMAN	Male-specific lethal 1 homolog OS=Homo sapiens GN=MSL1 PE=1 SV=3
sp|P20929|NEBU_HUMAN	Nebulin OS=Homo sapiens GN=NEB PE=1 SV=5
sp|Q8N556|AFAP1_HUMAN	Actin filament-associated protein 1 OS=Homo sapiens GN=AFAP1 PE=1 SV=2
sp|Q7Z7L8|CK096_HUMAN	Uncharacterized protein C11orf96 OS=Homo sapiens GN=C11orf96 PE=1 SV=3
sp|P01601|KV109_HUMAN	Ig kappa chain V-I region 16 (Fragment) OS=Homo sapiens GN=IGKV1D-16 P
A0A075B7F0|A0A075B7F0_HUMAN	Protein IGHV3OR16-10 (Fragment) OS=Homo sapiens GN=IGHV3OR16-10 PE=
sp|O00221|IKBE_HUMAN	NF-kappa-B inhibitor epsilon OS=Homo sapiens GN=NFKBIE PE=1 SV=3
sp|Q49AJ0|F135B_HUMAN	Protein FAM135B OS=Homo sapiens GN=FAM135B PE=2 SV=2
sp|Q13207|TBX2_HUMAN	T-box transcription factor TBX2 OS=Homo sapiens GN=TBX2 PE=1 SV=3
sp|Q9UPT6|JIP3_HUMAN	C-Jun-amino-terminal kinase-interacting protein 3 OS=Homo sapiens GN=MAPK
sp|O43516|WIPF1_HUMAN	WAS/WASL-interacting protein family member 1 OS=Homo sapiens GN=WIPF1 I
sp|Q5M9N0|CD158_HUMAN	Coiled-coil domain-containing protein 158 OS=Homo sapiens GN=CCDC158 PE=
sp|Q32P44|EMAL3_HUMAN	Echinoderm microtubule-associated protein-like 3 OS=Homo sapiens GN=EML3
sp|Q9BXP8|PAPP2_HUMAN	Pappalysin-2 OS=Homo sapiens GN=PAPPA2 PE=1 SV=4
sp|Q9Y6X6|MYO16_HUMAN	Unconventional myosin-XVI OS=Homo sapiens GN=MYO16 PE=1 SV=3
Q71RC1|Q71RC1_HUMAN	PP13850 OS=Homo sapiens PE=2 SV=1
sp|Q5TGY3|AHDC1_HUMAN	AT-hook DNA-binding motif-containing protein 1 OS=Homo sapiens GN=AHDC1
A0A075B6I1|A0A075B6I1_HUMAN	Protein IGLV4-60 (Fragment) OS=Homo sapiens GN=IGLV4-60 PE=1 SV=1
sp|A6NEL2|SWAHB_HUMAN	Ankyrin repeat domain-containing protein SOWAHB OS=Homo sapiens GN=SOW
sp|P05452|TETN_HUMAN	Tetranectin OS=Homo sapiens GN=CLEC3B PE=1 SV=3
sp|Q9Y561|LRP12_HUMAN	Low-density lipoprotein receptor-related protein 12 OS=Homo sapiens GN=LRP1
sp|Q9UGU5|HMGX4_HUMAN	HMG domain-containing protein 4 OS=Homo sapiens GN=HMGXB4 PE=1 SV=2
A0A075B7B8|A0A075B7B8_HUMAN	Protein IGHV3OR16-12 (Fragment) OS=Homo sapiens GN=IGHV3OR16-12 PE=
sp|Q6UVK1|CSPG4_HUMAN	Chondroitin sulfate proteoglycan 4 OS=Homo sapiens GN=CSPG4 PE=1 SV=2
sp|Q9UI38|TSP50_HUMAN	Probable threonine protease PRSS50 OS=Homo sapiens GN=PRSS50 PE=1 SV
sp|O75882|ATRN_HUMAN	Attractin OS=Homo sapiens GN=ATRN PE=1 SV=2
sp|Q86UW6|N4BP2_HUMAN	NEDD4-binding protein 2 OS=Homo sapiens GN=N4BP2 PE=1 SV=2
sp|O60287|NPA1P_HUMAN	Nucleolar pre-ribosomal-associated protein 1 OS=Homo sapiens GN=URB1 PE=
sp|Q99698|LYST_HUMAN	Lysosomal-trafficking regulator OS=Homo sapiens GN=LYST PE=1 SV=3

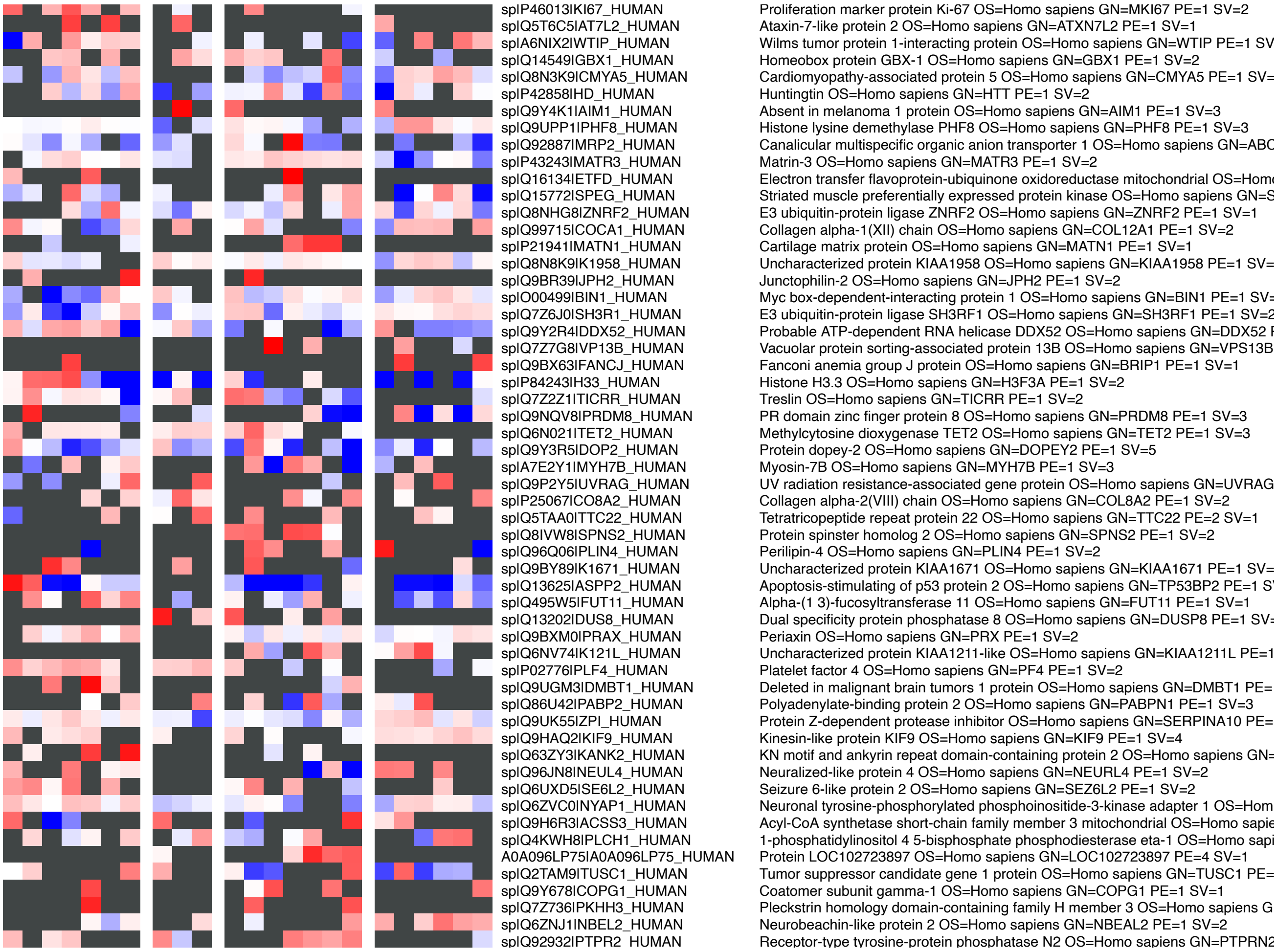
sp|P46013|KI67_HUMAN Proliferation marker protein Ki-67 OS=Homo sapiens GN=MKI67 PE=1 SV=2
sp|Q5T6C5|AT7L2_HUMAN Ataxin-7-like protein 2 OS=Homo sapiens GN=ATXN7L2 PE=1 SV=1
sp|A6NIX2|WTIP_HUMAN Wilms tumor protein 1-interacting protein OS=Homo sapiens GN=WTIP PE=1 SV
sp|Q14549|GBX1_HUMAN Homeobox protein GBX-1 OS=Homo sapiens GN=GBX1 PE=1 SV=2
sp|Q8N3K9|CMYA5_HUMAN Cardiomyopathy-associated protein 5 OS=Homo sapiens GN=CMYA5 PE=1 SV=
sp|P42858|HD_HUMAN Huntingtin OS=Homo sapiens GN=HTT PE=1 SV=2
sp|Q9Y4K1|AIM1_HUMAN Absent in melanoma 1 protein OS=Homo sapiens GN=AIM1 PE=1 SV=3
sp|Q9UPP1|PHF8_HUMAN Histone lysine demethylase PHF8 OS=Homo sapiens GN=PHF8 PE=1 SV=3
sp|Q92887|MRP2_HUMAN Canalicular multispecific organic anion transporter 1 OS=Homo sapiens GN=ABC
sp|P43243|MATR3_HUMAN Matrin-3 OS=Homo sapiens GN=MATR3 PE=1 SV=2
sp|Q16134|ETFD_HUMAN Electron transfer flavoprotein-ubiquinone oxidoreductase mitochondrial OS=Hom
sp|Q15772|SPEG_HUMAN Striated muscle preferentially expressed protein kinase OS=Homo sapiens GN=S
sp|Q8NHG8|ZNRF2_HUMAN E3 ubiquitin-protein ligase ZNRF2 OS=Homo sapiens GN=ZNRF2 PE=1 SV=1
sp|Q99715|COCA1_HUMAN Collagen alpha-1(XII) chain OS=Homo sapiens GN=COL12A1 PE=1 SV=2
sp|P21941|MATN1_HUMAN Cartilage matrix protein OS=Homo sapiens GN=MATN1 PE=1 SV=1
sp|Q8N8K9|K1958_HUMAN Uncharacterized protein KIAA1958 OS=Homo sapiens GN=KIAA1958 PE=1 SV=
sp|Q9BR39|JPH2_HUMAN Junctophilin-2 OS=Homo sapiens GN=JPH2 PE=1 SV=2
sp|O00499|BIN1_HUMAN Myc box-dependent-interacting protein 1 OS=Homo sapiens GN=BIN1 PE=1 SV=
sp|Q7Z6J0|SH3R1_HUMAN E3 ubiquitin-protein ligase SH3RF1 OS=Homo sapiens GN=SH3RF1 PE=1 SV=2
sp|Q9Y2R4|DDX52_HUMAN Probable ATP-dependent RNA helicase DDX52 OS=Homo sapiens GN=DDX52 F
sp|Q7Z7G8|VP13B_HUMAN Vacuolar protein sorting-associated protein 13B OS=Homo sapiens GN=VPS13B
sp|Q9BX63|FANCJ_HUMAN Fanconi anemia group J protein OS=Homo sapiens GN=BRIP1 PE=1 SV=1
sp|P84243|H33_HUMAN Histone H3.3 OS=Homo sapiens GN=H3F3A PE=1 SV=2
sp|Q7Z2Z1|TICRR_HUMAN Treslin OS=Homo sapiens GN=TICRR PE=1 SV=2
sp|Q9NQV8|PRDM8_HUMAN PR domain zinc finger protein 8 OS=Homo sapiens GN=PRDM8 PE=1 SV=3
sp|Q6N021|TET2_HUMAN Methylcytosine dioxygenase TET2 OS=Homo sapiens GN=TET2 PE=1 SV=3
sp|Q9Y3R5|DOP2_HUMAN Protein dopey-2 OS=Homo sapiens GN=DOPEY2 PE=1 SV=5
sp|A7E2Y1|MYH7B_HUMAN Myosin-7B OS=Homo sapiens GN=MYH7B PE=1 SV=3
sp|Q9P2Y5|UVRAG_HUMAN UV radiation resistance-associated gene protein OS=Homo sapiens GN=UVRAG
sp|P25067|CO8A2_HUMAN Collagen alpha-2(VIII) chain OS=Homo sapiens GN=COL8A2 PE=1 SV=2
sp|Q5TAA0|TTC22_HUMAN Tetratricopeptide repeat protein 22 OS=Homo sapiens GN=TTC22 PE=2 SV=1
sp|Q8IVW8|SPNS2_HUMAN Protein spinster homolog 2 OS=Homo sapiens GN=SPNS2 PE=1 SV=2
sp|Q96Q06|PLIN4_HUMAN Perilipin-4 OS=Homo sapiens GN=PLIN4 PE=1 SV=2
sp|Q9BY89|K1671_HUMAN Uncharacterized protein KIAA1671 OS=Homo sapiens GN=KIAA1671 PE=1 SV=
sp|Q13625|ASPP2_HUMAN Apoptosis-stimulating of p53 protein 2 OS=Homo sapiens GN=TP53BP2 PE=1 S
sp|Q495W5|FUT11_HUMAN Alpha-(1 3)-fucosyltransferase 11 OS=Homo sapiens GN=FUT11 PE=1 SV=1
sp|Q13202|DUS8_HUMAN Dual specificity protein phosphatase 8 OS=Homo sapiens GN=DUSP8 PE=1 SV=
sp|Q9BXM0|PRAX_HUMAN Periaxin OS=Homo sapiens GN=PRX PE=1 SV=2
sp|Q6NV74|K121L_HUMAN Uncharacterized protein KIAA1211-like OS=Homo sapiens GN=KIAA1211L PE=1
sp|P02776|PLF4_HUMAN Platelet factor 4 OS=Homo sapiens GN=PF4 PE=1 SV=2
sp|Q9UGM3|DMBT1_HUMAN Deleted in malignant brain tumors 1 protein OS=Homo sapiens GN=DMBT1 PE=
sp|Q86U42|PABP2_HUMAN Polyadenylate-binding protein 2 OS=Homo sapiens GN=PABPN1 PE=1 SV=3
sp|Q9UK55|ZPI_HUMAN Protein Z-dependent protease inhibitor OS=Homo sapiens GN=SERPINA10 PE=
sp|Q9HAQ2|KIF9_HUMAN Kinesin-like protein KIF9 OS=Homo sapiens GN=KIF9 PE=1 SV=4
sp|Q63ZY3|KANK2_HUMAN KN motif and ankyrin repeat domain-containing protein 2 OS=Homo sapiens GN=
sp|Q96JN8|NEUL4_HUMAN Neuralized-like protein 4 OS=Homo sapiens GN=NEURL4 PE=1 SV=2
sp|Q6UXD5|SE6L2_HUMAN Seizure 6-like protein 2 OS=Homo sapiens GN=SEZ6L2 PE=1 SV=2
sp|Q6ZVC0|NYAP1_HUMAN Neuronal tyrosine-phosphorylated phosphoinositide-3-kinase adapter 1 OS=Hom
sp|Q9H6R3|ACSS3_HUMAN Acyl-CoA synthetase short-chain family member 3 mitochondrial OS=Homo sapie
sp|Q4KWH8|PLCH1_HUMAN 1-phosphatidylinositol 4 5-bisphosphate phosphodiesterase eta-1 OS=Homo sapi
A0A096LP75|A0A096LP75_HUMAN Protein LOC102723897 OS=Homo sapiens GN=LOC102723897 PE=4 SV=1
sp|Q2TAM9|TUSC1_HUMAN Tumor suppressor candidate gene 1 protein OS=Homo sapiens GN=TUSC1 PE=
sp|Q9Y678|COPG1_HUMAN Coatomer subunit gamma-1 OS=Homo sapiens GN=COPG1 PE=1 SV=1
sp|Q7Z736|PKHH3_HUMAN Pleckstrin homology domain-containing family H member 3 OS=Homo sapiens G
sp|Q6ZNJ1|NBEL2_HUMAN Neurobeachin-like protein 2 OS=Homo sapiens GN=NBEAL2 PE=1 SV=2
sp|Q92932|PTPR2_HUMAN Receptor-type tyrosine-protein phosphatase N2 OS=Homo sapiens GN=PTPRN2
