# Supplementary material for: Plasma enzymatic activity, proteomics and peptidomics in COVID-19-induced sepsis: A novel approach for the analysis of hemostasis
Source: Front Mol Biosci. 2023 Jan 11;9:1051471. doi: 10.3389/fmolb.2022.1051471 (PMC9874325; doi:10.3389/fmolb.2022.1051471)
Supplement: Supplementary file 2 [file DataSheet3.PDF]

SUPPLEMENTARY FILE.

S1. Clinical data file.

Because these are observational data, there are some data points not reported, either because the patient was discharged from the unit, the measurements were not collected (particularly laboratory values) or were not reported. Abbreviations are listed at the base of the Table.

Age	57	47	88	29
Sex	M	M	F	M
Ethnicity	Hispanic	Asian/Indian	Hispanic	Hispanic
Height (cm)	167	172	160	160
Weight (kg)	82	70	82.3	77.3
BMI	29.4	23.7	32.1	30.2
Predicted body weight (kg)	63.2	67.7	52.4	56.9
**Premorbid Conditions**				
Cardiovascular disease		HTN	HTN	HTN
Respiratory disease				
Neurologic disease				
Chronic kidney disease				
Diabetes Mellitus				
Liver disease				
Rheumatologic disease				Y
Status/post organ transplant				
Cancer (present or history)				
**Presenting Symptoms**				
Fever	Y	Y	Y	Y
Cough	Y	Y	Y	
Nausea/vomiting	Y	Y		
Diarrhea				Y
Abdominal pain	Y			
Anorexia				Y
Shortness of breath	Y	Y	Y	
Chest pain				
Fatigue/Malaise	Y	Y		Y
Myalgias		Y		Y
Alcohol/smoker/drugs				
ICU admit day (hospital admit day = 0)	1	0	2	0
Admit from Emergency Department	Y		Y	
Frailty - very fit	Good health	Good health	Healthy	Good health
Modified Rankin score - baseline	0	0	0	1
Cerebral performance good	Y	Y	Y	Y
Outpatient meds: ASA			Y	Y
Outpatient meds: statin		Y		
Outpatient meds: ACEI			Y	
Outpatient meds: ARB				Y
Outpatient meds: inhaled steroid				Y
Outpatient meds: metformin				Y
Outpatient meds: none of the above	Y			

Immune: Mycophenolate mofetil				Y
No immune meds	Y	Y	Y	
**Treatment Medications**				
IV antibiotics	Y	Y	Y	Y
Azithromycin	Y	Y	Y	Y
Azithromycin start day	0	0	0	0
Azithromycin end day	2	2	0	2
Ceftriaxone	Y	Y		
Ceftriaxone start day	0	0		
Ceftriaxone end day	2	2		
Meropenem			Y	
Meropenem start day			15	
Meropenem end day			17	
Vancomycin			Y	Y
Vancomycin start day			16	0
Vancomycin end day			17	2
Piperacillin/Tazobactam (Zosyn)			Y	Y
Zosyn start day			7	0
Zosyn end day			15	3
**Infections**				
+ urine culture			Y	
Date + culture was reported			6	
Urine Pseudomonas			Y	
Respiratory infection - intubated			Y	
Date + culture was reported			7	
Pulmonary enterobacter			Y	
Respiratory infection - intubated			Y	
Date + culture was reported			12	
Pulmonary enterobacter			Y	
+ urine culture			Y	
Date + culture was reported			12	
Urine - Candida Albicans			Y	
Respiratory viral panel + (COVID)	Y	Y	Y	Y
**Clinical Course**				
Shock with hypotension	Y		Y	Y
Myocardial depression			Y	
Vasopressors required	Y		Y	Y
ARDS	Y		Y	Y
COVID only respiratory cause	Y			Y
+ Bacterial pneumonia			Y	
**Pulmonary**				
Oxygen requirement start (no ETT)		0		
Oxygen requirement end (no ETT)		6		
Days intubated	11	0	13	6

Day 0 Respiratory Rate		23	26	38
Day 1 Respiratory Rate		22	28	25
Day 2 Respiratory Rate	33	25	11	23
Day 3 Respiratory Rate	30	35	22	24
Day 4 Respiratory Rate	28	18	26	25
Day 5 Respiratory Rate	28	20	26	28
Day 6 Respiratory Rate	27		28	34
Day 7 Respiratory Rate	16		32	31
Day 8 Respiratory Rate	27		30	
Day 0 Respiratory device	Nasal cannula	Non-rebreather	Nasal cannula	CPAP/BiPAP
Day 1 Respiratory device	Non-rebreather	Nasal cannula	Non-rebreather	ET Tube
Day 2 Respiratory device	Non-rebreather	Nasal cannula	ET Tube	ET Tube
Day 3 Respiratory device	Non-rebreather	Non-rebreather	ET Tube	ET Tube
Day 4 Respiratory device	ET Tube	Nasal cannula	ET Tube	ET Tube
Day 5 Respiratory device	ET Tube	0	ET Tube	ET Tube
Day 6 Respiratory device	ET Tube		ET Tube	0
Day 7 Respiratory device	ET Tube		ET Tube	0
Day 8 Respiratory device	Nasal cannula		Nasal cannula	
Day 0 liters/min	2	15	6	
Day 1 liters/min	15	6	12	
Day 2 liters/min	15	6		
Day 3 liters/min	15	15		
Day 4 liters/min		5		
Day 7 liters/min	1			
Day 8 liters/min	1		3	
Day 0 FiO2				15
Day 1 FiO2				80
Day 2 FiO2			70	80
Day 3 FiO2			60	50
Day 4 FiO2	60		55	45
Day 5 FiO2	50		50	40
Day 6 FiO2	40		80	
Day 7 FiO2			45	

Ventilator parameters

Day 1 Ventilator mode				VTPC
Day 2 Ventilator mode			VTPC	VTPC
Day 3 Ventilator mode			VTPC	VTPC
Day 4 Ventilator mode	VTPC*		VTPC	VTPC
Day 5 Ventilator mode	VTPC		VTPC	VTPC
Day 6 Ventilator mode	VTPC		VTPC	
Day 7 Ventilator mode			VTPC	

*Volume Targeted Pressure Control

DAY 1

Day 1 Set respiratory rate [seconds]				26
Day 1 Actual respiratory rate [seconds]				32

Day 1 Set volume [ml]			350
Day 1 Actual volume [ml]			315
Day 1 Minute ventilation [liters]			10.1
Day 1 Peak inspiratory pressure (PIP) cmH2O			25
Day 1 PEEP [cmH2O]			10
Day 1 Inspiratory time [seconds]			0.8
Day 1 Plateau pressure [cmH2O]			24
Day 1 FiO2 on ABG [%]			80
**DAY 2**			
Day 2 Set respiratory rate [seconds]		22	26
Day 2 Actual respiratory rate [seconds]		22	26
Day 2 Set volume [ml]		310	350
Day 2 Actual volume [ml]		338	316
Day 2 Minute ventilation [liters]		5.32	8.5
Day 2 Peak inspiratory pressure (PIP) cmH2O		24	29
Day 2 PEEP [cmH2O]		14	14
Day 2 Inspiratory time [seconds]		0.9	0.85
Day 2 Plateau pressure [cmH2O]		24	
Day 2 FiO2 on ABG [%]		100	80
**DAY 3**			
Day 3 Set respiratory rate [seconds]		22	24
Day 3 Actual respiratory rate [seconds]		22	24
Day 3 Set volume [ml]		310	350
Day 3 Actual volume [ml]		314	352
Day 3 Minute ventilation [liters]		6.87	8.46
Day 3 Peak inspiratory pressure (PIP) cmH2O		28	29
Day 3 PEEP [cmH2O]		10	14
Day 3 Inspiratory time [seconds]		0.9	0.85
Day 3 FiO2 on ABG [%]			50
**Day 4**			
Day 4 Set respiratory rate [seconds]		44	46
Day 4 Actual respiratory rate [seconds]		44	46
Day 4 Set volume [ml]		310	350
Day 4 Actual volume [ml]		338	316
Day 4 Minute ventilation [liters]		5.34	8.5
Day 4 Peak inspiratory pressure (PIP) cmH2O		44	49
Day 4 PEEP[cmH2O]		14	14
Day 4 Inspiratory time [seconds]		0.9	0.85
Day 4 Plateau pressure [cmH2O]		44	
Day 4 FiO2 on ABG [%]		100	80
**DAY 5**			
Day 5 Set respiratory rate [seconds]	28	30	24
Day 5 Actual respiratory rate [seconds]	28	30	26
Day 5 Set volume [ml]	400	300	350
Day 5 Actual volume [ml]	401	302	357

Day 5 Minute ventilation [liters]	11.2	9.04	9.6
Day 5 Peak inspiratory pressure (PIP) cmH2O	28	28	20
Day 5 PEEP [cmH2O]	12	15	5
Day 5 Inspiratory time [seconds]		0.85	0.85
Day 5 Plateau pressure [cmH2O]	26		27
Day 5 FiO2 on ABG [%]	50	50	40
**DAY 6**			
Day 6 Set respiratory rate [seconds]	24	28	
Day 6 Actual respiratory rate [seconds]	27	28	
Day 6 Set volume [ml]	400	300	
Day 6 Actual volume [ml]	382	306	
Day 6 Minute ventilation [liters]	11	8.51	
Day 6 Peak inspiratory pressure (PIP) cmH2O	18	32	
Day 6 PEEP [cmH2O]	8	12	
Day 6 Inspiratory time [seconds]	0.8	0.85	
Day 6 Plateau pressure [cmH2O]	22		
Day 6 FiO2 on ABG [%]		60	
**DAY 7**			
Day 6 Set respiratory rate [seconds]		28	
Day 6 Actual respiratory rate [seconds]		32	
Day 6 Set volume [ml]		300	
Day 6 Actual volume [ml]		289	
Day 6 Minute ventilation [liters]		9.29	
Day 6 Peak inspiratory pressure (PIP) cmH2O		24	
Day 6 PEEP [cmH2O]		10	
Day 6 Inspiratory time [seconds]		0.85	
Day 6 FiO2 on ABG [%]		45	
**Arterial blood gases (ABGs)**			
Day 0 pH		7.42	
Day 1 pH			7.31
Day 2 pH		7.31	7.3
Day 3 pH			7.42
Day 4 pH	7.4	7.37	7.33
Day 5 pH	7.47	7.3	7.41
Day 6 pH		7.46	
Day 8 pH		7.47	
Day 0 PaCO2 [mmHg]		37	
Day 1 PaCO2 [mmHg]			44
Day 2 PaCO2 [mmHg]		47	45
Day 3 PaCO2 [mmHg]			34
Day 4 PaCO2 [mmHg]	55	43	42
Day 5 PaCO2 [mmHg]	53	52	38
Day 6 PaCO2 [mmHg]		56	
Day 8 PaCO2 [mmHg]		34	

Day 0 PaO2 [mmHg]			73	
Day 1 PaO2 [mmHg]				80
Day 2 PaO2 [mmHg]			59	98
Day 3 PaO2 [mmHg]				104
Day 4 PaO2 [mmHg]	79		65	97
Day 5 PaO2 [mmHg]	101		71	104
Day 6 PaO2 [mmHg]			170	
Day 8 PaO2 [mmHg]			79	
Neuromuscular blockade	Y		Y	Y
Prone positioning	Y		Y	Y
**SPO2**				
Day 0 SpO2 [%]	95	98	84	99
Day 1 SpO2 [%]		93	97	100
Day 2 SpO2 [%]	94	97	93	96
Day 3 SpO2 [%]	100	90	99	100
Day 4 SpO2 [%]	98	94	98	95
Day 5 SpO2 [%]	100	96	96	96
Day 6 SpO2 [%]	96		100	
Day 7 SpO2 [%]	97		97	97
Day 8 SpO2 [%]	93		100	
**Neurologic**				
Day 1 RASS	0	0	0	-2
Day 2 RASS	0	0	-2	-2
Day 3 RASS	0	0	-4	-5
Day 4 RASS	-5	0	-5	-2
Day 5 RASS	-5	0	-5	-1
Day 6 RASS	-3		-5	0
Day 7 RASS	0		-4	0
**Sedation**				
Day 6 Dexmedetomidine (mcg/kg/hr)	0.3			
Day 1 Fentanyl rate (mcg/hr)				75
Day 2 Fentanyl rate (mcg/hr)			100	150
Day 3 Fentanyl rate (mcg/hr)	0	0	100	150
Day 4 Fentanyl rate (mcg/hr)	150		125	150
Day 5 Fentanyl rate (mcg/hr)	200		125	100
Day 6 Fentanyl rate (mcg/hr)			100	
Day 1 Propofol rate (mcg/kg/min)				60
Day 2 Propofol rate (mcg/kg/min)			40	60
Day 3 Propofol rate (mcg/kg/min)	0	0	40	60
Day 4 Propofol rate (mcg/kg/min)	40		40	30
Day 5 Propofol rate (mcg/kg/min)	60		40	30

Day 2 Midazolam (mg/hr)				3
Day 3 Midazolam (mg/hr)	0	0	0	4
Day 4 Midazolam (mg/hr)			5	4
Day 5 Midazolam (mg/hr)	2		5	2
Day 0 sedation	None	None	None	None
Day 1 sedation	None	None	None	
Day 2 sedation	None	None		
Day 3 sedation	None	None		
Day 4 sedation		None		
Day 6 sedation			None	None
Day 7 sedation	None		None	None
Day 8 sedation	None			
**SOFA scores**				
Day 0 SOFA score	0	3	0	0
Day 1 SOFA score	0		8	8
Day 2 SOFA score	1	0	8	8
Day 3 SOFA score	0	0	8	9
Day 4 SOFA score	9	0	8	8
Day 5 SOFA score	8	0	8	8
Day 6 SOFA score	8		8	0
Day 7 SOFA score	1		8	3
Day 8 SOFA score	3		3	
**Temperature (°C)**				
Day 0 Temperature	39.4	37.7	39.2	36.9
Day 1 Temperature	37.7	36.8	37.1	38.9
Day 2 Temperature	38.5	36.8	37.5	37.8
Day 3 Temperature	38.2	38.5	36.4	36.5
Day 4 Temperature	36.5	36.9	36.3	38.1
Day 5 Temperature	36.3	36.8	37.1	37.8
Day 6 Temperature	37.5		36.9	36.3
Day 7 Temperature	37		37.8	37.8
Day 8 Temperature	37.3		37	
**Hemodynamics**				
**Heart rate (bpm)**				
Day 0 Heart rate	81	104	79	100
Day 1 Heart rate	88	101	73	97
Day 2 Heart rate	89	99	89	84
Day 3 Heart rate	96	114	93	62
Day 4 Heart rate	72	85	62	95
Day 5 Heart rate	68	102	98	110
Day 6 Heart rate	79		62	88
Day 7 Heart rate	123		71	71
Day 8 Heart rate	125		89	
**Systolic Blood Pressure (mmHg)**				

Day 0 Systolic Blood Pressure	122	142	154	115
Day 1 Systolic Blood Pressure	139	113	122	99
Day 2 Systolic Blood Pressure	141	121	153	109
Day 3 Systolic Blood Pressure	141	139	119	100
Day 4 Systolic Blood Pressure	120	128	144	114
Day 5 Systolic Blood Pressure	129	127	174	124
Day 6 Systolic Blood Pressure	111		157	123
Day 7 Systolic Blood Pressure	134		133	115
Day 8 Systolic Blood Pressure	160		74	
**Diastolic Blood Pressure (mmHg)**				
Day 0 Diastolic Blood Pressure	69	83	78	59
Day 1 Diastolic Blood Pressure	71	85	53	59
Day 2 Diastolic Blood Pressure	71	74	55	53
Day 3 Diastolic Blood Pressure	74	81	43	54
Day 4 Diastolic Blood Pressure	49	75	52	61
Day 5 Diastolic Blood Pressure	76	72	75	86
Day 6 Diastolic Blood Pressure	50		60	73
Day 7 Diastolic Blood Pressure	85		49	73
Day 8 Diastolic Blood Pressure	64		34	
**Mean Blood Pressure (MAP) (mmHg)**				
Day 0 MAP	79	96	102	71
Day 1 MAP	91	92	68	71
Day 2 MAP	88	86	87	71
Day 3 MAP	89	92	69	63
Day 4 MAP	69	88	88	78
Day 5 MAP	86	103	115	86
Day 6 MAP	68		96	82
Day 7 MAP	96		79	82
Day 8 MAP	78		47	
**Pressor Requirements**				
Pressor required during ICU stay	Y		Y	Y
Number of days requiring pressor	11		15	5
Day 1 Norepinephrine (mcg/min)	0	0	0	1
Day 2 Norepinephrine (mcg/min)	0	0	1	1
Day 3 Norepinephrine (mcg/min)	0	0	7	0
Day 4 Norepinephrine (mcg/min)	4	0	0	0
Day 5 Norepinephrine (mcg/min)	7	0	8	0
Day 6 Norepinephrine (mcg/min)	4	0	0	0
Day 5 Vasopressin (U/min)	0	0	0.04	0
Day 8 Vasopressin (U/min)	0	0	0.04	0
Day 4 Phenylephrine (mcg/min)	0	0	55	0
Day 6 Phenylephrine (mcg/min)			20	
**Feeding and I&Os [mL]**				

Enteral Feeding start (day)	1	1	0	0
Day 0 Total in	800	740	579	1461
Day 1 Total in	106	1350		2615
Day 2 Total in	960	395	1471	2056
Day 3 Total in			2485	2190
Day 4 Total in	3043	513	2161	
Day 5 Total in		637	4028	
Day 7 Total in	2609			
Day 8 Total in	1450			
Day 0 Total out	800	1375	891	1575
Day 1 Total out	2020	2000		2175
Day 2 Total out	1235	1008	3052	1425
Day 3 Total out			920	2195
Day 4 Total out	3510	1250	3575	
Day 5 Total out			4770	
Day 7 Total out	652			
Day 8 Total out	625			
Day 0 Urine out	800	1375	890	1575
Day 1 Urine out	2020	2000		2175
Day 2 Urine out	1235	1008	3052	1425
Day 3 Urine out			920	1895
Day 4 Urine out	3510	1250	3575	
Day 5 Urine out			4770	
Day 7 Urine out	650			
Day 8 Urine out	625			
**Laboratory Data**				
**White blood cell count (WBC) [1000/mcl]**				
Day 0 WBC	10.6	9.6	8.1	8.5
Day 1 WBC	9.2	8	8.3	10.3
Day 2 WBC	9.7	8.2		7.4
Day 3 WBC	7.6	8.1	12.7	5.7
Day 4 WBC	11.8	8.8	10	6
Day 5 WBC	9.5	7.4	10.8	8.1
Day 6 WBC	12.3			
Day 7 WBC	11.2		11.2	
Day 8 WBC			19.8	
Average WBC	10.2	8.4	11.6	7.7
Standard Deviation WBC	1.5	0.8	4.0	1.7
Day 0 Neutrophil count (%)	88	77	63	75
Day 1 Neutrophil count (%)	84	52	97	82
Day 2 Neutrophil count (%)	87	66		73
Day 3 Neutrophil count (%)	87	87	88	64
Day 4 Neutrophil count (%)	87	71	82	82
Day 5 Neutrophil count (%)	82		67	84

Day 6 Neutrophil count (%)	90			
Day 7 Neutrophil count (%)	77		84	
Day 8 Neutrophil count (%)			85	
Day 0 Lymphocyte count (%)	8	12	28	8
Day 1 Lymphocyte count (%)	10	17	0	8
Day 2 Lymphocyte count (%)	8	16		12
Day 3 Lymphocyte count (%)	7	7	8	16
Day 4 Lymphocyte count (%)	7	8	11	11
Day 5 Lymphocyte count (%)	9		21	6
Day 6 Lymphocyte count (%)	4			
Day 7 Lymphocyte count (%)	13		10	
Day 8 Lymphocyte count (%)			6	
**Hemoglobin (Hb) [g/dL]**				
Day 0 Hb	15.4	15.3	11	14.2
Day 1 Hb	14	15.3	10	12.8
Day 2 Hb	13.9	14.6		11.3
Day 3 Hb	12.5	12.1	10.5	10.6
Day 4 Hb	11.9	13.5	9.3	10.1
Day 5 Hb	11.7	13.6	10.5	10.3
Day 6 Hb	12			
Day 7 Hb	12.7		8.2	
Day 8 Hb			6.4	
Hb Average	13.0	14.1	9.4	11.6
Hb Standard Deviation	1.3	1.2	1.6	1.6
**Platelets [/mcL]**				
Day 0 Platelets	223	179	173	175
Day 1 Platelets	339	183	170	183
Day 2 Platelets	405	237		163
Day 3 Platelets	416	438	248	165
Day 4 Platelets	481	302	209	113
Day 5 Platelets	468	461	265	122
Day 6 Platelets	456			
Day 7 Platelets	446		391	
Day 8 Platelets			608	
Platelets Average	404.3	300.0	294.9	153.5
Platelets Standard Deviation	85.9	124.3	157.2	28.9
**Coagulation panel**				
Day 0 INR			1.2	
Day 1 INR	1.2	1.2	1.4	
Day 2 INR	1.3	1.1		
Day 3 INR	1.4	1.4	1.4	
Day 4 INR	1.4	1.1	1.41	
Day 5 INR	1.3			
Day 6 INR	1.2			
Day 7 INR	1.3			

Day 8 INR	1.4			
INR Average	1.31	1.20	1.40	
INR Standard Deviation	0.08	0.14	0.01	
Day 0 PTT			31	
Day 4 PTT			70	
Day 0 D-dimer	354		699	
<u>**Chemistry panel**</u>				
**Na+ [mEq/L]**				
Day 0 Sodium	133	138	139	137
Day 1 Sodium	139	141	142	138
Day 2 Sodium	141	139	145	143
Day 4 Sodium	137	140	143	147
Day 5 Sodium	141	141	149	145
Day 6 Sodium	140		146	
Day 7 Sodium	147		143	
Day 8 Sodium	134		148	
**K+ [mEq/L]**				
Day 0 Potassium	4.4	3.5	5.1	3.4
Day 1 Potassium	4.1	4.1	3.8	4.2
Day 2 Potassium	4.1	3.6	4.1	4.7
Day 3 Potassium	3.8	3.8	4	3.8
Day 4 Potassium	4.1	3.3	3.8	3.6
Day 5 Potassium	3.7	4.3	3.9	4
Day 6 Potassium	4		3.2	
Day 7 Potassium	3.8		3.1	
Day 8 Potassium	3.8		4.5	
**Cl- [mM/L]**				
Day 0 Chloride	92	101	103	98
Day 1 Chloride	94	102	106	102
Day 2 Chloride	95	100	109	108
Day 3 Chloride	93	99	107	111
Day 4 Chloride	96	103	107	113
Day 5 Chloride	95	104	111	112
Day 6 Chloride	92		101	
Day 7 Chloride	99		102	
Day 8 Chloride	92		111	
**Bicarbonate [mM/L]**				
Day 0 Bicarbonate	24	22	19	20
Day 1 Bicarbonate	30	23	21	18
Day 2 Bicarbonate	28	24	19	20
Day 3 Bicarbonate	31	26	24	21
Day 4 Bicarbonate	26	23	22	21
Day 5 Bicarbonate	31	23	24	21
Day 6 Bicarbonate	35		31	

Day 7 Bicarbonate	31		25	
Day 8 Bicarbonate	29		22	
**BUN (Blood urea nitrogen) [mg/dL]**				
Day 0 BUN	10	6	35	14
Day 1 BUN	15	12	15	20
Day 2 BUN	23	14	24	32
Day 3 BUN	139	138	146	146
Day 3 BUN	21	16	20	28
Day 4 BUN	20	11	43	42
Day 5 BUN	22	11	54	31
Day 6 BUN	25		48	
Day 7 BUN	35		49	
Day 8 BUN	15		77	
**Creatinine [mg/dL]**				
Day 0 Creatinine	0.81	0.57	1.03	1.11
Day 1 Creatinine	0.75	0.66	0.84	1.28
Day 2 Creatinine	0.66	0.67	0.76	1.37
Day 3 Creatinine	0.6	0.47	0.82	1.17
Day 4 Creatinine	0.67	0.57	1.13	1.02
Day 5 Creatinine	0.79	0.7	1.05	0.82
Day 6 Creatinine	0.76		0.95	
Day 7 Creatinine	0.71		0.79	
Day 8 Creatinine	0.59		1.02	
**Glucose [mg/dL]**				
Day 0 Glucose	114	95	101	126
Day 1 Glucose	120	105	95	168
Day 2 Glucose	115	103	115	116
Day 3 Glucose	97	114	98	94
Day 4 Glucose	136	131	92	133
Day 5 Glucose	224	113	145	128
Day 6 Glucose	174		147	
Day 7 Glucose	104		283	
Day 8 Glucose	143		244	
**Calcium [mg/dL]**				
Day 0 Calcium	8.8	8.6	8.8	8.7
Day 1 Calcium	9.2	8.5	8.6	8.6
Day 2 Calcium	9.3	8.7	8.6	8.4
Day 3 Calcium	9	7.9	8.37	8.7
Day 4 Calcium	8.7	8.5	7.9	8.3
Day 5 Calcium	8.3	9	8.2	9.1
Day 6 Calcium	8.9		8.4	
Day 7 Calcium	9.6		9.3	
Day 8 Calcium	8.5		8.5	
**Alkaline Phosphatase (AP) [IU/L]**				

Day 0 AP	101	118	88	105
Day 1 AP	88	119	73	
Day 2 AP	103	122	64	
Day 3 AP	91	79	67	
Day 4 AP	86	136	75	
Day 5 AP	123	136	91	
Day 6 AP	126			
Day 7 AP	134		118	
Day 8 AP	119		91	
**Alanine aminotransferase (ALT) [IU/L]**				
Day 0 ALT	37	30	21	23
Day 1 ALT	35	43	17	
Day 2 ALT	34	60	15	
Day 3 ALT	28	20	14	
Day 4 ALT	22	106	11	
Day 5 ALT	76	124	14	
Day 6 ALT	100			
Day 7 ALT	87		40	
Day 8 ALT	40		43	
**Aspartate aminotransferase (AST) [IU/L]**				
Day 0 AST	68	44	57	24
Day 1 AST	62	62	57	
Day 2 AST	57	58	45	
Day 3 AST	58	42	45	
Day 4 AST	42	69	34	
Day 5 AST	109	72	44	
Day 6 AST	105			
Day 7 AST	58		61	
Day 8 AST	34		43	
**Total Bilirubin (T Bili) [mg/dL]**				
Day 0 T Bili	0.7	0.4	0.4	0.5
Day 1 T Bili	0.7	0.4	0.5	
Day 2 T Bili	1	0.7	0.4	
Day 3 T Bili	1	1	0.5	
Day 4 T Bili	1.2	0.7	0.6	
Day 5 T Bili	0.8	0.3	0.9	
Day 6 T Bili	0.4			
Day 7 T Bili	1		0.8	
Day 8 T Bili	1.1		0.8	
**Albumin [g/dL]**				
Day 0 Albumin	3.6	3.6	3.5	3.6
Day 1 Albumin	2.9	3.5	3.2	
Day 2 Albumin	2.9	3.3	2.8	
Day 3 Albumin	2.6	2	2.5	
Day 4 Albumin	2.3	3	2	

Day 5 Albumin	2.1	3.3	2.2	
Day 6 Albumin	2.4			
Day 7 Albumin	2.8		2.4	
Day 8 Albumin	2.6		2.3	
**Total Protein (TP) [g/dL]**				
Day 0 TP	7.3	7.3	8.1	7.2
Day 1 TP	6.9	7	7.6	
Day 2 TP	7.2	7.2	6.8	
Day 3 TP	6.8	5.9	6.8	
Day 4 TP	6.4	6.6	6.2	
Day 5 TP	6.4	6.9	7	
Day 7 TP	8.6		7.5	
Day 8 TP	6.9		7.1	
**Phosphate (Phos) [mg/dL]**				
Day 0 Phos	3.6			
Day 1 Phos	4.3		3.4	
Day 2 Phos	3.7		3	3.3
Day 3 Phos	3.9	3.4		3.1
Day 4 Phos	4.1			3.2
Day 5 Phos	3.9			3.4
Day 6 Phos	4.7		3.2	
Day 7 Phos	5.3			
**Magnesium (Mg) [mM/L]**				
Day 0 Mg	2.2			
Day 1 Mg	2.5	2	2	
Day 2 Mg	2.5	2	2	2.5
Day 3 Mg	2.4	2	1.8	2.7
Day 4 Mg	2.3	2.1	1.9	2.6
Day 5 Mg	2		2.3	2.4
Day 6 Mg	2.2		2.2	
Day 7 Mg	2.4		2.4	
Day 8 Mg			3	
**Procalcitonin [ng/mL]**				
Day 0 Procalcitonin	0.23	0.09	0.17	0.45
Day 1 Procalcitonin			0.32	
**Lactate [mg/dL]**				
Day 0 Lactate	1.3	0.9	1.9	1.3
Day 2 Lactate			1.7	
**Miscellaneous Labs**				
Day 0 Lactate dehydrogenase	468	493	722	
Day 0 Creatinine phosphokinase [mcg/dL]			922	
Day 0 Brain natriuretic peptide [pg/mL]	0		1159	1161
Day 0 C-reactive protein [mg/L]			11.87	

Day 0 Erythrocyte sedimentation rate [mm/hr]				
Day 0 Ferritin [mcg/L]	1236	2957	500	
Day 0 Troponin (High Sensitivity) [ng/L]		0	41	25
Day 0 Interleukin-6 [pg/mL]			23	
**Discharge Information**				
Survive to discharge	1	1	0	1
Hospital Length of stay	23	8	18	11
ICU Length of stay	17	3	18	9

Abbreviations used and not previously defined: ACEI, angiotensin converting enzyme inhibitor; ARB, angiotensin 2 receptor blocker; ARDS, Acute Respiratory Distress Syndrome; ASA, aspirin; CPAP/BiPAP, continuous positive airway pressure/bilevel positive airway pressure; ET tube, endotracheal tube; bpm, beats-per-minute; INR, international normalized ratio; mmHg, millimeters (of) mercury; PEEP, positive end-expiratory pressure; RASS, Richmond Agitation Sedation Scale; SOFA, Sequential Organ Failure Assessment; VTPC, volume targeted pressure control (ventilator mode).

Continuous outcomes reported as a single number reflect the median of the 24-hour period. Notation on prone positioning and use of neuromuscular blockers indicate whether they were employed during the patients' ICU course. These subjects were enrolled early in the COVID pandemic, before the widespread use of steroids and anti-viral therapies.
